# Supplementary material for: Genetic interaction between Adgrg6 and Sox9 reveals a feedforward mechanism for postnatal spinal stability
Source: Res Sq. 2026 Apr 8:rs.3.rs-9271349. Preprint. [Version 1] doi: 10.21203/rs.3.rs-9271349/v1 (PMC13082157; doi:10.21203/rs.3.rs-9271349/v1)
Supplement: Supplement 1 [file NIHPPrs9271349v1-supplement-1.pdf]

Supplemental Figures

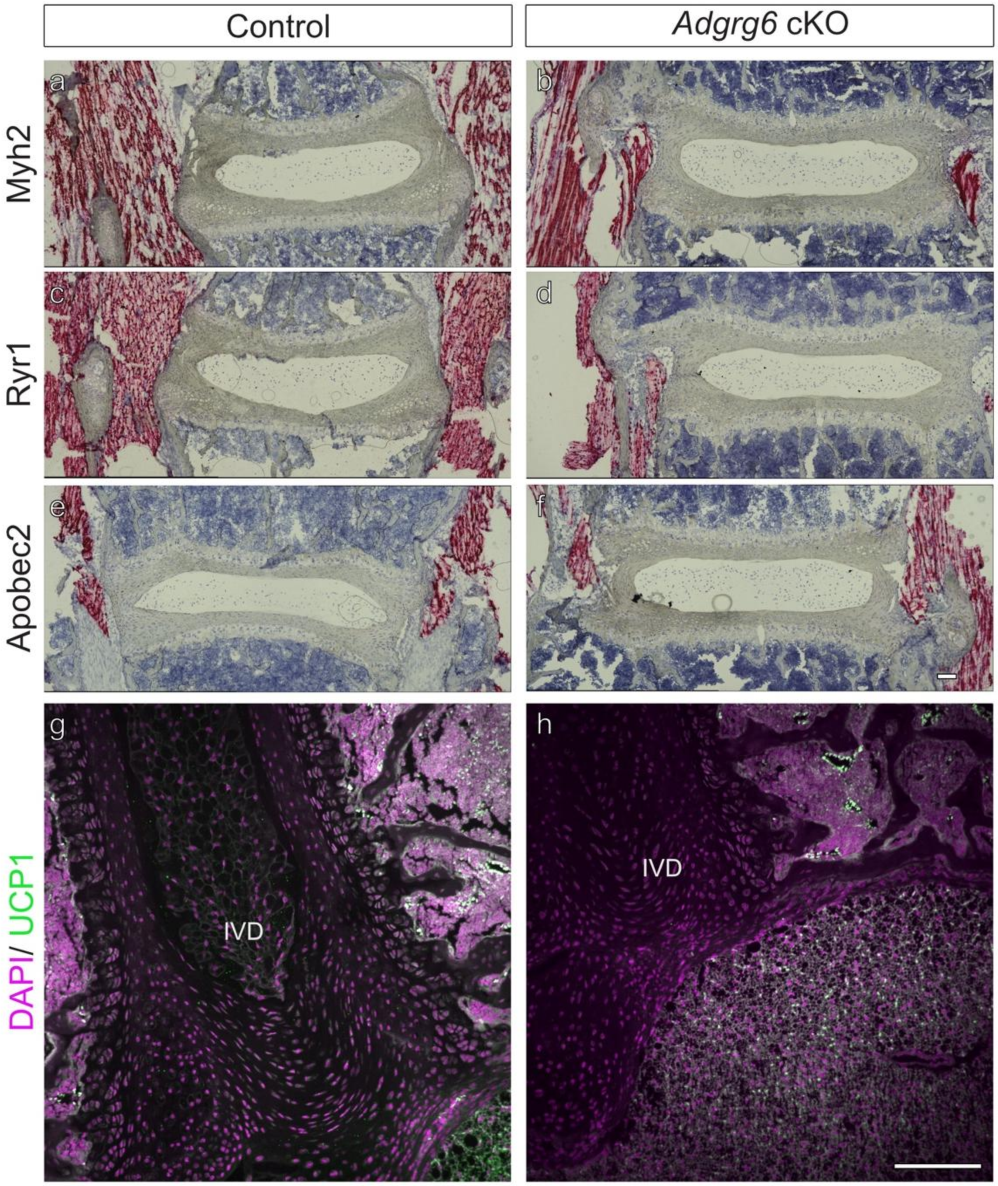

Supplemental Figure 1. RNA *in situ* analysis of muscle and adipose-associated transcripts. Representative RNAScope images comparing control and *Adgrg6-cKO* spines probed for muscle genes *Myh2* (a-b), *Ryr1* (c-d), and *Apobec2* (e-f). Red puncta indicate transcript signal, which is robust in the paraspinal skeletal muscle adjacent to the vertebral column in both genotypes. No detectable expression of *Myh2* or *Ryr1* is observed within the IVD (a-d). In contrast, *Apobec2* shows very low-level signal in the annulus fibrosus. UCP1 expression (green) in the paraspinal adipose and muscle tissues in wild-type control (g) and *Adgrg6-cKO* mutant (h) mice, contrasted with nuclei staining to highlight the intervertebral disc (IVD) (DAPI/magenta). Scale bars = 100 um.

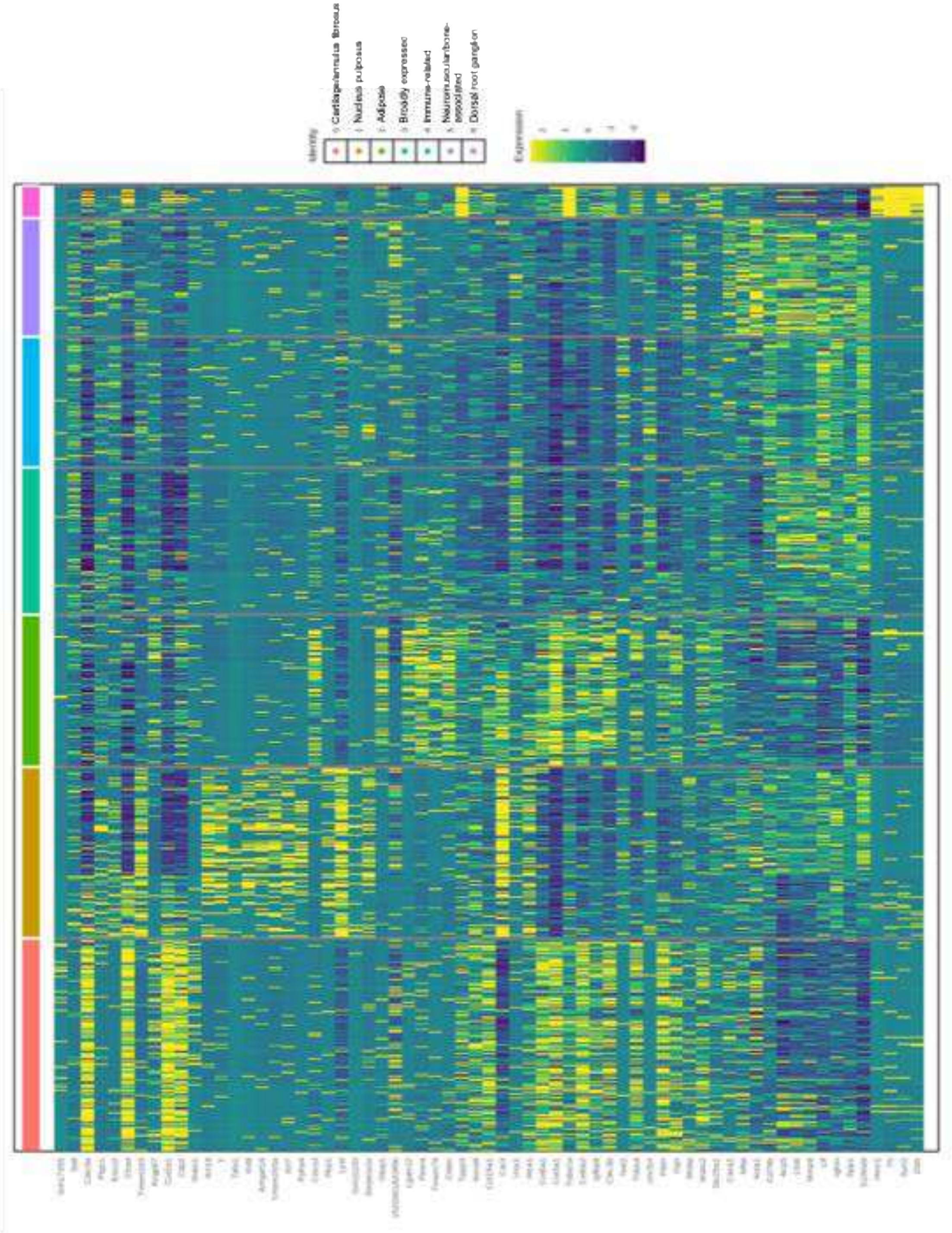

Supplemental Figure 2. IVD specific gene cluster analysis and heatmap. Rows indicate the unique set of the top 15 expressed genes per cluster. Columns show cells grouped by clusters (color bar above). Values depict scaled normalized expression.

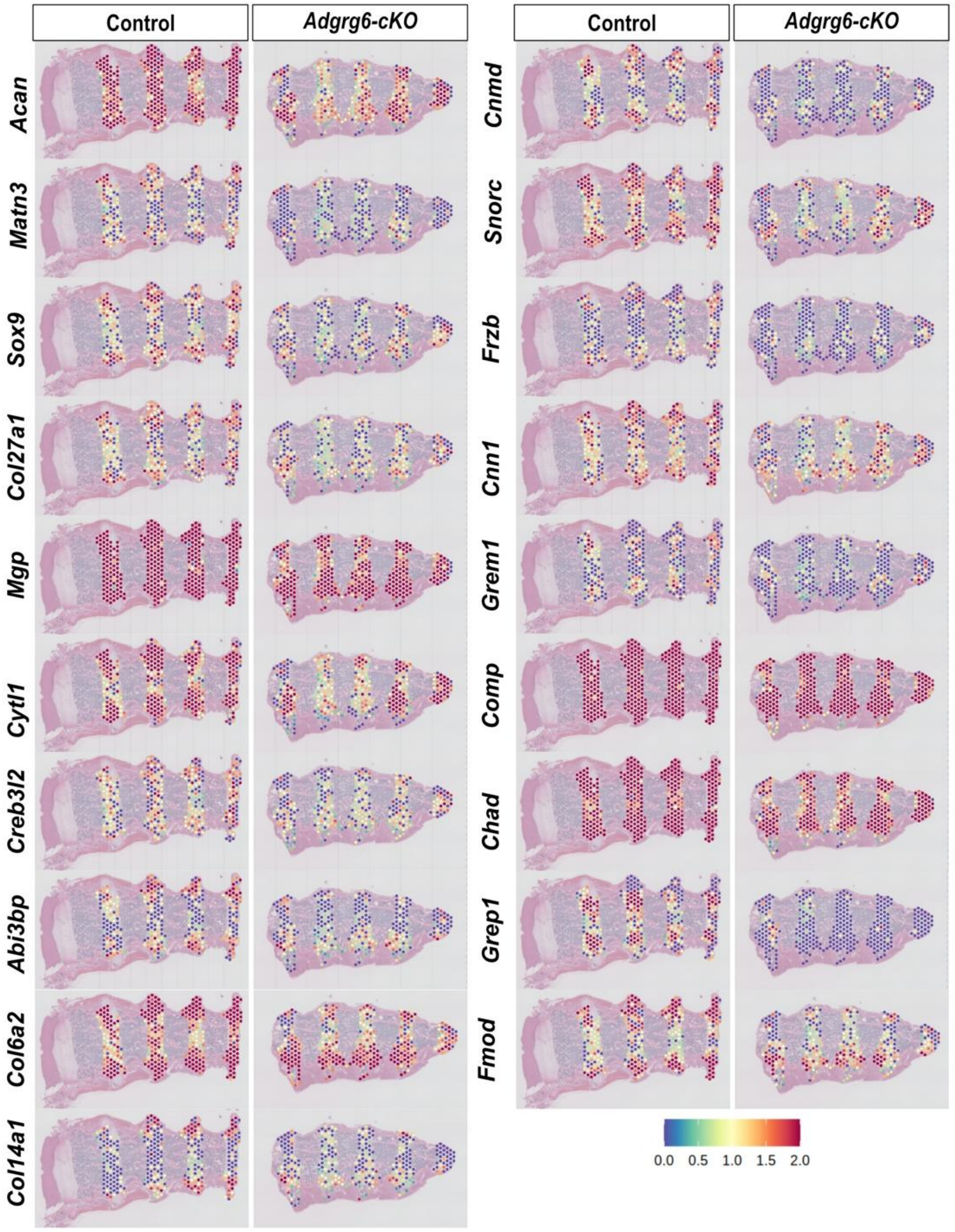

Supplemental Figure 3. Intervertebral disc specific spatial gene expression altered in *Adgrg6-cKO* mice. Spatial feature plots show log-normalized cartilage development and extracellular matrix organization transcript abundance per spot. Color intensity corresponds to expression magnitude, with consistent scale across genes and samples.

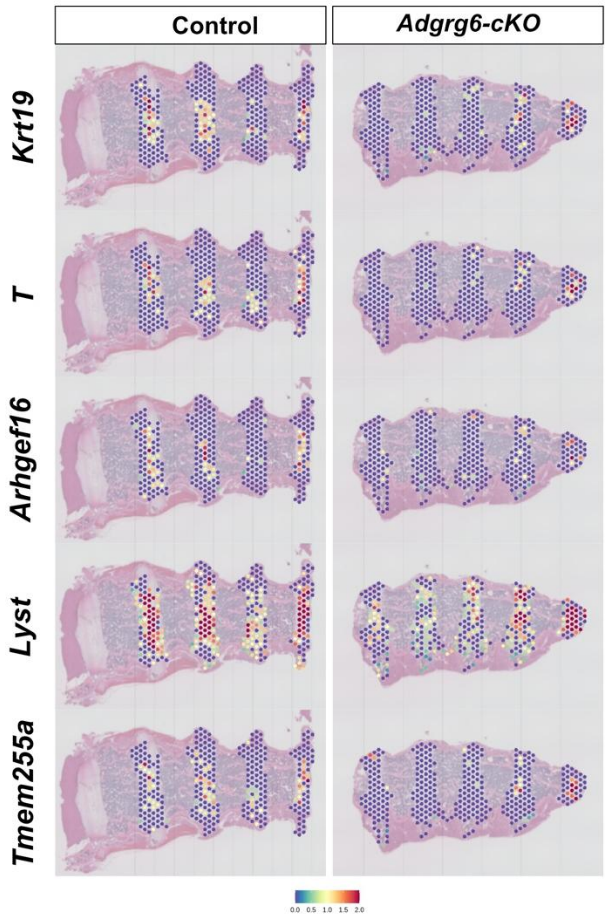

Supplemental Figure 4. Nucleus pulposus specific spatial gene expression in wildtype and *Adgrg6-cKO* mice. Spatial feature plots show log-normalized nucleus pulposus transcript abundance per spot. Color intensity corresponds to expression magnitude, with consistent scale across genes and samples.

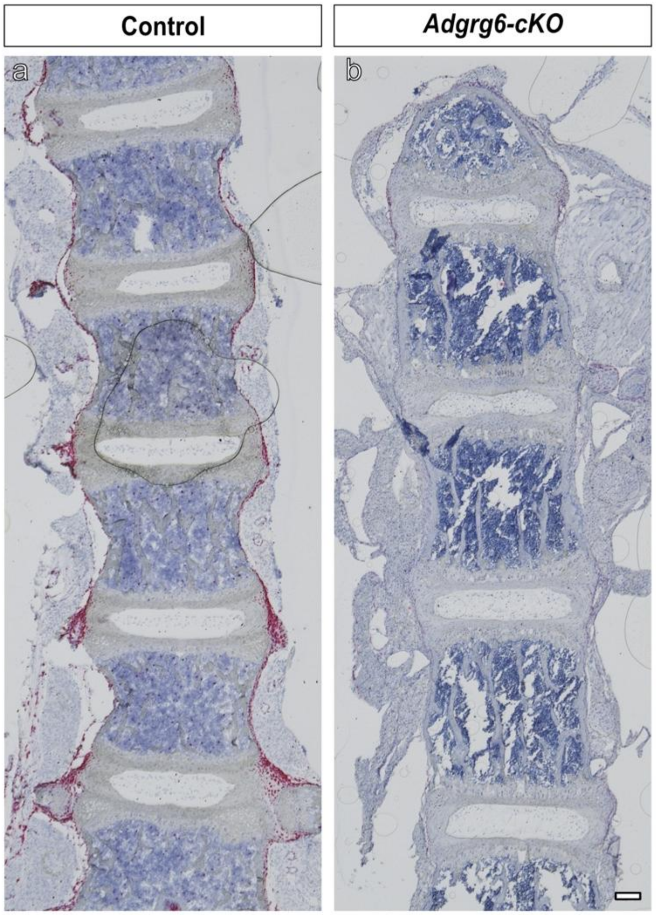

Supplemental Figure 5. *Col14a1* is expressed in periosteum-like tissue adjacent to the spine. RNAScope *in situ* hybridization was performed to detect *Col14a1* expression in sagittal sections of the thoracic spine. In control mice, robust *Col14a1* expression is observed in periosteum-like tissue surrounding the vertebral column (a). In *Adgrg6-cKO* spines, *Col14a1* expression is markedly reduced and largely absent within these paraspinal tissues (b). Scale bar = 100 μm.

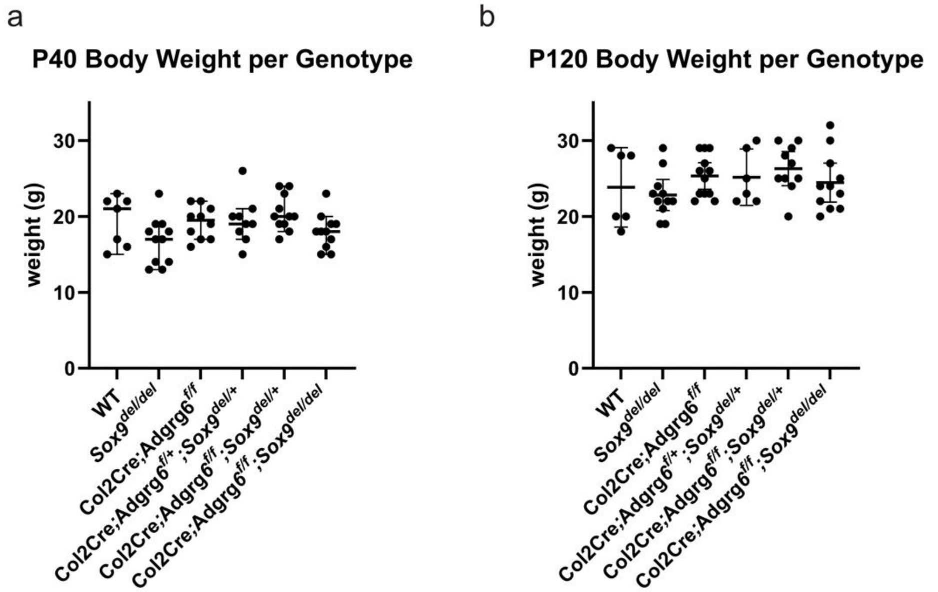

Supplemental Figure 6. Longitudinal body weight measurements indicate normal growth patterns in experimental and control mice. Body weight measures of mutant and control mice scanned at P40 (a) and P120 (b) are not significantly different. Individual points are shown, with median ± 95% confidence intervals overlaid.

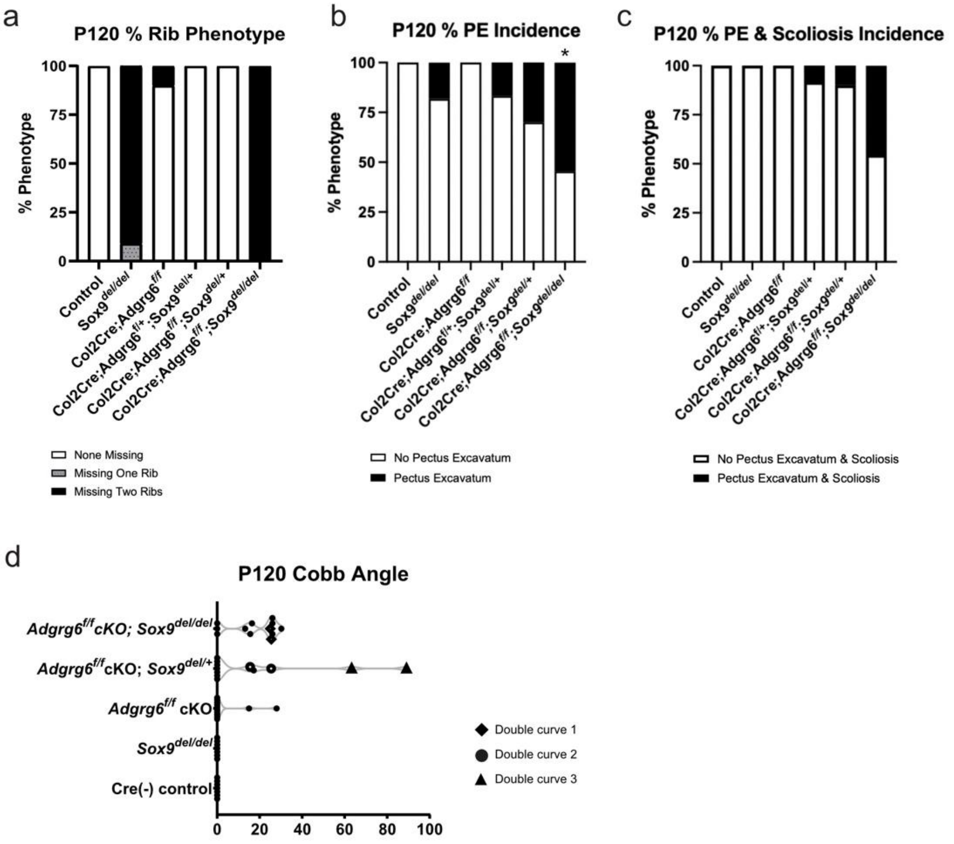

Supplemental Figure 7. Genotype-dependent incidence of scoliosis, T13 rib loss, and pectus excavatum phenotypes at P120. Histogram of the percentage of mice exhibiting loss of the T13 rib pair phenotype at P120 across genotypes (a). Histogram of the incidence of pectus excavatum (PE) across genotypes (b). Fisher's Exact Test for pairwise comparisons of PE incidence count at P120 showed significant genotype-dependent differences in *Adgrg6-cKO*; *Sox9*$^{del/del}$ double mutant mice when compared across genotype, * = $p \leq 0.05$ (b). Histogram of the concurrent of PE and scoliosis at P120 (c), statistical analysis using Fisher's Exact Test for pairwise comparisons showed no significant genotype-dependent differences for this relationship. Scoliosis severity distributions differed significantly by genotype, with *Adgrg6*-cKO; *Sox9*$^{del/del}$ double mutant mice significantly different from *Sox9*^{del}, Col2Cre; *Adgrg6*$^{f/+}$; *Sox9*$^{del/+}$, and control mice ($p \leq 0.01$)(d). Increased incidence and severity of thoracic scoliosis in *Adgrg6*-cKO; *Sox9*$^{del/+}$ and *Adgrg6*-cKO; *Sox9*$^{del/del}$ mutant mice quantification shows higher incidence of thoracic scoliosis and increased severity compared with controls (d).

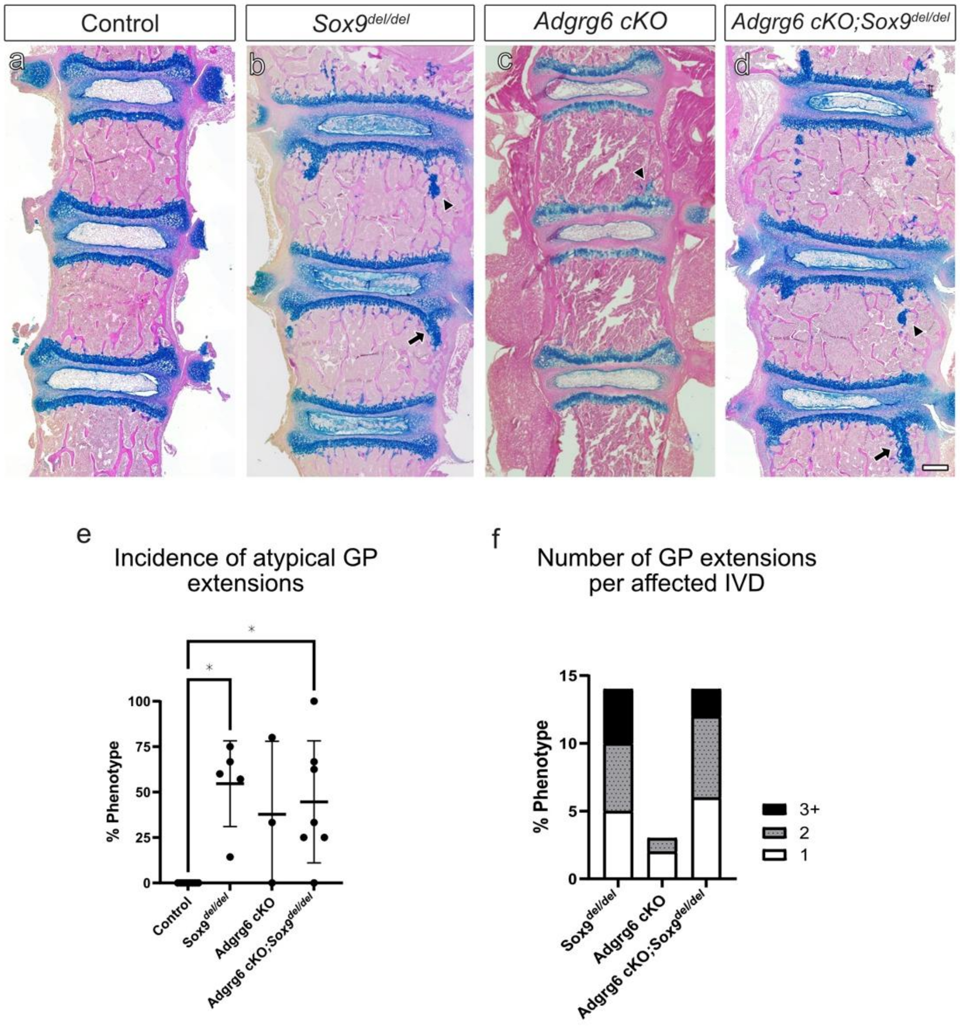

Supplemental Figure 8. Histological analysis at (P)20 reveals growth plate defects and ectopic cartilage regions in vertebral bone of *Adgrg6*-cKO; *Sox9*$^{del/del}$ double mutant mice. Alcian Blue Hematoxylin Eosin Orange G stain reveals growth plate (GP) extensions (arrows) and ectopic cartilaginous regions in the vertebrae that are disconnected from the GP (arrowheads) in *Sox9*^{del}, *Adgrg6*-cKO, and *Adgrg6*-cKO; *Sox9*$^{del/del}$ double mutant mice (b-d), that are not observed in wild-type mice (a). The incidence of these growth plate defects was quantified as the percentage of affected IVDs per spine, with each point representing an individual mouse with the median ± 95% confidence intervals overlaid (e). One way ANOVA identified genotype as a major source of variation (38% of total variance), with *Sox9*^{del} and *Adgrg6*-cKO; *Sox9*$^{del/del}$ exhibiting significantly increased incidence compared to controls ($p \leq 0.05$)(e). *Sox9*^{del} and *Adgrg6*-cKO; *Sox9*$^{del/del}$ double mutant mice exhibited increased severity of GP defects, exhibit higher incidence of three or more defects per affected IVD (f). Scale bar = 500 um.

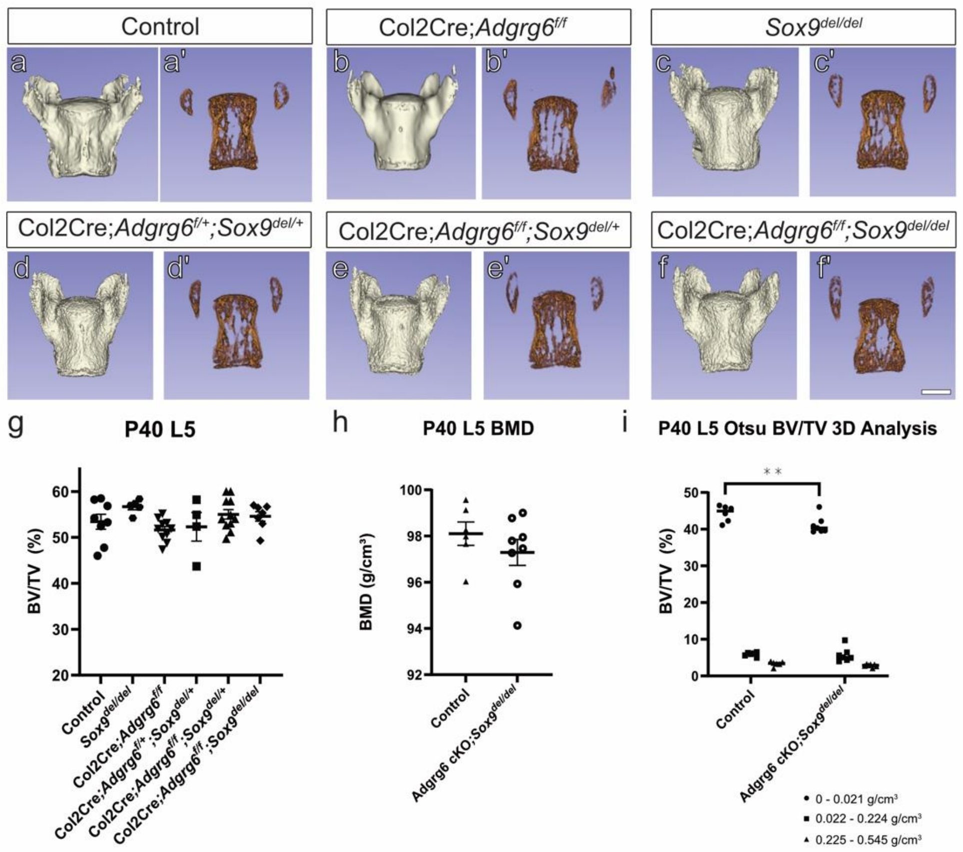

Supplemental Figure 9. Lumbar (L)5 vertebral microarchitecture is largely preserved across genotypes at P40. MicroCT reconstructions of lumbar vertebrae (L)5 reveal no gross morphological differences across genotypes (a-f). Representative 10 μm midline slices similarly show no gross alterations of inner bone among genotypes (a'-f'). One way ANOVA and t-test analysis of bone microarchitecture revealed no significant changes in total bone volume (g) or bone mineral density (h). Two way ANOVA of OTSU-based threshold 3D bone volume analysis stratifying bone into low- (0–0.021 g/cm³), mid- (0.022 – 0.224 g/cm³), and high-density (0.225–0.545 g/cm³) fractions reveals a significant loss of low-density distribution in double mutant mice ($p < 0.05$) (i). Each dot represents an individual mouse, with mean ± SEM overlaid. Scale bar = 1 mm.

Supporting Documents

Table 1. Unsupervised clustering analysis of spatial analysis over intervertebral disc regions. Markers were identified for each cluster using the SCT-normalized data. Values in the Log Fold Change column represent the natural log fold change (log-normalized) of gene expression in the target cluster relative to all other spots. The pct.1 and pct.2 columns indicate the proportion of spots expressing the gene within the target cluster and across all other clusters, respectively. Marker genes shown were restricted to those with a minimum detection frequency of 25% within the cluster to ensure biological representativeness. Statistical significance was defined as a Bonferroni-adjusted p-value < 0.05.

Table 2. Differential gene expression of refined spatial analysis over intervertebral disc regions excluding high-muscle expressing capture spots. Differential expression was performed using the non-parametric Wilcoxon Rank Sum test. For condition-specific comparisons (*Adgrg6*-cKO vs. wildtype), data were subset to remove capture spots enriched for muscle-related transcripts and normalized using standard log-normalization. Statistical significance was defined as a Bonferroni-adjusted p-value < 0.05. Only genes detected in at least 10% of spots in either comparison group with a minimum natural log fold change of 0.25 were included.

Table 3. Longitudinal Cobb angle measurements in mice presenting with thoracic scoliosis. Cobb angles were quantified for all experimental genotypes in the dataset that had scoliosis that exhibited a measurable thoracic curvature (>10 degrees). “No curve” denotes a curve less than 10 degrees or the absence of noticeable scoliosis. At P40, *Col2Cre;Adgrg6*$^{f/f}$*;Sox9*$^{del/del}$ presented with five single curves and three *no curves*. By P120, previously straight spines developed a single curve, resulting in a total of seven single curves and one double curve. Double thoracic curvatures are denoted by two Cobb angles (e.g. 45, 77).

Table 1

Gene	Log2 Fold Change	pct.1	pct.2	Adjusted *p*-value	Cluster
**Gm17455**	2.5519	0.101	0.017	7.2633E-03	0
**Clec3a**	2.2246	1.000	0.861	5.8148E-54	0
**Chad**	2.0002	1.000	0.945	6.9435E-56	0
**Col2a1**	1.9826	1.000	1.000	8.8644E-61	0
**Cnmd**	1.9808	0.766	0.356	7.5935E-27	0
**Cilp2**	1.9743	0.994	0.734	2.6639E-45	0
**Angptl7**	1.9712	0.329	0.152	1.8340E-03	0
**Matn1**	1.9468	0.196	0.059	1.2984E-03	0
**Abi3bp**	1.9276	0.842	0.417	1.1311E-31	0
**Comp**	1.8836	1.000	0.972	2.0311E-63	0
**Nfatc2**	1.8500	0.525	0.206	1.4051E-13	0
**Chst1**	1.8491	0.215	0.075	4.7851E-03	0
**Clec3b**	1.8121	0.835	0.556	1.9988E-16	0
**Thbs4**	1.7571	0.797	0.479	8.3121E-19	0
**Fibin**	1.7516	0.943	0.650	1.6625E-32	0
**Extl1**	1.7110	0.278	0.092	1.6587E-05	0
**Cytl1**	1.6999	0.981	0.785	2.5593E-31	0
**Shisa2**	1.6989	0.184	0.061	2.4254E-02	0
**Scrg1**	1.6794	0.652	0.272	6.7688E-18	0
**Fmod**	1.6747	0.956	0.690	1.4182E-30	0
**Fosb**	1.6635	0.222	0.084	2.4378E-02	0
**Col9a2**	1.6552	0.956	0.701	2.7405E-31	0
**Ecm2**	1.6327	0.399	0.150	6.1896E-08	0
**Pax1**	1.5913	0.342	0.125	2.6244E-06	0
**Col14a1**	1.5729	0.658	0.413	7.7362E-09	0
**Prelp**	1.5600	1.000	0.883	9.0978E-36	0
**Chadl**	1.5348	0.506	0.261	2.1630E-07	0
**Emilin3**	1.5233	0.272	0.110	4.2316E-03	0
**Calml3**	1.5019	0.348	0.147	6.6917E-05	0
**Trpv4**	1.4828	0.551	0.281	2.3069E-09	0
**Col15a1**	1.4748	0.880	0.613	1.3172E-23	0
**Grem1**	1.4690	0.513	0.295	3.6272E-05	0
**Matn2**	1.4617	0.652	0.328	3.4212E-12	0
**Mn1**	1.4588	0.487	0.266	2.2877E-05	0
**Setbp1**	1.4538	0.481	0.253	6.4350E-06	0
**Stc2**	1.4500	0.342	0.167	6.0682E-03	0
**Col6a2**	1.4302	1.000	0.905	6.8586E-41	0
**Col9a3**	1.4213	0.994	0.835	7.2642E-32	0
**Loxl3**	1.4185	0.835	0.464	8.2116E-21	0
**Gas1**	1.4071	1.000	0.886	2.0951E-28	0
**Nt5e**	1.4010	0.399	0.209	8.8431E-04	0
**Clu**	1.3998	0.766	0.528	1.7934E-15	0
**Col6a1**	1.3984	0.994	0.884	8.3535E-36	0

Table 1

**Col9a1**	1.3948	1.000	0.974	2.4127E-48	0
**Col8a1**	1.3933	0.386	0.200	1.8472E-03	0
**Cilp**	1.3927	0.418	0.209	1.9982E-04	0
**Col16a1**	1.3909	0.994	0.901	5.5827E-30	0
**Sox9**	1.3634	0.867	0.549	5.6331E-21	0
**Loxl2**	1.3618	0.734	0.457	5.9155E-13	0
**Pamr1**	1.3609	0.551	0.352	1.8850E-04	0
**Wif1**	1.3556	0.911	0.681	8.8827E-21	0
**Sertad4**	1.3389	0.361	0.169	9.8242E-04	0
**Pkd2**	1.3349	0.741	0.429	1.3469E-12	0
**Igfbp6**	1.3269	0.462	0.215	5.2374E-06	0
**Plod2**	1.3144	0.671	0.457	4.1572E-09	0
**Col3a1**	1.3142	1.000	0.982	1.3904E-25	0
**Thbs3**	1.2810	0.741	0.477	1.5797E-10	0
**Mfge8**	1.2701	0.595	0.339	4.7767E-08	0
**Snorc**	1.2582	0.892	0.657	7.3291E-20	0
**Frzb**	1.2544	0.494	0.316	3.2766E-03	0
**Col6a3**	1.2516	1.000	0.972	2.6581E-40	0
**Matn3**	1.2505	0.665	0.415	4.4817E-08	0
**Thbs2**	1.2387	0.810	0.580	4.6650E-11	0
**Ogn**	1.2267	0.658	0.426	4.2220E-07	0
**Bgn**	1.2218	1.000	0.980	6.8086E-39	0
**Dlk1**	1.2100	0.557	0.376	1.3727E-03	0
**Col27a1**	1.2064	0.873	0.613	6.3085E-18	0
**Igfbp7**	1.1974	1.000	0.972	2.8745E-28	0
**Pdgfrl**	1.1938	0.494	0.273	5.4729E-05	0
**Hapln1**	1.1851	0.854	0.624	1.6273E-15	0
**B4galnt3**	1.1742	0.671	0.411	1.6572E-08	0
**Dkk3**	1.1694	0.608	0.383	4.0965E-06	0
**Dpt**	1.1656	0.386	0.200	6.1413E-03	0
**Anxa8**	1.1319	0.614	0.339	1.1003E-07	0
**Trps1**	1.1128	0.614	0.394	2.4062E-06	0
**Meltf**	1.1013	0.595	0.343	2.6148E-06	0
**Hspb7**	1.0781	0.437	0.259	3.7917E-02	0
**Plagl1**	1.0664	0.582	0.378	5.2099E-04	0
**Fbln7**	1.0590	0.525	0.330	1.2614E-02	0
**Col5a1**	1.0537	1.000	0.998	4.0254E-28	0
**Fhl1**	1.0445	0.646	0.448	9.7585E-05	0
**Dcn**	1.0389	0.899	0.732	2.5413E-10	0
**Mgp**	1.0280	1.000	0.987	1.4122E-28	0
**Islr**	1.0227	0.924	0.752	8.8803E-14	0
**Chst11**	1.0169	0.690	0.461	3.8192E-06	0
**Fn1**	1.0041	1.000	0.980	1.8844E-26	0
**Angptl2**	1.0022	0.816	0.604	1.6963E-08	0

Table 1

**Dnm1**	0.9892	0.835	0.613	5.2763E-11	0
**Cspg4**	0.9878	0.816	0.624	9.6302E-11	0
**Trim47**	0.9763	0.690	0.475	9.4812E-06	0
**Tgfbi**	0.9659	0.563	0.411	2.9962E-02	0
**Nr1d1**	0.9637	0.696	0.481	2.5188E-06	0
**Myh1**	0.9401	0.715	0.560	1.7410E-02	0
**Pam**	0.9327	0.589	0.385	1.0198E-03	0
**Cdkn1c**	0.9286	0.797	0.659	1.2909E-04	0
**Sod3**	0.9003	0.797	0.620	3.3835E-06	0
**Tnnt3**	0.8687	0.785	0.650	5.9230E-03	0
**Thbs1**	0.8631	0.987	0.912	6.0040E-18	0
**Myh2**	0.8543	0.608	0.400	8.2092E-03	0
**Mxra7**	0.8479	0.753	0.600	1.9623E-04	0
**Smoc2**	0.8191	0.804	0.594	7.7490E-08	0
**Sdc4**	0.8114	0.987	0.961	3.3072E-20	0
**Acan**	0.8096	0.994	0.892	1.6413E-18	0
**Rarg**	0.7969	0.785	0.602	2.7251E-05	0
**Ckm**	0.7947	0.747	0.604	4.9077E-03	0
**Fgfrl1**	0.7930	0.690	0.512	4.4113E-03	0
**Gsn**	0.7923	0.962	0.921	1.2999E-11	0
**Htra3**	0.7333	0.753	0.629	2.2622E-02	0
**Pcsk6**	0.7324	0.658	0.508	4.8458E-02	0
**Marveld1**	0.7299	0.804	0.596	1.5771E-05	0
**Wwp2**	0.7272	0.848	0.769	1.3549E-05	0
**Sparc**	0.7047	1.000	1.000	1.7952E-46	0
**Ndufa4l2**	0.7039	0.696	0.483	3.4844E-04	0
**Eln**	0.6835	0.987	0.919	8.1657E-06	0
**Ptgis**	0.6654	0.930	0.800	7.0845E-07	0
**Plec**	0.6614	0.968	0.848	1.6443E-06	0
**Phldb1**	0.6598	0.930	0.782	2.0192E-07	0
**Col5a2**	0.6573	1.000	0.996	6.9999E-15	0
**Ccn1**	0.6566	0.861	0.769	7.3204E-04	0
**Timp2**	0.6441	0.899	0.862	3.2605E-04	0
**Ski**	0.6420	0.962	0.916	2.8105E-07	0
**Susd5**	0.6160	0.873	0.668	2.4975E-07	0
**Cst3**	0.5995	1.000	1.000	1.0160E-18	0
**Itm2c**	0.5558	0.930	0.828	1.6139E-04	0
**Serpinh1**	0.5481	1.000	1.000	1.2961E-16	0
**Timp3**	0.5131	0.975	0.875	1.2909E-04	0
**Krt19**	4.6891	0.548	0.049	5.6755E-47	1
**T**	4.4016	0.452	0.043	8.8841E-36	1
**Tafa1**	4.0696	0.143	0.009	2.8486E-10	1
**Krt8**	3.7801	0.302	0.028	1.4205E-21	1
**Arhgef16**	3.6633	0.357	0.047	3.9745E-22	1

Table 1

**Tmem255a**	3.5380	0.421	0.061	2.0986E-25	1
**Krt7**	3.4409	0.373	0.052	4.3140E-22	1
**Ppfia4**	3.4175	0.302	0.035	5.5872E-19	1
**Lyst**	3.2885	0.913	0.402	2.5847E-45	1
**Gm11100**	3.2593	0.127	0.016	1.5610E-05	1
**Serpina1e**	3.1620	0.397	0.066	6.2302E-21	1
**Pkp1**	3.1521	0.238	0.028	8.0914E-14	1
**2200002D01Rik**	3.0316	0.159	0.021	2.7176E-07	1
**Dmrta1**	3.0025	0.127	0.019	1.6701E-04	1
**Sostdc1**	2.9414	0.381	0.090	1.4466E-14	1
**Tmem163**	2.9231	0.659	0.179	1.7717E-30	1
**Adcy5**	2.9114	0.421	0.085	2.4485E-19	1
**Gldn**	2.8326	0.103	0.014	1.7016E-03	1
**Lamb3**	2.7097	0.214	0.033	1.5345E-09	1
**Frmd5**	2.7097	0.127	0.021	5.5326E-04	1
**Dsc2**	2.5362	0.127	0.024	3.7964E-03	1
**Jph2**	2.4118	0.651	0.239	1.8219E-21	1
**Rab38**	2.3472	0.317	0.083	2.5065E-09	1
**Car3**	2.2814	1.000	0.995	7.0700E-32	1
**Slc12a2**	2.2761	0.294	0.075	2.9474E-08	1
**Pde1a**	2.2602	0.317	0.104	1.8173E-06	1
**Cdh6**	2.1280	0.151	0.033	2.1970E-03	1
**Fxyd6**	2.1250	0.635	0.296	3.0395E-15	1
**Zim1**	1.9981	0.246	0.071	6.0003E-05	1
**Slc2a1**	1.9217	0.651	0.380	5.5831E-12	1
**Hopx**	1.8166	0.325	0.140	1.1264E-03	1
**Lgals3**	1.6334	0.952	0.828	1.8297E-21	1
**Gpc3**	1.5365	0.516	0.321	6.5809E-03	1
**Ppp1r3c**	1.4801	0.333	0.137	9.6187E-04	1
**Susd5**	1.3774	0.929	0.667	5.1441E-16	1
**Ndufa4l2**	1.3334	0.706	0.492	6.6748E-08	1
**Dst**	1.2540	0.579	0.354	7.6065E-04	1
**Csrp3**	1.2273	0.381	0.180	6.0044E-03	1
**Htra1**	1.1686	0.841	0.735	9.6920E-05	1
**Syne1**	1.1320	0.532	0.341	3.5893E-02	1
**Vim**	1.0852	1.000	0.998	2.3868E-12	1
**App**	1.0758	1.000	0.997	1.2080E-15	1
**Ttn**	0.9728	0.484	0.274	2.5784E-02	1
**Acta2**	0.9691	0.587	0.402	3.9701E-02	1
**Acan**	0.9687	1.000	0.896	6.7883E-11	1
**Tcap**	0.9289	0.532	0.300	1.7846E-03	1
**Smim1**	0.8055	0.698	0.503	4.6072E-02	1
**Cryab**	0.7739	0.683	0.515	1.3104E-02	1
**Nap1l1**	0.6817	0.984	0.931	1.7177E-07	1

Table 1

**Myh1**	0.5756	0.730	0.565	2.9728E-02	1
**Tpm1**	0.5680	0.992	0.971	3.7857E-05	1
**Irs3**	3.0780	0.162	0.019	5.3343E-08	2
**Nppc**	3.0589	0.207	0.017	1.0355E-13	2
**Mpzl2**	3.0525	0.225	0.029	1.6930E-11	2
**Gstm7**	3.0431	0.144	0.017	1.2747E-06	2
**Ttc36**	2.9556	0.135	0.017	1.2038E-05	2
**Lgals12**	2.8625	0.423	0.062	7.3037E-23	2
**Adhfe1**	2.8480	0.225	0.032	2.1223E-10	2
**Pm20d1**	2.8301	0.153	0.024	1.6984E-05	2
**Ppara**	2.7978	0.739	0.194	2.3207E-34	2
**Lurap1**	2.7935	0.108	0.015	2.3476E-03	2
**Eda**	2.7935	0.108	0.015	2.3476E-03	2
**Tfb2m**	2.7561	0.162	0.024	2.3765E-06	2
**Tmem79**	2.7370	0.369	0.064	7.2609E-17	2
**Cidec**	2.7232	0.658	0.145	1.0862E-31	2
**Apoo**	2.7199	0.162	0.027	1.7700E-05	2
**Magix**	2.7199	0.144	0.027	8.7553E-04	2
**Kcnk3**	2.6753	0.910	0.301	6.4254E-43	2
**Otop1**	2.6749	0.505	0.108	2.1747E-21	2
**Slc22a3**	2.6495	0.279	0.051	4.7105E-11	2
**Echdc3**	2.6077	0.342	0.062	2.2734E-14	2
**Ebf2**	2.5884	0.369	0.069	1.5725E-15	2
**Pparg**	2.5792	0.550	0.111	1.0830E-24	2
**Cox7a1**	2.5694	0.982	0.584	7.9725E-45	2
**Angptl8**	2.5670	0.162	0.027	2.0476E-05	2
**Ces1d**	2.5479	0.640	0.154	4.5234E-27	2
**Il20rb**	2.5305	0.108	0.019	2.0265E-02	2
**Mfsd4b1**	2.5305	0.108	0.019	2.0265E-02	2
**Inca1**	2.5081	0.135	0.024	1.0631E-03	2
**Hoxa7**	2.5025	0.144	0.025	3.9468E-04	2
**Egflam**	2.5025	0.144	0.025	3.9468E-04	2
**Gpd1**	2.4992	1.000	0.730	8.8100E-45	2
**Tmem37**	2.4975	0.144	0.027	9.3195E-04	2
**Adig**	2.4962	0.919	0.421	1.6137E-34	2
**Npr3**	2.4952	0.450	0.096	3.8040E-18	2
**Adrb3**	2.4878	0.378	0.084	1.8094E-13	2
**Klf15**	2.4854	0.532	0.122	1.4549E-21	2
**Pck1**	2.4727	0.973	0.586	2.5912E-43	2
**Papln**	2.4608	0.252	0.047	4.1289E-09	2
**Pld6**	2.4581	0.261	0.047	5.9581E-10	2
**Pdk4**	2.4248	0.658	0.174	1.6767E-25	2
**Cfd**	2.4235	0.991	0.706	2.5691E-43	2
**Etfrf1**	2.4150	0.441	0.093	1.4450E-17	2

Table 1

**Acaa1b**	2.4150	0.306	0.051	2.6806E-13	2
**Cyp2u1**	2.4150	0.306	0.061	3.1270E-11	2
**Adtrp**	2.4150	0.270	0.049	2.5467E-10	2
**Adra1a**	2.4150	0.207	0.035	1.4740E-07	2
**Sgk2**	2.4150	0.189	0.034	2.8333E-06	2
**Flacc1**	2.4150	0.162	0.032	2.9686E-04	2
**Lipt1**	2.4150	0.135	0.025	2.6790E-03	2
**Apoc1**	2.4085	0.964	0.549	5.3833E-39	2
**Trarg1**	2.4005	0.523	0.142	1.5960E-17	2
**Aoc3**	2.3976	0.766	0.216	1.7892E-31	2
**Ppp1r3b**	2.3954	0.514	0.105	5.1838E-22	2
**Thrsp**	2.3919	0.946	0.584	2.0019E-37	2
**Cidea**	2.3826	1.000	0.655	4.0195E-44	2
**Pbld1**	2.3811	0.324	0.064	3.9916E-12	2
**Klhdc7a**	2.3768	0.676	0.201	1.0337E-23	2
**Ucp1**	2.3747	0.991	0.713	6.6860E-37	2
**Etl4**	2.3692	0.252	0.051	2.2690E-08	2
**Oxnad1**	2.3685	0.405	0.093	2.3666E-14	2
**Plin1**	2.3598	0.919	0.554	1.1369E-35	2
**Rasl12**	2.3536	0.477	0.105	1.0727E-18	2
**Ppargc1a**	2.3463	0.306	0.066	3.3376E-10	2
**Plin5**	2.3446	0.414	0.088	8.6349E-16	2
**Ablim3**	2.3370	0.432	0.108	2.8654E-14	2
**Slc25a42**	2.3343	0.712	0.198	2.2893E-28	2
**Ajuba**	2.3326	0.135	0.029	1.3321E-02	2
**Ntrk3**	2.3301	0.694	0.184	8.7954E-27	2
**Mrap**	2.3301	0.423	0.101	1.3982E-14	2
**A530016L24Rik**	2.3301	0.270	0.049	3.2840E-10	2
**Adh1**	2.3276	0.369	0.076	7.5073E-14	2
**Mcrip2**	2.3231	0.793	0.279	8.7128E-29	2
**Acsm3**	2.3214	0.757	0.228	1.4573E-28	2
**Blcap**	2.3155	0.117	0.024	4.5614E-02	2
**Esrrg**	2.3119	0.216	0.042	7.8365E-07	2
**Fabp4**	2.3051	1.000	0.978	2.2475E-47	2
**Itpka**	2.2996	0.297	0.059	1.3952E-10	2
**Cpt1b**	2.2953	0.883	0.346	5.8074E-33	2
**Cox8b**	2.2910	1.000	0.932	1.9091E-46	2
**Tmem45b**	2.2895	0.667	0.199	2.4108E-23	2
**Pex11a**	2.2895	0.261	0.049	2.3054E-09	2
**Gsta4**	2.2873	0.405	0.093	4.8730E-14	2
**Dgat2**	2.2828	0.982	0.642	2.8710E-39	2
**Phyh**	2.2730	0.387	0.100	1.5883E-11	2
**Aqp7**	2.2724	0.739	0.203	4.6320E-28	2
**Clstn3**	2.2706	0.604	0.166	2.1502E-21	2

Table 1

**Gpd2**	2.2666	0.775	0.282	1.2657E-25	2
**Rnf152**	2.2603	0.405	0.090	1.3694E-14	2
**Nectin2**	2.2558	0.396	0.093	3.3010E-13	2
**Jag2**	2.2451	0.189	0.044	3.0681E-04	2
**Pnpla2**	2.2427	1.000	0.875	2.0930E-46	2
**Nudt12**	2.2400	0.243	0.057	2.5066E-06	2
**Ldhd**	2.2316	0.387	0.098	1.1028E-11	2
**Aifm2**	2.2306	0.649	0.218	8.2689E-20	2
**Slc25a20**	2.2257	0.874	0.355	3.8968E-32	2
**Slc24a3**	2.2249	0.468	0.117	4.4663E-16	2
**Slc25a34**	2.2224	0.207	0.049	6.7691E-05	2
**Ces1f**	2.2224	0.171	0.039	1.1128E-03	2
**Mlxipl**	2.2165	0.432	0.101	7.5398E-15	2
**Slc4a4**	2.2161	0.586	0.169	6.5295E-19	2
**Tppp**	2.2147	0.333	0.083	1.1881E-09	2
**Ddo**	2.2134	0.297	0.068	4.3353E-09	2
**Slc25a35**	2.2134	0.306	0.073	5.2510E-09	2
**Cyp2e1**	2.2134	0.171	0.034	1.3527E-04	2
**Aco2**	2.2109	0.991	0.720	1.4761E-39	2
**Cadm4**	2.2009	0.162	0.044	3.1606E-02	2
**P2rx5**	2.1987	0.414	0.117	1.1960E-11	2
**Acsl1**	2.1969	0.486	0.157	5.1197E-13	2
**Zic1**	2.1969	0.279	0.088	5.5896E-05	2
**Agpat2**	2.1964	0.622	0.184	9.2500E-21	2
**Adcy9**	2.1926	0.279	0.066	9.5069E-08	2
**Prss36**	2.1926	0.153	0.034	4.1996E-03	2
**Scara5**	2.1806	0.477	0.125	2.6073E-15	2
**Rilp**	2.1740	0.189	0.039	4.1293E-05	2
**Adm**	2.1740	0.189	0.042	1.6363E-04	2
**Ucp3**	2.1740	0.171	0.037	6.0683E-04	2
**Acacb**	2.1665	0.946	0.542	4.9052E-32	2
**Pank1**	2.1659	0.622	0.182	1.9479E-20	2
**Adrb1**	2.1613	0.342	0.093	5.3459E-09	2
**Angptl4**	2.1601	0.604	0.194	1.0260E-17	2
**1190005I06Rik**	2.1563	0.369	0.103	8.1989E-10	2
**Fam13a**	2.1562	0.766	0.252	7.5576E-28	2
**Coq6**	2.1552	0.468	0.135	2.7358E-13	2
**F3**	2.1520	0.279	0.069	2.9378E-07	2
**Acaa2**	2.1508	1.000	0.821	2.1904E-42	2
**Coq8a**	2.1502	0.991	0.711	3.4100E-39	2
**Siah1a**	2.1420	0.198	0.041	1.5061E-05	2
**Acadvl**	2.1403	1.000	0.718	1.3304E-41	2
**Adora1**	2.1394	0.162	0.034	8.2479E-04	2
**Cib2**	2.1391	0.730	0.267	5.6224E-22	2

Table 1

**Lipe**	2.1372	0.991	0.652	1.9984E-40	2
**Cpt2**	2.1367	0.667	0.228	1.0061E-19	2
**Fbxo21**	2.1326	0.288	0.069	5.6841E-08	2
**Fabp3**	2.1291	0.910	0.556	2.2265E-32	2
**Rasd1**	2.1268	0.577	0.179	2.0120E-17	2
**Retsat**	2.1265	0.703	0.209	1.3367E-23	2
**Nr2f2**	2.1255	0.216	0.047	8.4592E-06	2
**Ostn**	2.1255	0.189	0.051	3.7424E-03	2
**Nos2**	2.1255	0.144	0.034	2.2700E-02	2
**Zfp366**	2.1255	0.144	0.035	4.2041E-02	2
**Akap1**	2.1244	0.604	0.182	8.8607E-19	2
**Ehhadh**	2.1236	0.505	0.117	3.6909E-18	2
**Pdhb**	2.1155	0.495	0.122	6.2154E-17	2
**Aspg**	2.1139	0.360	0.098	2.0425E-09	2
**Ybx2**	2.1125	0.225	0.047	1.9836E-06	2
**Eci1**	2.1083	1.000	0.794	5.7227E-43	2
**Timp4**	2.1061	0.459	0.122	5.0453E-14	2
**Ephx2**	2.1057	0.595	0.150	6.8084E-21	2
**Nfib**	2.1019	0.604	0.167	1.2066E-20	2
**Cbr4**	2.0931	0.234	0.057	1.5218E-05	2
**Adam33**	2.0931	0.198	0.046	1.3129E-04	2
**Abhd15**	2.0825	0.514	0.149	9.9073E-15	2
**Arhgap29**	2.0794	0.333	0.083	2.0234E-09	2
**Sucla2**	2.0790	0.964	0.562	5.9226E-37	2
**Acad10**	2.0780	0.153	0.037	1.6850E-02	2
**Acadm**	2.0766	0.982	0.721	1.0410E-37	2
**Oplah**	2.0731	0.532	0.147	2.2725E-16	2
**Sox17**	2.0721	0.315	0.081	2.7074E-08	2
**Cisd3**	2.0711	0.667	0.220	2.6499E-21	2
**Rxrg**	2.0711	0.369	0.095	1.4855E-10	2
**Clic5**	2.0651	0.586	0.186	3.0070E-17	2
**Gstm2**	2.0636	0.234	0.057	1.6265E-05	2
**Lpl**	2.0619	0.973	0.738	5.4994E-36	2
**Chp2**	2.0614	0.153	0.035	9.1657E-03	2
**Hadh**	2.0580	0.874	0.454	8.1938E-26	2
**Acer2**	2.0575	0.270	0.062	1.7301E-07	2
**Ndufa6**	2.0560	0.586	0.167	2.8412E-18	2
**Chdh**	2.0525	0.279	0.062	3.6498E-08	2
**Car4**	2.0525	0.288	0.081	2.6469E-06	2
**Kyat3**	2.0473	0.252	0.061	2.3545E-06	2
**Ccdc85a**	2.0458	0.315	0.090	4.2447E-07	2
**Cntfr**	2.0391	0.838	0.287	2.5930E-28	2
**Aldh6a1**	2.0378	0.928	0.546	6.4125E-32	2
**Macrod1**	2.0365	0.874	0.345	3.3082E-29	2

Table 1

**Pfkfb1**	2.0365	0.234	0.061	5.1036E-05	2
**Dhdh**	2.0309	0.423	0.110	1.6366E-12	2
**Prex2**	2.0309	0.405	0.115	9.9177E-11	2
**Hibch**	2.0272	0.658	0.193	2.0920E-21	2
**Pgm1**	2.0260	0.297	0.079	5.7939E-07	2
**Kng2**	2.0214	0.378	0.098	8.4800E-11	2
**Fmo1**	2.0199	0.640	0.250	8.2833E-17	2
**Fndc5**	2.0191	0.153	0.039	3.2028E-02	2
**Idh3a**	2.0182	0.991	0.745	1.0416E-40	2
**Decr1**	2.0177	0.865	0.505	1.9788E-23	2
**Papss2**	2.0165	0.171	0.044	8.7106E-03	2
**Kif26a**	2.0145	0.216	0.044	2.3754E-06	2
**Cebpa**	2.0110	0.937	0.640	8.9884E-32	2
**Cd36**	2.0108	0.991	0.834	2.1666E-40	2
**Etfdh**	2.0096	0.838	0.365	3.6987E-25	2
**Galnt15**	2.0084	0.297	0.086	2.4509E-06	2
**Adipoq**	2.0055	1.000	0.775	1.2336E-41	2
**Tmcc3**	2.0044	0.550	0.145	1.7253E-17	2
**Nrg4**	2.0000	0.559	0.179	1.5991E-15	2
**Radil**	2.0000	0.171	0.044	8.7106E-03	2
**Cdh23**	2.0000	0.153	0.039	3.2576E-02	2
**Impdh1**	1.9972	0.946	0.514	1.0061E-32	2
**Il15ra**	1.9936	0.396	0.117	1.0942E-09	2
**Fzd4**	1.9923	0.550	0.179	3.1716E-14	2
**Plcd3**	1.9905	0.252	0.078	4.9301E-04	2
**Prrx2**	1.9862	0.198	0.054	2.7898E-03	2
**Nos3**	1.9821	0.306	0.083	2.8138E-07	2
**Aacs**	1.9811	0.414	0.103	8.9956E-13	2
**Nudt13**	1.9769	0.234	0.069	7.9146E-04	2
**Pcx**	1.9730	0.865	0.402	1.3509E-24	2
**Tcim**	1.9727	0.658	0.228	6.9000E-19	2
**Slc25a19**	1.9714	0.477	0.145	2.6977E-12	2
**Myo5b**	1.9714	0.198	0.052	1.5732E-03	2
**Hadhb**	1.9697	0.964	0.542	6.9704E-35	2
**Bckdhb**	1.9684	0.640	0.231	6.0291E-17	2
**Sfrp1**	1.9676	0.270	0.059	5.1174E-08	2
**Gstk1**	1.9625	0.162	0.042	2.3177E-02	2
**Gprc5b**	1.9556	0.315	0.083	8.3763E-08	2
**Mief2**	1.9556	0.234	0.066	2.6687E-04	2
**Bckdha**	1.9524	0.964	0.633	2.0993E-31	2
**Agmo**	1.9521	0.297	0.078	3.2469E-07	2
**Mrgprf**	1.9521	0.288	0.074	4.8291E-07	2
**Galt**	1.9511	0.207	0.066	1.5505E-02	2
**Adra1d**	1.9467	0.477	0.137	4.0666E-13	2

Table 1

**Sncaip**	1.9456	0.207	0.056	1.0737E-03	2
**Podxl**	1.9411	0.288	0.074	4.9722E-07	2
**Trim68**	1.9411	0.153	0.039	3.4399E-02	2
**Atp1a2**	1.9405	0.937	0.677	4.2223E-30	2
**Synm**	1.9386	0.198	0.051	9.6559E-04	2
**Ech1**	1.9378	0.982	0.726	5.5102E-34	2
**Amy1**	1.9296	0.288	0.078	1.5083E-06	2
**Rasgrp3**	1.9296	0.162	0.041	1.3743E-02	2
**Acadl**	1.9295	0.982	0.910	7.1277E-39	2
**Mcur1**	1.9287	0.793	0.375	2.8827E-20	2
**Csad**	1.9276	0.784	0.334	1.3814E-22	2
**Ntrk2**	1.9255	0.595	0.177	1.1125E-17	2
**Dbt**	1.9250	0.541	0.176	7.8737E-14	2
**Gpt**	1.9232	0.577	0.181	6.5356E-16	2
**Timm17a**	1.9232	0.252	0.069	4.4196E-05	2
**Fasn**	1.9212	0.550	0.189	1.7599E-13	2
**Acot11**	1.9203	0.324	0.098	1.0181E-06	2
**Dpyd**	1.9203	0.189	0.046	8.1958E-04	2
**Shpk**	1.9203	0.189	0.047	1.4616E-03	2
**Slc36a2**	1.9197	0.937	0.564	2.5613E-28	2
**Rmdn1**	1.9195	0.423	0.125	1.2732E-10	2
**Nr1h3**	1.9195	0.414	0.123	5.9311E-10	2
**4931406C07Rik**	1.9114	0.658	0.265	7.1331E-16	2
**Echs1**	1.9110	0.973	0.723	6.7874E-33	2
**Mmut**	1.9106	0.306	0.091	3.6943E-06	2
**Rbp7**	1.9066	0.432	0.145	4.0852E-09	2
**Lpin1**	1.9062	0.757	0.312	2.0271E-19	2
**Sox7**	1.9005	0.234	0.062	1.1564E-04	2
**Nabp1**	1.9005	0.225	0.059	1.7943E-04	2
**Ppargc1b**	1.8955	0.775	0.301	1.7718E-23	2
**Cpn2**	1.8845	0.225	0.062	4.9947E-04	2
**Gpx3**	1.8822	1.000	0.970	1.3221E-48	2
**Art3**	1.8822	0.324	0.100	1.7258E-06	2
**Wnt10b**	1.8790	0.162	0.042	2.5445E-02	2
**C030006K11Rik**	1.8771	0.640	0.213	2.5206E-17	2
**Pxmp2**	1.8766	0.315	0.090	6.1054E-07	2
**Cers5**	1.8745	0.180	0.051	1.9154E-02	2
**Fdx1**	1.8732	0.694	0.270	1.1194E-17	2
**Gk**	1.8688	0.432	0.125	5.1951E-11	2
**S100a1**	1.8622	0.991	0.606	7.1789E-33	2
**Mccc2**	1.8614	0.414	0.137	9.6664E-09	2
**Dglucy**	1.8604	0.261	0.074	5.1825E-05	2
**G0s2**	1.8601	0.739	0.299	3.1490E-18	2
**Ndrg2**	1.8593	0.712	0.291	1.8943E-18	2

Table 1

**Cdo1**	1.8546	0.928	0.559	1.3571E-27	2
**Gpihbp1**	1.8511	0.739	0.287	1.7834E-19	2
**Inka2**	1.8447	0.261	0.069	1.3361E-05	2
**Aldh7a1**	1.8431	0.270	0.084	1.9595E-04	2
**Ppm1k**	1.8419	0.333	0.096	2.5616E-07	2
**Cyp2d22**	1.8382	0.405	0.120	1.4341E-09	2
**Ndufs3**	1.8360	0.477	0.171	3.9833E-10	2
**Phlda3**	1.8340	0.856	0.410	2.2211E-25	2
**Letmd1**	1.8333	0.676	0.289	1.2659E-15	2
**Sorbs1**	1.8301	0.532	0.182	3.2235E-12	2
**Cyp4b1**	1.8301	0.459	0.140	3.0555E-11	2
**Hlf**	1.8301	0.288	0.090	4.3410E-05	2
**Mamld1**	1.8301	0.225	0.068	2.4032E-03	2
**Gstt1**	1.8301	0.207	0.062	8.5610E-03	2
**Prrg2**	1.8301	0.207	0.064	1.3095E-02	2
**Acad12**	1.8301	0.153	0.039	3.8338E-02	2
**Lep**	1.8301	0.171	0.049	4.7656E-02	2
**Acads**	1.8278	0.919	0.600	1.6655E-26	2
**Cd300lg**	1.8271	0.703	0.301	3.0023E-16	2
**Chpt1**	1.8194	0.883	0.432	1.2541E-24	2
**Cav1**	1.8194	0.640	0.257	3.0450E-14	2
**Plekha4**	1.8189	0.261	0.086	1.3576E-03	2
**Rpusd3**	1.8146	0.261	0.074	5.8800E-05	2
**Slfn5**	1.8130	0.342	0.132	1.0589E-04	2
**Pdk2**	1.8129	0.820	0.426	1.8288E-21	2
**Wdr45**	1.8074	0.180	0.051	2.0863E-02	2
**Aldh4a1**	1.8044	0.523	0.184	2.0645E-11	2
**Slc9a3r2**	1.8042	0.838	0.368	2.5173E-23	2
**Fah**	1.8021	0.243	0.083	6.5241E-03	2
**Adam11**	1.7961	0.216	0.062	2.3192E-03	2
**Etfb**	1.7954	1.000	0.845	4.8576E-37	2
**Mmachc**	1.7935	0.216	0.064	3.7431E-03	2
**Hsdl2**	1.7916	0.730	0.274	6.8234E-19	2
**Mfn2**	1.7870	0.396	0.135	1.9103E-07	2
**Pdcl3**	1.7870	0.189	0.049	3.1047E-03	2
**Hoxa2**	1.7843	0.243	0.074	1.0449E-03	2
**Dnajc15**	1.7822	0.757	0.328	6.9793E-19	2
**Clock**	1.7812	0.243	0.066	9.9916E-05	2
**Pcmtd1**	1.7812	0.234	0.068	5.9462E-04	2
**Pgap2**	1.7812	0.234	0.068	6.3815E-04	2
**Fmc1**	1.7806	0.874	0.432	7.1733E-24	2
**Pcca**	1.7790	0.459	0.157	1.0276E-09	2
**Gchfr**	1.7776	0.216	0.068	9.0023E-03	2
**Myoc**	1.7776	0.207	0.066	2.3623E-02	2

Table 1

**Bri3**	1.7752	0.766	0.316	2.5498E-19	2
**Npr1**	1.7746	0.450	0.152	2.2755E-09	2
**Immp2l**	1.7746	0.270	0.081	9.7973E-05	2
**Vegfb**	1.7735	0.694	0.292	8.2624E-15	2
**Ednrb**	1.7701	0.423	0.123	2.9757E-10	2
**Suclg1**	1.7666	0.883	0.488	2.9001E-21	2
**Adgrf5**	1.7663	0.622	0.242	7.8572E-14	2
**Afg1l**	1.7630	0.378	0.125	5.4202E-07	2
**Enpep**	1.7630	0.180	0.052	3.5618E-02	2
**Cdh5**	1.7620	0.883	0.490	2.7303E-24	2
**Sult1a1**	1.7610	0.450	0.149	2.2245E-09	2
**Tmem88**	1.7597	0.495	0.169	1.2501E-10	2
**Opa1**	1.7590	0.252	0.079	9.1480E-04	2
**Palmd**	1.7579	0.360	0.108	1.3460E-07	2
**Depp1**	1.7566	0.847	0.459	1.7431E-20	2
**Yjefn3**	1.7545	0.225	0.076	2.2252E-02	2
**Dbi**	1.7544	1.000	0.861	1.8153E-35	2
**Hadha**	1.7521	0.973	0.608	2.2054E-30	2
**Enc1**	1.7521	0.288	0.090	5.7749E-05	2
**Axin2**	1.7521	0.279	0.088	1.4879E-04	2
**Acad11**	1.7506	0.568	0.221	4.4595E-11	2
**Ifi27**	1.7491	0.955	0.731	7.1675E-30	2
**Perm1**	1.7462	0.297	0.091	2.7403E-05	2
**Tie1**	1.7437	0.225	0.061	4.1690E-04	2
**Olfml1**	1.7418	0.261	0.083	7.3390E-04	2
**Ppp1r1a**	1.7397	0.441	0.150	7.6840E-09	2
**Idh3b**	1.7379	0.964	0.715	9.7988E-31	2
**Sparcl1**	1.7370	0.667	0.274	2.3120E-14	2
**Oxld1**	1.7336	0.378	0.127	6.7154E-07	2
**Acadsb**	1.7336	0.351	0.130	4.6752E-05	2
**Coasy**	1.7311	0.414	0.140	6.2429E-08	2
**Lama5**	1.7305	0.234	0.073	2.5763E-03	2
**Socs5**	1.7305	0.225	0.069	4.1197E-03	2
**Ier5l**	1.7270	0.288	0.088	3.9327E-05	2
**Slc2a4**	1.7235	0.775	0.377	1.2306E-17	2
**Slc25a33**	1.7232	0.297	0.095	5.1955E-05	2
**Pex16**	1.7211	0.459	0.160	3.4051E-09	2
**Pxdn**	1.7211	0.441	0.169	1.6275E-07	2
**Prss35**	1.7207	0.369	0.118	4.8989E-07	2
**Rnf150**	1.7184	0.225	0.073	9.3759E-03	2
**Prag1**	1.7171	0.342	0.105	9.4841E-07	2
**Pecr**	1.7162	0.423	0.157	4.9873E-07	2
**Acot13**	1.7157	0.577	0.216	4.5417E-12	2
**Mcf2l**	1.7124	0.369	0.111	6.7565E-08	2

Table 1

**Uqcrfs1**	1.7114	0.982	0.743	8.4043E-34	2
**Ndufa10**	1.7105	0.883	0.508	2.7405E-23	2
**Tspan12**	1.7105	0.234	0.069	1.1529E-03	2
**Heg1**	1.7102	0.577	0.225	1.1823E-11	2
**Etfa**	1.7093	0.883	0.541	5.3668E-22	2
**Hint2**	1.7069	0.604	0.220	1.1552E-13	2
**Egr1**	1.7056	0.748	0.318	6.4967E-17	2
**Nova2**	1.7045	0.243	0.081	5.5587E-03	2
**Agpat3**	1.7035	0.847	0.444	2.6877E-20	2
**Zbtb38**	1.7023	0.270	0.091	1.1804E-03	2
**Pccb**	1.7005	0.829	0.465	1.0248E-18	2
**Mgst1**	1.6988	0.766	0.289	1.0121E-19	2
**Mccc1**	1.6974	0.342	0.110	2.8242E-06	2
**Slc1a3**	1.6974	0.324	0.108	3.9441E-05	2
**Las1l**	1.6968	0.243	0.081	5.4439E-03	2
**Dld**	1.6951	0.928	0.573	1.3121E-23	2
**Paxx**	1.6951	0.450	0.164	5.2374E-08	2
**Slc22a5**	1.6951	0.279	0.088	1.7066E-04	2
**Gatd3a**	1.6944	0.739	0.394	6.3405E-15	2
**Mmp11**	1.6938	0.288	0.096	3.0821E-04	2
**Pdp2**	1.6899	0.369	0.115	1.9914E-07	2
**Cntnap1**	1.6881	0.252	0.071	1.3089E-04	2
**Aplnr**	1.6881	0.234	0.076	6.2376E-03	2
**Sdr39u1**	1.6871	0.405	0.145	4.9149E-07	2
**Aco1**	1.6869	0.640	0.277	3.4962E-13	2
**Svep1**	1.6854	0.505	0.179	2.3476E-10	2
**Ppm1m**	1.6854	0.486	0.199	4.1691E-08	2
**Kdr**	1.6848	0.676	0.282	1.9564E-13	2
**Tbc1d4**	1.6842	0.577	0.209	2.7204E-12	2
**Mecr**	1.6825	0.910	0.542	1.5801E-22	2
**Ehd2**	1.6781	0.820	0.373	7.3487E-21	2
**Tdrd7**	1.6781	0.225	0.071	6.8939E-03	2
**Orm1**	1.6781	0.180	0.051	2.4883E-02	2
**Emid1**	1.6781	0.189	0.057	4.1464E-02	2
**Sik2**	1.6759	0.658	0.280	3.8286E-13	2
**Lgals4**	1.6728	0.315	0.128	1.8914E-03	2
**Coq7**	1.6700	0.477	0.169	3.0967E-09	2
**Gbe1**	1.6698	0.658	0.296	1.0107E-12	2
**Prkra**	1.6666	0.216	0.068	1.1090E-02	2
**Rbm47**	1.6650	0.306	0.111	6.4237E-04	2
**Hk2**	1.6635	0.532	0.181	1.6547E-11	2
**Mmaa**	1.6612	0.333	0.113	1.9028E-05	2
**Mlycd**	1.6574	0.586	0.220	4.3087E-12	2
**Dlc1**	1.6560	0.559	0.223	2.5761E-10	2

Table 1

**Pdha1**	1.6542	0.991	0.750	3.9468E-31	2
**Dhrs7**	1.6521	0.631	0.242	5.0116E-14	2
**Sdhd**	1.6515	0.937	0.720	3.4676E-27	2
**Ndufa9**	1.6499	0.829	0.453	3.4585E-19	2
**Sgcd**	1.6495	0.369	0.123	1.6199E-06	2
**Isoc2a**	1.6457	0.865	0.429	1.3449E-20	2
**Prkag2**	1.6453	0.477	0.147	5.4544E-11	2
**Ndufs2**	1.6447	0.910	0.581	8.0638E-23	2
**Dlat**	1.6425	0.937	0.610	4.1473E-21	2
**Sgms1**	1.6424	0.198	0.062	4.2806E-02	2
**Prickle3**	1.6401	0.342	0.120	4.1234E-05	2
**Itpk1**	1.6386	0.586	0.230	7.6769E-12	2
**Nrp1**	1.6374	0.505	0.186	1.5030E-09	2
**Ddt**	1.6367	0.721	0.351	9.1813E-14	2
**Col8a2**	1.6358	0.495	0.177	1.3700E-09	2
**Ecsit**	1.6356	0.450	0.166	5.5838E-08	2
**Cd34**	1.6326	0.577	0.236	3.0334E-10	2
**Sod2**	1.6313	0.964	0.674	1.0829E-26	2
**Acox1**	1.6292	0.631	0.262	1.6898E-12	2
**Sulf1**	1.6284	0.288	0.101	9.0260E-04	2
**Pim3**	1.6259	0.631	0.265	2.8506E-12	2
**Gstz1**	1.6250	0.342	0.120	3.6617E-05	2
**Mical2**	1.6245	0.315	0.096	9.6192E-06	2
**Fmo2**	1.6236	0.207	0.068	3.9827E-02	2
**Esrra**	1.6215	0.748	0.372	4.9030E-15	2
**Amacr**	1.6204	0.378	0.122	2.8671E-07	2
**Scrn2**	1.6167	0.333	0.120	1.2031E-04	2
**Prickle2**	1.6167	0.198	0.059	1.9054E-02	2
**Mmd**	1.6162	0.505	0.167	8.6967E-11	2
**Ahcyl2**	1.6160	0.342	0.118	3.4131E-05	2
**Eya2**	1.6153	0.225	0.068	3.4720E-03	2
**Aars2**	1.6143	0.261	0.079	3.8353E-04	2
**Wnt5a**	1.6077	0.360	0.123	7.4818E-06	2
**Pcdh1**	1.6077	0.234	0.074	5.0952E-03	2
**Tnn**	1.6055	0.757	0.409	1.7469E-15	2
**Sdhb**	1.6046	1.000	0.892	1.9889E-34	2
**Idh3g**	1.6028	0.910	0.652	5.5257E-21	2
**Cav2**	1.6021	0.649	0.252	9.0731E-14	2
**Adamts15**	1.6006	0.243	0.076	2.0659E-03	2
**Stimate**	1.5995	0.216	0.071	2.7704E-02	2
**Aspn**	1.5991	0.559	0.225	5.0099E-10	2
**Igf1**	1.5974	0.739	0.312	1.6079E-16	2
**Hrct1**	1.5969	0.288	0.095	3.4294E-04	2
**Nnmt**	1.5956	0.369	0.128	4.7771E-06	2

Table 1

**Cd274**	1.5956	0.288	0.084	2.7619E-05	2
**Kank3**	1.5946	0.550	0.216	8.2391E-10	2
**Gm5617**	1.5946	0.324	0.120	5.3625E-04	2
**Ndufab1**	1.5945	0.919	0.588	1.3247E-23	2
**Bscl2**	1.5936	0.486	0.182	1.5871E-08	2
**Lpar6**	1.5934	0.351	0.118	9.1214E-06	2
**Rhoj**	1.5919	0.306	0.101	1.0821E-04	2
**Naprt**	1.5919	0.297	0.103	4.9063E-04	2
**F11r**	1.5912	0.523	0.191	1.0964E-09	2
**Atf5**	1.5895	0.730	0.324	2.8435E-15	2
**Cluh**	1.5891	0.820	0.456	2.0505E-15	2
**Sh2b2**	1.5879	0.631	0.280	3.4478E-12	2
**Hoxa5**	1.5879	0.261	0.084	1.3310E-03	2
**Cyyr1**	1.5872	0.297	0.106	1.0004E-03	2
**Ndufs1**	1.5833	0.649	0.252	6.8835E-14	2
**Ebf3**	1.5821	0.243	0.088	2.8004E-02	2
**Cyp27a1**	1.5815	0.495	0.186	5.2791E-09	2
**Sco2**	1.5810	0.342	0.123	6.7173E-05	2
**Gpr4**	1.5810	0.189	0.057	4.8053E-02	2
**Amotl2**	1.5798	0.802	0.383	5.6610E-17	2
**Cped1**	1.5797	0.378	0.128	1.2577E-06	2
**Cavin2**	1.5787	0.892	0.556	1.0553E-19	2
**Meox2**	1.5785	0.423	0.140	3.3751E-08	2
**Dusp4**	1.5779	0.550	0.213	2.0433E-09	2
**S100a13**	1.5772	0.892	0.498	3.3994E-21	2
**Ptprg**	1.5718	0.252	0.093	2.6827E-02	2
**Ndufa7**	1.5707	0.550	0.223	7.9327E-10	2
**Egfl7**	1.5698	0.766	0.356	5.7119E-16	2
**Rgs5**	1.5690	0.676	0.350	1.4962E-10	2
**Pde3b**	1.5688	0.595	0.208	2.7531E-12	2
**Cgnl1**	1.5688	0.541	0.221	4.9952E-09	2
**Tmem53**	1.5636	0.351	0.123	2.8005E-05	2
**Entpd5**	1.5634	0.532	0.209	2.6202E-09	2
**Parm1**	1.5609	0.216	0.061	2.0039E-03	2
**Txnip**	1.5604	0.991	0.779	5.4212E-32	2
**Ccdc3**	1.5589	0.667	0.299	4.8472E-12	2
**Ushbp1**	1.5584	0.631	0.269	8.1871E-12	2
**Sept4**	1.5580	0.387	0.145	1.1532E-05	2
**Prkch**	1.5571	0.252	0.090	1.3428E-02	2
**Megf6**	1.5559	0.342	0.108	4.2725E-06	2
**Cmbl**	1.5552	0.225	0.074	1.9366E-02	2
**Epha2**	1.5544	0.468	0.191	7.7096E-07	2
**Jun**	1.5525	0.901	0.524	2.6025E-23	2
**Crls1**	1.5525	0.396	0.140	2.4841E-06	2

Table 1

**Lrrc15**	1.5483	0.306	0.118	2.2484E-03	2
**Adipor2**	1.5463	0.450	0.179	1.0330E-06	2
**Rasip1**	1.5460	0.667	0.331	1.1657E-10	2
**Adgrl4**	1.5456	0.351	0.144	1.0156E-03	2
**Ece1**	1.5454	0.559	0.215	3.1968E-10	2
**Lama4**	1.5451	0.396	0.147	6.4415E-06	2
**Gja4**	1.5443	0.261	0.100	2.7012E-02	2
**Col4a1**	1.5426	0.964	0.767	8.6429E-26	2
**Vldlr**	1.5421	0.523	0.206	1.3113E-08	2
**Pick1**	1.5406	0.252	0.086	7.1489E-03	2
**Sfxn5**	1.5406	0.207	0.066	3.2728E-02	2
**Dpep1**	1.5406	0.234	0.083	3.6258E-02	2
**Hfe**	1.5375	0.333	0.117	7.9433E-05	2
**Ccs**	1.5370	0.495	0.189	2.1856E-08	2
**Esam**	1.5365	0.739	0.294	1.2151E-16	2
**H2afy2**	1.5346	0.333	0.117	8.6470E-05	2
**Sdhc**	1.5345	0.811	0.490	1.0894E-15	2
**Rhobtb1**	1.5337	0.306	0.108	6.3017E-04	2
**Shmt1**	1.5332	0.514	0.216	5.1105E-08	2
**Tns1**	1.5328	0.973	0.797	5.1901E-25	2
**Dpt**	1.5327	0.559	0.182	1.4649E-12	2
**Prss23**	1.5316	0.396	0.137	1.2681E-06	2
**Fuom**	1.5305	0.315	0.105	7.7992E-05	2
**Csrp2**	1.5275	0.333	0.106	1.2220E-05	2
**Cebpb**	1.5263	0.964	0.672	6.2417E-25	2
**Wfdc1**	1.5247	0.306	0.105	2.6896E-04	2
**Coq3**	1.5247	0.315	0.115	6.2905E-04	2
**Cpxm1**	1.5243	0.351	0.128	1.4072E-04	2
**Dlst**	1.5231	0.928	0.703	1.3635E-20	2
**Cbr1**	1.5231	0.568	0.240	1.1232E-08	2
**Btd**	1.5220	0.387	0.139	5.0971E-06	2
**Acat1**	1.5206	0.838	0.497	1.1205E-15	2
**Nr2f6**	1.5204	0.712	0.333	1.2165E-13	2
**Dhrs7b**	1.5187	0.532	0.255	1.0752E-06	2
**Tns2**	1.5178	0.505	0.206	8.7429E-08	2
**Tagln**	1.5173	0.541	0.211	3.3527E-09	2
**Grasp**	1.5169	0.342	0.120	5.2642E-05	2
**Ifngr1**	1.5160	0.306	0.103	2.2221E-04	2
**Mapk1ip1**	1.5156	0.360	0.135	9.4334E-05	2
**Idh2**	1.5133	0.946	0.725	1.3553E-23	2
**Fermt2**	1.5132	0.495	0.196	4.1169E-08	2
**Hint3**	1.5099	0.378	0.152	1.3870E-04	2
**Tmem205**	1.5081	0.685	0.299	3.8162E-12	2
**Slc2a8**	1.5081	0.297	0.108	2.2310E-03	2

Table 1

**Fahd2a**	1.5058	0.333	0.115	6.6893E-05	2
**Fh1**	1.5031	0.856	0.542	8.6133E-16	2
**Ak3**	1.5025	0.658	0.274	3.0829E-12	2
**Sdha**	1.5020	0.865	0.559	7.8710E-18	2
**Crat**	1.5004	0.919	0.698	7.1379E-22	2
**Ano1**	1.4999	0.279	0.098	2.5545E-03	2
**Cox6c**	1.4981	0.685	0.341	4.1769E-11	2
**Itih5**	1.4975	0.523	0.204	6.8552E-09	2
**Yap1**	1.4975	0.288	0.093	2.5255E-04	2
**Shb**	1.4975	0.297	0.098	2.7702E-04	2
**Lamb1**	1.4945	0.631	0.280	1.5864E-10	2
**Igfbp6**	1.4927	0.550	0.218	1.0753E-09	2
**Cs**	1.4921	0.982	0.904	1.5231E-27	2
**Afap1l1**	1.4913	0.486	0.177	1.9463E-08	2
**Coq9**	1.4911	0.883	0.611	1.6712E-16	2
**Hoxa4**	1.4906	0.351	0.132	1.2780E-04	2
**Smim26**	1.4890	0.342	0.118	4.2162E-05	2
**Fst**	1.4890	0.297	0.108	1.8862E-03	2
**Clybl**	1.4881	0.550	0.215	1.1052E-09	2
**C130074G19Rik**	1.4875	0.559	0.252	6.6080E-08	2
**Uqcrc2**	1.4874	0.865	0.564	6.0142E-19	2
**Loxl1**	1.4822	0.586	0.255	4.2973E-09	2
**Suclg2**	1.4796	0.505	0.203	1.4927E-07	2
**Jam3**	1.4782	0.297	0.096	2.0676E-04	2
**Caskin2**	1.4731	0.297	0.103	7.3574E-04	2
**Grrp1**	1.4731	0.297	0.111	3.7954E-03	2
**Cfl2**	1.4707	0.495	0.177	6.7122E-09	2
**Ogdh**	1.4679	0.991	0.894	1.3621E-25	2
**Uqcrc1**	1.4675	1.000	0.953	2.3505E-34	2
**Tecpr1**	1.4675	0.369	0.150	5.3521E-04	2
**Pde4a**	1.4646	0.486	0.206	8.2362E-07	2
**Nit2**	1.4639	0.351	0.128	9.2323E-05	2
**Pakap**	1.4619	0.757	0.368	4.3423E-14	2
**Commd6**	1.4618	0.369	0.133	2.7066E-05	2
**Ttc38**	1.4608	0.514	0.220	4.0998E-07	2
**Rab3ip**	1.4608	0.324	0.140	1.7779E-02	2
**Alkbh7**	1.4590	0.378	0.144	5.6380E-05	2
**Akt2**	1.4584	0.784	0.378	4.8317E-16	2
**Fam171a2**	1.4581	0.378	0.127	2.0123E-06	2
**Nampt**	1.4573	0.396	0.145	9.8459E-06	2
**Npepl1**	1.4571	0.550	0.231	1.3076E-08	2
**Syne2**	1.4569	0.279	0.098	2.9769E-03	2
**Fbln2**	1.4557	0.369	0.149	2.8099E-04	2
**Nudt8**	1.4546	0.297	0.110	3.0530E-03	2

Table 1

**Emc8**	1.4542	0.423	0.164	6.7560E-06	2
**Dmac2**	1.4528	0.378	0.171	1.8218E-03	2
**S1pr1**	1.4522	0.396	0.154	2.7681E-05	2
**Ndufv1**	1.4517	0.991	0.914	9.6613E-30	2
**Mcee**	1.4516	0.279	0.113	4.3691E-02	2
**Cldn5**	1.4510	0.739	0.353	2.8642E-12	2
**Coq10a**	1.4498	0.595	0.270	1.8410E-08	2
**Clpb**	1.4486	0.541	0.236	7.3452E-08	2
**Nudt7**	1.4482	0.333	0.127	7.5249E-04	2
**Ndufs5**	1.4467	0.459	0.186	2.3709E-06	2
**Nr4a1**	1.4465	0.703	0.319	2.6121E-12	2
**Yif1a**	1.4458	0.477	0.198	1.7119E-06	2
**Sgcb**	1.4436	0.378	0.137	1.7455E-05	2
**Spg20**	1.4436	0.396	0.152	2.7400E-05	2
**Acat2**	1.4427	0.468	0.223	7.6348E-05	2
**Epha4**	1.4420	0.234	0.076	9.5955E-03	2
**Sod3**	1.4415	0.964	0.603	1.7312E-25	2
**Hspa12b**	1.4410	0.423	0.162	4.0708E-06	2
**Epas1**	1.4408	0.883	0.534	9.5733E-17	2
**Chchd10**	1.4401	0.874	0.639	1.8621E-16	2
**Strip1**	1.4393	0.243	0.088	3.7999E-02	2
**Ptgr2**	1.4354	0.766	0.404	2.3248E-11	2
**Stx18**	1.4352	0.297	0.110	3.2673E-03	2
**Rbpms2**	1.4352	0.270	0.105	3.4536E-02	2
**Ahr**	1.4341	0.288	0.108	7.2135E-03	2
**Smim20**	1.4341	0.279	0.111	3.8568E-02	2
**Alas1**	1.4336	0.829	0.515	3.5038E-13	2
**Idh1**	1.4321	0.514	0.226	1.2835E-06	2
**Gstm1**	1.4271	0.441	0.167	1.3337E-06	2
**2300009A05Rik**	1.4255	0.514	0.181	3.1899E-09	2
**Serping1**	1.4231	0.577	0.230	9.6965E-10	2
**Hsd17b4**	1.4198	0.757	0.360	5.4563E-14	2
**Cyc1**	1.4157	0.982	0.921	1.0823E-29	2
**Ndufs7**	1.4150	0.991	0.885	1.1200E-26	2
**Mfap4**	1.4150	0.658	0.247	8.5145E-13	2
**Tspan18**	1.4150	0.640	0.247	1.3086E-11	2
**Pex19**	1.4150	0.523	0.243	2.1389E-06	2
**Dip2a**	1.4150	0.297	0.113	6.2719E-03	2
**Tnfsf12**	1.4150	0.279	0.108	2.1517E-02	2
**Adck1**	1.4150	0.243	0.088	4.0493E-02	2
**Tmem86a**	1.4150	0.252	0.095	4.7007E-02	2
**Atpaf2**	1.4102	0.748	0.365	4.4964E-12	2
**Cox6a1**	1.4082	1.000	0.990	9.1279E-36	2
**Myh11**	1.4055	0.441	0.204	2.1147E-04	2

Table 1

**Tmed5**	1.4033	0.459	0.182	2.2327E-06	2
**Cd248**	1.4022	0.405	0.155	2.6513E-05	2
**Tmem100**	1.4022	0.369	0.150	5.7579E-04	2
**Bcar1**	1.3987	0.333	0.132	2.4110E-03	2
**Ptprm**	1.3987	0.333	0.137	4.9885E-03	2
**Snta1**	1.3985	0.541	0.242	7.1951E-07	2
**Actr8**	1.3984	0.333	0.128	1.7106E-03	2
**Hsd17b10**	1.3981	0.793	0.466	6.2120E-12	2
**Kcnb1**	1.3967	0.297	0.115	8.3808E-03	2
**Flt1**	1.3954	0.730	0.358	4.1862E-12	2
**Ndufv2**	1.3948	0.784	0.416	2.5657E-13	2
**Acss1**	1.3937	0.441	0.184	2.6562E-05	2
**Lgr6**	1.3930	0.387	0.186	8.1539E-03	2
**Vwa8**	1.3923	0.676	0.309	2.6549E-10	2
**Map1lc3a**	1.3906	0.892	0.598	3.5194E-18	2
**Epb41l1**	1.3893	0.631	0.277	1.7501E-10	2
**Kdm4c**	1.3886	0.234	0.081	3.4178E-02	2
**Sco1**	1.3881	0.360	0.150	1.6641E-03	2
**Atp5f1**	1.3873	0.703	0.318	1.8901E-12	2
**Adamtsl3**	1.3865	0.378	0.135	1.9813E-05	2
**Mrm3**	1.3865	0.387	0.149	7.6521E-05	2
**Rhoq**	1.3831	0.441	0.188	2.6099E-05	2
**Mtg1**	1.3826	0.342	0.132	7.1326E-04	2
**Plin2**	1.3804	0.685	0.351	1.9883E-09	2
**Rab12**	1.3776	0.505	0.223	3.0471E-06	2
**Hic1**	1.3766	0.297	0.108	3.0476E-03	2
**Lap3**	1.3764	0.586	0.262	4.5095E-08	2
**Ptcd1**	1.3760	0.559	0.208	8.3266E-10	2
**Itpkb**	1.3755	0.495	0.208	1.1844E-06	2
**Uqcr11**	1.3732	0.973	0.816	2.0294E-25	2
**Oxsm**	1.3724	0.378	0.150	1.9331E-04	2
**Col12a1**	1.3720	0.730	0.405	6.1772E-10	2
**Miga2**	1.3714	0.748	0.372	4.4670E-12	2
**Tm4sf1**	1.3711	0.568	0.265	1.7290E-07	2
**Plaat3**	1.3705	0.856	0.502	1.1342E-14	2
**Timm10**	1.3677	0.495	0.203	5.1488E-07	2
**Phactr2**	1.3677	0.450	0.179	3.3989E-06	2
**Itpr1**	1.3661	0.532	0.242	1.4721E-06	2
**Itga11**	1.3644	0.676	0.307	2.1275E-11	2
**Fzd1**	1.3638	0.532	0.235	6.7944E-07	2
**Trpt1**	1.3632	0.333	0.120	3.4664E-04	2
**Prkaca**	1.3632	0.459	0.225	4.8610E-04	2
**Afdn**	1.3632	0.297	0.122	2.9977E-02	2
**Scp2**	1.3623	0.919	0.688	1.4352E-16	2

Table 1

**Aebp1**	1.3619	0.514	0.221	1.2080E-06	2
**Abcd3**	1.3606	0.396	0.152	6.0232E-05	2
**Adcy6**	1.3592	0.306	0.115	3.4507E-03	2
**Postn**	1.3587	0.748	0.405	2.7124E-12	2
**Zbtb20**	1.3585	0.405	0.144	2.6552E-06	2
**Sntb2**	1.3577	0.306	0.110	1.5501E-03	2
**Pcdhgc3**	1.3569	0.459	0.208	8.3203E-05	2
**Gpc4**	1.3561	0.360	0.142	5.5253E-04	2
**Lias**	1.3552	0.414	0.188	8.6082E-04	2
**Tufm**	1.3547	0.523	0.236	2.0538E-06	2
**Kdm6b**	1.3528	0.279	0.108	2.6483E-02	2
**Tmem201**	1.3520	0.414	0.171	8.5983E-05	2
**Zeb1**	1.3509	0.351	0.132	3.2911E-04	2
**Igf2**	1.3507	0.811	0.492	5.1725E-13	2
**Gdf10**	1.3499	0.459	0.203	4.7517E-05	2
**Apmap**	1.3494	0.351	0.137	8.9776E-04	2
**Myl9**	1.3482	0.685	0.345	1.7507E-09	2
**Cox10**	1.3446	0.243	0.088	4.6163E-02	2
**Gss**	1.3418	0.342	0.145	5.6346E-03	2
**Slc25a22**	1.3416	0.459	0.189	8.1544E-06	2
**Coq5**	1.3409	0.829	0.522	1.1766E-13	2
**Pecam1**	1.3370	0.387	0.159	3.2324E-04	2
**Poldip2**	1.3364	0.577	0.318	6.3794E-05	2
**Ppp1r14a**	1.3345	0.414	0.162	3.7761E-05	2
**Ndufb6**	1.3344	1.000	0.912	4.3356E-29	2
**Emc9**	1.3305	0.414	0.181	4.1476E-04	2
**Fam210a**	1.3300	0.559	0.284	1.1597E-05	2
**Tenm4**	1.3296	0.342	0.113	3.0522E-05	2
**Comt**	1.3286	0.532	0.226	5.8615E-07	2
**Ptges2**	1.3285	0.586	0.243	5.1316E-09	2
**Acaa1a**	1.3254	0.748	0.394	2.2351E-10	2
**Pxmp4**	1.3239	0.333	0.144	1.3360E-02	2
**Ptn**	1.3230	0.541	0.311	6.6018E-03	2
**Zfp703**	1.3222	0.811	0.456	5.9834E-14	2
**Trim16**	1.3219	0.333	0.135	4.7277E-03	2
**Prodh**	1.3193	0.360	0.150	3.1214E-03	2
**Cox7b**	1.3151	0.937	0.742	7.5354E-19	2
**Snx21**	1.3146	0.541	0.296	2.1073E-04	2
**Aldoa**	1.3140	0.973	0.858	2.2782E-22	2
**Ablim1**	1.3135	0.351	0.152	6.0047E-03	2
**Acvrl1**	1.3132	0.333	0.130	2.2735E-03	2
**Taco1**	1.3124	0.441	0.182	2.8361E-05	2
**Clpx**	1.3110	0.703	0.338	1.0899E-09	2
**Acsf3**	1.3107	0.486	0.220	2.5285E-05	2

Table 1

**Kyat1**	1.3107	0.333	0.123	8.4943E-04	2
**Ltbp4**	1.3087	0.766	0.367	1.1910E-13	2
**Ndufb5**	1.3072	0.901	0.672	2.8456E-15	2
**Ndufs4**	1.3072	0.883	0.642	1.5111E-15	2
**Ndufa12**	1.3071	0.829	0.561	2.4020E-11	2
**Nrarp**	1.3061	0.378	0.155	8.2958E-04	2
**Bcat2**	1.3056	0.757	0.410	1.0875E-09	2
**Parp3**	1.3050	0.559	0.233	3.8758E-08	2
**Serhl**	1.3040	0.532	0.225	5.7848E-07	2
**Tob2**	1.3038	0.685	0.321	5.9822E-10	2
**Selenon**	1.3031	0.477	0.191	2.1818E-06	2
**Snrk**	1.3022	0.910	0.671	2.4341E-16	2
**Pptc7**	1.2996	0.468	0.189	6.0567E-06	2
**Ndufaf5**	1.2996	0.297	0.120	2.7783E-02	2
**Pdzd2**	1.2964	0.586	0.299	4.0165E-06	2
**Atp5d**	1.2921	0.991	0.988	3.8049E-32	2
**Cdh13**	1.2917	0.405	0.169	4.0953E-04	2
**Lonp2**	1.2914	0.613	0.279	2.1778E-08	2
**Mmrn2**	1.2907	0.414	0.154	9.6654E-06	2
**Abhd11**	1.2902	0.550	0.240	3.3384E-07	2
**C1qtnf3**	1.2883	0.577	0.233	1.1584E-08	2
**Usp21**	1.2879	0.315	0.125	8.3523E-03	2
**Fam234a**	1.2838	0.450	0.204	2.3783E-04	2
**Clec14a**	1.2833	0.423	0.199	2.8453E-03	2
**Hipk3**	1.2824	0.748	0.417	1.6734E-10	2
**Calu**	1.2824	0.631	0.363	3.4228E-06	2
**Lrfn4**	1.2818	0.261	0.096	3.0365E-02	2
**Pdhx**	1.2815	0.468	0.208	4.4694E-05	2
**Ralgapa2**	1.2815	0.450	0.203	2.1876E-04	2
**Wtip**	1.2812	0.306	0.117	6.5704E-03	2
**Mkrn2**	1.2812	0.306	0.118	7.4105E-03	2
**Tmem65**	1.2811	0.874	0.507	2.1450E-14	2
**Shank3**	1.2802	0.865	0.547	7.0499E-11	2
**Itpr2**	1.2795	0.721	0.346	2.4898E-10	2
**Ccm2l**	1.2775	0.324	0.133	1.2378E-02	2
**Pon3**	1.2755	0.450	0.203	2.1559E-04	2
**Mocs2**	1.2755	0.423	0.186	6.5532E-04	2
**Tob1**	1.2754	0.667	0.324	1.2904E-08	2
**Ptprz1**	1.2746	0.342	0.149	1.4896E-02	2
**Pald1**	1.2743	0.739	0.400	5.7733E-10	2
**Dolpp1**	1.2742	0.315	0.127	1.2399E-02	2
**Trp53inp2**	1.2740	0.640	0.324	5.3005E-07	2
**Hsd17b12**	1.2732	0.829	0.495	4.1118E-13	2
**Sirt3**	1.2721	0.378	0.155	1.0163E-03	2

Table 1

**Dact3**	1.2700	0.387	0.160	8.1408E-04	2
**Acot2**	1.2700	0.378	0.166	3.6500E-03	2
**Ndufa5**	1.2687	0.874	0.684	6.9039E-12	2
**Cisd1**	1.2682	0.396	0.177	2.8544E-03	2
**Klf9**	1.2671	0.667	0.323	3.4660E-09	2
**Cd151**	1.2668	0.658	0.346	1.7779E-08	2
**Mdh1**	1.2663	0.928	0.809	4.5375E-16	2
**Tnxb**	1.2662	0.459	0.203	7.5042E-05	2
**C4b**	1.2653	0.405	0.191	3.4921E-03	2
**Ciapin1**	1.2648	0.495	0.252	4.9151E-04	2
**Smpdl3a**	1.2640	0.559	0.240	3.1818E-07	2
**Spr**	1.2638	0.559	0.272	2.2836E-06	2
**Pdgfd**	1.2630	0.396	0.169	8.4059E-04	2
**Timm9**	1.2630	0.324	0.142	4.3524E-02	2
**Vegfa**	1.2622	0.784	0.426	3.3900E-10	2
**Pakap.1**	1.2618	0.631	0.309	1.4486E-07	2
**Uqcr10**	1.2605	0.955	0.850	1.8568E-20	2
**Kremen1**	1.2594	0.541	0.230	2.9169E-07	2
**Dok4**	1.2566	0.450	0.174	6.1912E-06	2
**AU022252**	1.2554	0.360	0.159	1.0493E-02	2
**Ndufs6**	1.2553	0.631	0.316	2.9450E-07	2
**Tmem135**	1.2533	0.459	0.182	1.0207E-05	2
**Slc27a1**	1.2528	0.622	0.299	1.3276E-07	2
**Tspan9**	1.2521	0.351	0.150	7.9828E-03	2
**Ndufs8**	1.2516	0.964	0.838	1.2463E-21	2
**Acyp2**	1.2500	0.631	0.336	5.8136E-07	2
**Zfp423**	1.2497	0.450	0.225	2.4047E-03	2
**Trim2**	1.2495	0.523	0.226	2.0632E-06	2
**Cry2**	1.2490	0.342	0.137	3.1833E-03	2
**Hmgcs1**	1.2487	0.559	0.231	2.0022E-08	2
**Ndufa1**	1.2485	0.874	0.586	7.8301E-13	2
**Nfia**	1.2468	0.559	0.267	5.6942E-06	2
**Ndufc2**	1.2455	0.982	0.917	1.3398E-24	2
**Pfkl**	1.2432	0.514	0.258	3.1153E-04	2
**Apba3**	1.2420	0.369	0.174	2.7262E-02	2
**Ppp2r5a**	1.2409	0.649	0.329	1.2459E-07	2
**Srpx**	1.2382	0.324	0.142	3.4391E-02	2
**Penk**	1.2367	0.396	0.179	2.9193E-03	2
**Decr2**	1.2350	0.486	0.235	2.3565E-04	2
**Itm2a**	1.2336	0.766	0.443	4.1327E-09	2
**Prkar2b**	1.2328	0.892	0.608	4.5464E-13	2
**Igfbp3**	1.2312	0.712	0.476	6.2964E-06	2
**Ppa2**	1.2306	0.432	0.203	1.7919E-03	2
**Gprc5c**	1.2306	0.342	0.145	1.3277E-02	2

Table 1

**Ctif**	1.2295	0.369	0.157	4.5824E-03	2
**Mpc2**	1.2292	0.784	0.593	5.6180E-08	2
**Cygb**	1.2286	0.396	0.186	7.3140E-03	2
**Ndufv3**	1.2284	0.964	0.838	1.3389E-18	2
**Mdh2**	1.2270	0.982	0.787	2.1962E-21	2
**Lamc1**	1.2265	0.784	0.534	1.3835E-10	2
**Clec11a**	1.2261	0.811	0.424	1.8993E-11	2
**Tesk1**	1.2260	0.342	0.155	4.1561E-02	2
**Ctso**	1.2252	0.279	0.106	3.0199E-02	2
**Ndufa8**	1.2221	0.901	0.706	7.1362E-16	2
**Zadh2**	1.2212	0.441	0.225	1.2872E-02	2
**Mdfic**	1.2198	0.459	0.221	1.0971E-03	2
**Zfp629**	1.2186	0.351	0.149	7.0989E-03	2
**Atoh8**	1.2186	0.360	0.160	1.5824E-02	2
**Robo1**	1.2182	0.459	0.211	3.7532E-04	2
**Immt**	1.2174	0.883	0.654	8.6323E-13	2
**Ccdc8**	1.2171	0.369	0.176	3.7622E-02	2
**Cd99l2**	1.2154	0.441	0.194	3.1013E-04	2
**Zfp36**	1.2153	0.595	0.292	1.4916E-06	2
**Ndufb8**	1.2145	0.937	0.755	2.3544E-15	2
**Nid1**	1.2134	0.505	0.270	1.0004E-03	2
**Samm50**	1.2101	0.757	0.497	7.0253E-09	2
**Cask**	1.2086	0.387	0.167	2.8451E-03	2
**Pmpcb**	1.2071	0.892	0.561	1.4465E-13	2
**Atp5b**	1.2060	1.000	0.953	3.8055E-29	2
**Pnpla8**	1.2056	0.658	0.358	3.4885E-07	2
**Fat4**	1.2048	0.369	0.154	2.1298E-03	2
**Chchd3**	1.2031	0.856	0.600	5.5413E-13	2
**Arhgef28**	1.2030	0.414	0.166	1.1479E-04	2
**2900026A02Rik**	1.2006	0.523	0.255	1.2672E-04	2
**Rab34**	1.1990	0.459	0.201	1.0283E-04	2
**Ahcyl1**	1.1979	0.946	0.704	8.4073E-16	2
**Gnpat**	1.1966	0.586	0.302	1.9823E-05	2
**Heyl**	1.1956	0.450	0.225	4.4011E-03	2
**Scd1**	1.1926	0.622	0.319	4.4154E-06	2
**Rhou**	1.1926	0.559	0.284	2.8334E-05	2
**Tjp1**	1.1926	0.468	0.243	3.4279E-03	2
**Gstt2**	1.1908	0.577	0.328	1.8456E-04	2
**Robo4**	1.1896	0.477	0.209	7.3088E-05	2
**Ccnd1**	1.1885	0.369	0.164	1.1290E-02	2
**Efnb1**	1.1885	0.378	0.174	1.5883E-02	2
**Trap1**	1.1866	0.613	0.378	1.0461E-04	2
**Atp5k**	1.1863	0.946	0.841	4.2594E-17	2
**Rassf8**	1.1818	0.586	0.311	2.1003E-05	2

Table 1

**Lims2**	1.1784	0.577	0.289	1.9792E-05	2
**Cox5b**	1.1756	1.000	0.990	1.2295E-28	2
**Glis2**	1.1750	0.387	0.188	2.9168E-02	2
**Rbx1**	1.1740	0.351	0.159	3.5357E-02	2
**Acaca**	1.1736	0.604	0.346	6.3607E-05	2
**Acss2**	1.1711	0.514	0.236	2.4434E-05	2
**Arhgef40**	1.1707	0.514	0.258	3.7866E-04	2
**Gsto1**	1.1699	0.685	0.382	2.2370E-07	2
**Atp5c1**	1.1696	0.748	0.417	1.3040E-09	2
**Angpt1**	1.1684	0.414	0.194	7.3040E-03	2
**Lmf1**	1.1671	0.360	0.150	3.9644E-03	2
**Lzts2**	1.1640	0.784	0.441	3.9930E-10	2
**Lpp**	1.1630	0.405	0.182	2.9176E-03	2
**Pex6**	1.1627	0.883	0.590	1.9371E-12	2
**Rarres2**	1.1613	0.667	0.343	2.9317E-07	2
**Ptgfrn**	1.1575	0.532	0.265	1.2455E-04	2
**Cdc42bpb**	1.1572	0.495	0.235	2.5653E-04	2
**Sik3**	1.1572	0.369	0.157	4.6890E-03	2
**Prrx1**	1.1544	0.820	0.475	2.8902E-10	2
**Pkn1**	1.1525	0.865	0.590	2.2270E-13	2
**Itga6**	1.1494	0.595	0.301	4.5304E-06	2
**Tmem106b**	1.1491	0.523	0.279	1.0234E-03	2
**Stk40**	1.1485	0.450	0.230	1.0237E-02	2
**March5**	1.1483	0.450	0.216	2.7965E-03	2
**Sowahc**	1.1476	0.414	0.181	1.6414E-03	2
**Egln1**	1.1465	0.676	0.400	3.8216E-07	2
**Sptbn1**	1.1445	0.991	0.848	1.1637E-22	2
**Atp5a1**	1.1444	1.000	0.993	1.9370E-27	2
**Ndufb9**	1.1443	0.667	0.436	2.0300E-04	2
**Dusp10**	1.1420	0.495	0.231	1.6626E-04	2
**Ptpn4**	1.1411	0.432	0.176	1.6136E-04	2
**Rragc**	1.1384	0.595	0.299	9.2121E-06	2
**Endog**	1.1378	0.414	0.208	2.2462E-02	2
**Efna1**	1.1363	0.432	0.208	6.9024E-03	2
**Slc25a11**	1.1356	0.658	0.390	1.8723E-06	2
**Ndufb10**	1.1343	1.000	0.966	2.0529E-25	2
**Ammecr1l**	1.1335	0.396	0.189	2.3570E-02	2
**Uqcrh**	1.1320	0.856	0.610	3.2622E-11	2
**Plekhh3**	1.1312	0.505	0.223	2.8030E-05	2
**Nnat**	1.1296	0.414	0.167	1.1171E-04	2
**Clcn4**	1.1296	0.387	0.169	5.2557E-03	2
**Fndc1**	1.1290	0.604	0.277	3.4651E-07	2
**2410131K14Rik**	1.1271	0.351	0.152	1.7805E-02	2
**Por**	1.1270	0.748	0.380	9.9444E-09	2

Table 1

**H2-Aa**	1.1247	0.477	0.265	1.8945E-02	2
**Grhpr**	1.1218	0.441	0.233	3.1643E-02	2
**Tbc1d20**	1.1215	0.739	0.476	2.9585E-06	2
**Sh3bp5**	1.1200	0.712	0.399	3.3805E-07	2
**2310061I04Rik**	1.1177	0.577	0.267	1.7621E-06	2
**Lrp5**	1.1170	0.441	0.213	3.6653E-03	2
**Xdh**	1.1131	0.423	0.199	5.2232E-03	2
**Notch4**	1.1131	0.414	0.198	1.0339E-02	2
**Pygb**	1.1131	0.414	0.206	2.8356E-02	2
**Myct1**	1.1095	0.414	0.181	1.7108E-03	2
**Ank2**	1.1091	0.559	0.252	8.8560E-07	2
**Aifm1**	1.1088	0.495	0.236	5.7126E-04	2
**Afg3l2**	1.1081	0.604	0.301	1.0455E-05	2
**Card19**	1.1075	0.604	0.292	5.7190E-06	2
**Sdhaf1**	1.1057	0.486	0.223	4.0866E-04	2
**Gosr1**	1.1057	0.369	0.167	2.6105E-02	2
**Bace1**	1.1057	0.360	0.164	3.8229E-02	2
**Arhgap18**	1.1047	0.477	0.258	1.5798E-02	2
**Thbs2**	1.1030	0.919	0.578	1.8675E-14	2
**Parp10**	1.1024	0.441	0.218	6.6126E-03	2
**Cox8a**	1.1017	1.000	0.946	6.2287E-25	2
**Plgrkt**	1.1011	0.477	0.233	7.4769E-04	2
**Dusp1**	1.1009	0.613	0.326	2.3773E-05	2
**Sox18**	1.0972	0.477	0.236	1.6719E-03	2
**Adam12**	1.0931	0.414	0.199	1.5463E-02	2
**Ets2**	1.0911	0.910	0.642	9.3748E-15	2
**1700037H04Rik**	1.0895	0.712	0.453	3.7944E-06	2
**Micos13**	1.0894	0.919	0.784	2.7393E-14	2
**Atad3a**	1.0892	0.532	0.243	6.7189E-05	2
**Trip10**	1.0882	0.423	0.211	2.4051E-02	2
**Lum**	1.0868	0.928	0.767	3.6338E-09	2
**Per1**	1.0841	0.441	0.220	1.0992E-02	2
**Acad9**	1.0838	0.450	0.230	1.5980E-02	2
**Whamm**	1.0825	0.387	0.182	2.5663E-02	2
**Spry1**	1.0821	0.396	0.179	8.5217E-03	2
**Col3a1**	1.0816	1.000	0.983	8.3464E-27	2
**Tysnd1**	1.0806	0.685	0.402	1.7584E-05	2
**Xylt2**	1.0785	0.432	0.220	2.7330E-02	2
**Sh3pxd2a**	1.0780	0.883	0.623	1.0579E-12	2
**Bcl6**	1.0776	0.477	0.238	2.5009E-03	2
**Ric1**	1.0769	0.396	0.193	4.1758E-02	2
**Ppif**	1.0765	0.622	0.368	1.1702E-04	2
**Kif1c**	1.0754	0.874	0.556	1.8812E-11	2
**Ptms**	1.0750	1.000	1.000	7.7830E-36	2

Table 1

**Rnf144a**	1.0746	0.441	0.208	5.5142E-03	2
**Dhrs3**	1.0740	0.883	0.590	2.2979E-11	2
**Gnas**	1.0728	1.000	1.000	2.9502E-34	2
**Fos**	1.0726	0.505	0.275	7.7385E-03	2
**Cnst**	1.0726	0.495	0.287	2.7826E-02	2
**Fbn1**	1.0725	0.676	0.368	1.2963E-06	2
**Col4a2**	1.0721	1.000	0.949	2.2291E-22	2
**Gpr180**	1.0719	0.396	0.182	1.2485E-02	2
**Phb**	1.0711	0.450	0.228	2.0671E-02	2
**Pcolce**	1.0704	0.892	0.699	6.2810E-10	2
**Ralgds**	1.0671	0.550	0.265	2.7756E-05	2
**Arhgef11**	1.0671	0.405	0.189	1.4296E-02	2
**Gxylt2**	1.0652	0.568	0.267	2.6966E-05	2
**Tgfbr3**	1.0650	0.784	0.476	6.6423E-09	2
**Sfrp2**	1.0645	0.315	0.123	1.0391E-02	2
**Bicc1**	1.0640	0.541	0.301	3.0655E-03	2
**Slc25a17**	1.0624	0.477	0.240	3.3513E-03	2
**Ghitm**	1.0622	0.883	0.696	5.6935E-09	2
**Fcgrt**	1.0605	0.748	0.564	1.0300E-05	2
**Cmtm4**	1.0602	0.387	0.184	3.3106E-02	2
**Arid5b**	1.0596	0.459	0.226	3.1029E-03	2
**Uqcrb**	1.0592	0.514	0.277	7.1310E-03	2
**Nfix**	1.0591	0.712	0.399	5.2060E-07	2
**Camta2**	1.0575	0.405	0.182	6.7542E-03	2
**Pop5**	1.0567	0.604	0.311	1.9158E-05	2
**Cox7a2**	1.0551	0.901	0.620	1.4789E-11	2
**Rnase4**	1.0525	0.685	0.407	6.3387E-06	2
**Vdac2**	1.0525	0.739	0.502	4.9521E-05	2
**Cdh11**	1.0514	0.703	0.443	2.6343E-05	2
**Inpp5a**	1.0458	0.387	0.174	1.4223E-02	2
**Cdc42ep1**	1.0438	0.694	0.426	3.7583E-06	2
**Ctnnbip1**	1.0431	0.559	0.285	7.1738E-04	2
**Fgd5**	1.0383	0.450	0.211	3.6729E-03	2
**Itgb5**	1.0381	0.739	0.419	2.0714E-07	2
**Letm1**	1.0378	0.748	0.476	2.1141E-06	2
**Gfm1**	1.0352	0.550	0.267	9.0864E-05	2
**Dnajc4**	1.0351	0.523	0.269	2.2480E-03	2
**Tspan4**	1.0349	0.441	0.235	4.7501E-02	2
**Rpe**	1.0339	0.360	0.159	2.6898E-02	2
**Gpam**	1.0336	0.477	0.264	2.3402E-02	2
**Ahnak**	1.0334	0.982	0.878	1.9767E-19	2
**Gys1**	1.0323	0.685	0.394	1.7854E-05	2
**Mfn1**	1.0308	0.523	0.279	2.9788E-03	2
**Capn2**	1.0296	0.757	0.458	1.6898E-06	2

Table 1

**Angptl2**	1.0289	0.892	0.606	1.3164E-09	2
**Mmp2**	1.0282	0.829	0.527	7.5538E-09	2
**Slc25a44**	1.0251	0.568	0.301	4.3559E-04	2
**Atp5mpl**	1.0244	0.730	0.505	4.8498E-05	2
**Dusp3**	1.0240	0.757	0.454	4.0143E-07	2
**Ogn**	1.0238	0.766	0.424	8.6313E-09	2
**Dlk1**	1.0236	0.712	0.361	2.0083E-08	2
**Bpnt1**	1.0219	0.568	0.272	4.5494E-05	2
**Glrx2**	1.0217	0.495	0.238	8.1156E-04	2
**Hspa9**	1.0209	0.973	0.914	8.5212E-17	2
**Gpat4**	1.0176	0.604	0.336	1.7489E-04	2
**Tomm5**	1.0169	0.613	0.378	4.3352E-03	2
**Rgcc**	1.0151	0.955	0.785	2.5040E-15	2
**Tmem147**	1.0149	0.459	0.221	4.3599E-03	2
**Hcfc1r1**	1.0142	0.486	0.231	1.3137E-03	2
**Mgll**	1.0139	0.847	0.672	2.4561E-07	2
**2310039H08Rik**	1.0117	0.559	0.318	2.5562E-03	2
**Ifi27l2a**	1.0111	0.459	0.230	8.7973E-03	2
**Aplp2**	1.0110	0.892	0.652	9.0584E-09	2
**Ltbp2**	1.0102	0.486	0.240	1.7175E-03	2
**Apod**	1.0101	0.577	0.321	1.0591E-03	2
**Timm23**	1.0088	0.829	0.520	2.1355E-09	2
**Micos10**	1.0084	0.550	0.292	1.4127E-03	2
**B3gnt9**	1.0060	0.532	0.301	5.9838E-03	2
**Pwwp2b**	1.0056	0.468	0.228	3.1746E-03	2
**Plxna1**	1.0049	0.468	0.252	3.2724E-02	2
**Isoc1**	1.0000	0.559	0.269	5.2028E-05	2
**Tmem242**	1.0000	0.577	0.285	1.4440E-04	2
**Bnip3**	1.0000	0.495	0.235	7.1678E-04	2
**Plin3**	0.9987	0.739	0.441	3.3200E-07	2
**Ramp2**	0.9976	0.514	0.258	1.3188E-03	2
**Itprid2**	0.9976	0.523	0.280	2.5467E-03	2
**Romo1**	0.9959	0.757	0.525	1.2485E-05	2
**Ankrd9**	0.9936	0.414	0.201	3.0516E-02	2
**Ackr3**	0.9936	0.423	0.211	3.9743E-02	2
**Bag3**	0.9910	0.468	0.211	9.6932E-04	2
**Gtf3c2**	0.9893	0.468	0.253	3.2930E-02	2
**Grpel1**	0.9891	0.874	0.706	2.3169E-09	2
**Dag1**	0.9888	0.523	0.279	4.4805E-03	2
**Ttc28**	0.9876	0.550	0.296	1.1986E-03	2
**Foxred1**	0.9855	0.450	0.228	2.4844E-02	2
**Dgcr6**	0.9846	0.658	0.336	1.5861E-05	2
**Ddhd2**	0.9840	0.432	0.213	2.4972E-02	2
**Aamdc**	0.9826	0.631	0.350	2.1550E-04	2

Table 1

**Hp**	0.9821	0.649	0.417	2.0650E-03	2
**Cald1**	0.9821	0.523	0.299	1.6670E-02	2
**Crispld2**	0.9821	0.477	0.253	2.9159E-02	2
**Lrp6**	0.9804	0.649	0.402	3.1989E-04	2
**Timp1**	0.9804	0.459	0.235	2.7080E-02	2
**Mmp23**	0.9774	0.523	0.285	7.6773E-03	2
**Hspb1**	0.9760	0.595	0.301	1.5056E-04	2
**Pts**	0.9758	0.441	0.226	3.7384E-02	2
**Wwtr1**	0.9745	0.829	0.564	3.4622E-08	2
**Golga3**	0.9745	0.387	0.182	4.6008E-02	2
**Glud1**	0.9737	0.784	0.544	4.8819E-06	2
**Anxa6**	0.9728	0.910	0.635	3.8469E-11	2
**Rxra**	0.9723	0.486	0.265	2.6528E-02	2
**Tomm40l**	0.9717	0.559	0.292	1.4514E-03	2
**Tinagl1**	0.9691	0.568	0.331	8.1588E-03	2
**Lgals1**	0.9685	1.000	0.887	4.9174E-23	2
**Ahdc1**	0.9684	0.468	0.225	5.5864E-03	2
**Jup**	0.9662	0.676	0.409	9.5354E-05	2
**Ifnar2**	0.9660	0.550	0.316	4.2941E-03	2
**Tef**	0.9659	0.784	0.476	6.1408E-07	2
**Dock9**	0.9655	0.586	0.329	9.0930E-04	2
**Pnkd**	0.9651	0.775	0.554	2.4716E-05	2
**Marc2**	0.9639	0.505	0.287	3.1618E-02	2
**Mfap2**	0.9632	0.802	0.466	1.6993E-07	2
**BC029722**	0.9632	0.559	0.299	1.5241E-03	2
**Dusp7**	0.9610	0.532	0.267	2.4388E-03	2
**Itpripl2**	0.9588	0.523	0.299	3.3886E-02	2
**Ak2**	0.9584	0.964	0.872	3.3536E-16	2
**Pard6g**	0.9581	0.640	0.361	4.0305E-04	2
**Atn1**	0.9556	0.396	0.179	1.9498E-02	2
**Six5**	0.9540	0.568	0.287	2.7444E-04	2
**Notch3**	0.9528	0.568	0.329	1.1988E-02	2
**Mgrn1**	0.9515	0.595	0.334	2.5611E-03	2
**Sgce**	0.9494	0.468	0.226	5.8202E-03	2
**Anxa5**	0.9473	0.730	0.417	1.0113E-05	2
**Sh3glb1**	0.9459	0.838	0.627	2.8074E-08	2
**S100a16**	0.9456	0.703	0.470	4.8462E-05	2
**Auh**	0.9452	0.568	0.350	2.7460E-02	2
**Prdx5**	0.9446	0.946	0.742	1.8741E-11	2
**Rgs3**	0.9430	0.550	0.312	9.2744E-03	2
**Ctsf**	0.9427	0.477	0.260	4.9602E-02	2
**Sdhaf4**	0.9420	0.468	0.243	2.9443E-02	2
**Col13a1**	0.9388	0.577	0.329	8.5231E-03	2
**Clpp**	0.9388	0.766	0.471	1.8064E-05	2

Table 1

**Dnlz**	0.9379	0.649	0.399	3.9589E-03	2
**Itgav**	0.9375	0.532	0.299	1.9099E-02	2
**Cthrc1**	0.9370	0.784	0.601	3.7067E-05	2
**Dmac1**	0.9370	0.486	0.257	2.3606E-02	2
**Gpx7**	0.9350	0.514	0.272	1.2229E-02	2
**Gmpr**	0.9324	0.505	0.269	1.8244E-02	2
**Tmem94**	0.9319	0.775	0.525	2.6291E-05	2
**Chkb**	0.9276	0.577	0.348	2.5521E-02	2
**Rgs2**	0.9263	0.739	0.463	2.1237E-05	2
**Rgl1**	0.9262	0.649	0.399	2.6801E-03	2
**Agl**	0.9217	0.459	0.242	4.6734E-02	2
**Nr1d1**	0.9213	0.802	0.478	2.7474E-06	2
**Dab2**	0.9175	0.396	0.186	4.3546E-02	2
**Tspo**	0.9154	0.964	0.919	8.0098E-15	2
**Ddx3x**	0.9135	0.559	0.309	5.1742E-03	2
**Rbms1**	0.9135	0.541	0.323	4.5263E-02	2
**Celf2**	0.9134	0.595	0.338	3.0501E-03	2
**Atp5o**	0.9110	0.847	0.623	2.6414E-07	2
**Dbp**	0.9107	0.577	0.302	7.7665E-04	2
**Arfrp1**	0.9083	0.685	0.355	9.1491E-06	2
**Commd7**	0.9072	0.468	0.243	3.0766E-02	2
**Acot8**	0.9067	0.450	0.226	3.0949E-02	2
**Sptan1**	0.9063	0.784	0.493	7.6465E-07	2
**Smtn**	0.9060	0.640	0.394	3.1491E-03	2
**Twist1**	0.9056	0.604	0.340	1.7967E-03	2
**Klf6**	0.9052	0.622	0.326	3.4984E-04	2
**Ltbp3**	0.9044	0.676	0.427	5.7491E-04	2
**Zfp651**	0.9041	0.541	0.299	9.4550E-03	2
**Arhgef12**	0.9017	0.649	0.390	3.7310E-04	2
**Ndufa13**	0.8994	0.955	0.855	8.3857E-11	2
**Lamp2**	0.8992	0.613	0.387	3.5942E-02	2
**Ptpmt1**	0.8987	0.676	0.399	1.3175E-04	2
**Erbin**	0.8982	0.477	0.230	5.1110E-03	2
**Nbl1**	0.8981	0.964	0.720	5.7501E-13	2
**Dab2ip**	0.8978	0.568	0.292	1.4184E-03	2
**Spag9**	0.8976	0.649	0.360	5.1863E-04	2
**Insr**	0.8952	0.477	0.245	2.2162E-02	2
**Tpst2**	0.8945	0.550	0.311	2.0405E-02	2
**Kdelr3**	0.8925	0.468	0.228	7.8712E-03	2
**Cdk16**	0.8915	0.441	0.203	9.9272E-03	2
**Clec3b**	0.8909	0.928	0.561	1.6886E-13	2
**Dtnbp1**	0.8881	0.667	0.380	4.5605E-04	2
**Add3**	0.8873	0.667	0.405	1.5423E-03	2
**Acta2**	0.8868	0.640	0.397	7.9812E-03	2

Table 1

**BC003965**	0.8845	0.730	0.466	2.6979E-04	2
**Mif4gd**	0.8829	0.577	0.355	2.9153E-02	2
**Zfp395**	0.8795	0.559	0.324	3.1868E-02	2
**Col14a1**	0.8787	0.775	0.410	1.0797E-08	2
**Mmp14**	0.8780	0.811	0.625	8.1986E-04	2
**Cox14**	0.8780	0.595	0.363	2.0573E-02	2
**Sh3glb2**	0.8774	0.712	0.490	3.0782E-03	2
**Ctsd**	0.8737	0.919	0.752	1.0175E-11	2
**Serpinf1**	0.8734	0.964	0.975	5.3886E-10	2
**Eif4ebp1**	0.8707	0.640	0.412	4.9164E-02	2
**Ifitm3**	0.8703	0.640	0.380	6.4130E-03	2
**Rbfox2**	0.8657	0.613	0.297	8.2104E-05	2
**Atp5l**	0.8653	0.784	0.519	2.7897E-05	2
**Chpf**	0.8642	0.667	0.392	6.9768E-04	2
**Rusc2**	0.8640	0.514	0.285	4.4135E-02	2
**Timm50**	0.8612	0.703	0.525	6.4632E-03	2
**Dcn**	0.8609	0.955	0.735	2.4320E-10	2
**Mtln**	0.8578	0.703	0.476	1.9219E-03	2
**Cyhr1**	0.8576	0.748	0.566	4.3580E-04	2
**4931406P16Rik**	0.8568	0.631	0.419	1.7487E-02	2
**Tmem109**	0.8565	0.811	0.593	4.5774E-05	2
**Tnks1bp1**	0.8564	0.631	0.429	4.8892E-02	2
**Cavin1**	0.8556	0.901	0.682	5.4779E-08	2
**Pdlim5**	0.8556	0.595	0.346	1.1773E-02	2
**Tmem184b**	0.8543	0.477	0.236	1.3919E-02	2
**Arhgef17**	0.8508	0.523	0.270	9.7965E-03	2
**Myadm**	0.8500	0.532	0.262	2.2196E-03	2
**Pltp**	0.8491	0.676	0.431	3.0845E-03	2
**Kif1b**	0.8491	0.622	0.340	8.1337E-04	2
**Kxd1**	0.8431	0.640	0.377	1.3274E-03	2
**Qdpr**	0.8394	0.631	0.372	3.3124E-03	2
**Fstl1**	0.8383	0.874	0.605	1.2309E-06	2
**Tkt**	0.8304	1.000	0.997	5.2092E-19	2
**Gorasp2**	0.8301	0.640	0.361	2.0281E-03	2
**Baiap2**	0.8301	0.514	0.272	1.6850E-02	2
**Emp1**	0.8267	0.685	0.459	1.4351E-02	2
**Il11ra1**	0.8251	0.586	0.323	8.2330E-03	2
**Rcn1**	0.8250	0.586	0.353	3.0760E-02	2
**Pdgfb**	0.8240	0.550	0.296	1.3369E-02	2
**Gid4**	0.8230	0.730	0.456	5.7373E-04	2
**Tgoln1**	0.8216	0.685	0.399	1.8839E-03	2
**Boc**	0.8201	0.604	0.328	1.8904E-03	2
**Per3**	0.8198	0.595	0.324	3.5007E-03	2
**Iscu**	0.8194	0.856	0.686	2.8027E-06	2

Table 1

**Foxo1**	0.8169	0.820	0.623	2.6511E-05	2
**Cd93**	0.8164	0.829	0.600	1.6064E-04	2
**Prxl2a**	0.8148	0.955	0.792	5.5390E-09	2
**Galnt2**	0.8115	0.577	0.306	3.4992E-03	2
**Ptp4a1**	0.8112	0.757	0.525	9.4770E-04	2
**Rhot2**	0.8086	0.811	0.559	1.6491E-04	2
**Mgat4b**	0.8079	0.802	0.561	8.6210E-04	2
**Mxd4**	0.8055	0.694	0.453	1.5819E-03	2
**Lix1l**	0.8050	0.694	0.448	3.9522E-03	2
**Smim10l1**	0.7990	0.739	0.475	2.4245E-03	2
**Erlec1**	0.7988	0.568	0.291	2.4950E-03	2
**Cdip1**	0.7959	0.730	0.438	4.3984E-04	2
**Cox17**	0.7893	0.928	0.758	3.9885E-09	2
**Fxyd1**	0.7892	0.982	0.885	1.6620E-10	2
**Nipsnap2**	0.7861	0.703	0.471	4.3970E-02	2
**Htra3**	0.7807	0.883	0.615	5.9552E-06	2
**Man2a2**	0.7803	0.532	0.294	4.1668E-02	2
**Sgta**	0.7801	0.883	0.660	8.0842E-06	2
**Nf2**	0.7781	0.631	0.395	3.1683E-02	2
**Fscn1**	0.7733	0.928	0.691	1.1628E-07	2
**Setbp1**	0.7727	0.514	0.265	2.4363E-02	2
**Hspg2**	0.7723	0.910	0.755	1.0276E-07	2
**Phospho1**	0.7712	0.973	0.922	3.2338E-10	2
**Tgfbi**	0.7703	0.649	0.407	1.2737E-02	2
**Mcam**	0.7693	0.432	0.191	1.5510E-03	2
**Eln**	0.7678	0.982	0.926	3.1086E-08	2
**Cat**	0.7613	0.928	0.816	2.6916E-06	2
**Nrbp2**	0.7612	0.649	0.382	1.1339E-02	2
**Rin2**	0.7601	0.739	0.556	1.5831E-02	2
**Pgam1**	0.7572	0.982	0.875	1.9640E-08	2
**Rab2a**	0.7561	0.676	0.417	7.5945E-03	2
**Plagl1**	0.7553	0.631	0.385	1.7485E-02	2
**Epb41l2**	0.7548	0.721	0.459	3.6046E-03	2
**0610012G03Rik**	0.7546	0.712	0.507	2.0858E-02	2
**Apoe**	0.7546	1.000	0.998	7.3086E-21	2
**Zcchc24**	0.7516	0.730	0.557	3.5878E-03	2
**Antxr1**	0.7514	0.829	0.605	3.3543E-04	2
**Soga1**	0.7465	0.685	0.446	1.1382E-02	2
**Ctdsp1**	0.7449	0.811	0.578	1.7534E-03	2
**Reep5**	0.7299	1.000	0.973	4.3161E-15	2
**Gas6**	0.7286	0.811	0.552	2.2163E-04	2
**Mprip**	0.7284	0.847	0.677	4.7073E-04	2
**Lrg1**	0.7177	0.820	0.547	7.9470E-04	2
**Scarf2**	0.7173	0.838	0.610	1.4854E-04	2

Table 1

**1110008P14Rik**	0.7164	0.712	0.422	3.3086E-03	2
**Igfbp7**	0.7138	1.000	0.975	2.4120E-16	2
**Crk**	0.7134	0.829	0.576	5.8283E-04	2
**Angptl1**	0.7131	0.514	0.264	2.2986E-02	2
**Ntn1**	0.7127	0.613	0.367	1.6911E-02	2
**Adamts2**	0.7119	0.811	0.611	1.0870E-02	2
**Islr**	0.7107	0.955	0.760	1.0760E-06	2
**Ppa1**	0.7105	0.919	0.772	1.9525E-05	2
**Zcchc14**	0.7080	0.802	0.579	3.0591E-03	2
**Chp1**	0.7076	0.838	0.627	4.2047E-03	2
**Nedd4**	0.7021	0.865	0.735	1.9287E-03	2
**Ywhag**	0.7002	0.991	0.931	1.2791E-12	2
**Zfp91**	0.6945	0.694	0.421	5.3473E-03	2
**Map4k4**	0.6917	0.748	0.556	3.5768E-02	2
**Rsrp1**	0.6896	1.000	0.959	8.1590E-12	2
**Phb2**	0.6867	0.847	0.698	4.0170E-04	2
**Ankrd40**	0.6861	0.649	0.390	4.3951E-02	2
**Cdkn1c**	0.6787	0.865	0.657	1.0265E-04	2
**Hsbp1**	0.6773	0.838	0.581	2.8681E-03	2
**Dkk3**	0.6760	0.676	0.389	6.0775E-03	2
**Id1**	0.6758	0.901	0.726	2.8614E-05	2
**Id3**	0.6728	0.928	0.865	1.3677E-05	2
**Nenf**	0.6723	0.883	0.671	2.7762E-04	2
**Fam3c**	0.6700	0.802	0.596	1.3539E-02	2
**Ndufa2**	0.6671	0.865	0.796	3.9662E-03	2
**2700081O15Rik**	0.6629	0.838	0.645	7.3044E-03	2
**Anxa2**	0.6616	0.964	0.895	7.9266E-08	2
**Pmepa1**	0.6607	0.820	0.603	5.5039E-03	2
**Capns1**	0.6599	0.973	0.916	4.6687E-09	2
**Col11a1**	0.6592	0.865	0.791	1.1615E-03	2
**Plxnd1**	0.6562	0.892	0.740	4.4980E-04	2
**Txn2**	0.6512	0.910	0.753	1.8766E-03	2
**Tomm40**	0.6505	0.874	0.611	1.2316E-03	2
**Plpp3**	0.6475	0.973	0.845	1.3766E-07	2
**Prdx6**	0.6460	0.802	0.617	2.8123E-02	2
**Pamr1**	0.6457	0.613	0.356	3.2918E-02	2
**Bcap31**	0.6434	0.865	0.742	1.2276E-03	2
**Tcn2**	0.6432	0.766	0.485	3.6531E-03	2
**Lamp1**	0.6431	0.901	0.752	2.1989E-05	2
**Mknk2**	0.6424	0.775	0.542	8.2740E-03	2
**Nrp2**	0.6401	0.928	0.748	1.6288E-04	2
**Slc25a4**	0.6391	1.000	0.986	8.9062E-12	2
**Fmod**	0.6342	0.910	0.720	1.7429E-04	2
**Atp5e**	0.6339	1.000	0.998	2.4109E-14	2

Table 1

**Col16a1**	0.6335	0.973	0.912	8.5453E-10	2
**Qk**	0.6270	0.883	0.716	2.5561E-04	2
**Nfe2l1**	0.6246	0.910	0.742	1.2577E-03	2
**Reep3**	0.6180	0.793	0.603	1.5684E-02	2
**Tle5**	0.6137	0.991	0.971	1.1352E-10	2
**Lrrc58**	0.6108	0.829	0.655	8.8057E-03	2
**Timm13**	0.6036	0.883	0.758	4.8870E-02	2
**Ubc**	0.6009	0.982	0.961	3.2468E-08	2
**Marcks**	0.5960	0.982	0.919	1.4930E-08	2
**Slc25a5**	0.5948	0.982	0.870	6.0916E-04	2
**Ctsl**	0.5927	0.910	0.836	2.7087E-04	2
**Hspd1**	0.5880	0.901	0.750	1.4925E-02	2
**Col6a2**	0.5854	1.000	0.912	4.7696E-10	2
**Gpi1**	0.5821	0.991	0.993	3.6791E-11	2
**Pkm**	0.5796	1.000	0.981	1.2007E-09	2
**Col1a1**	0.5792	1.000	1.000	2.6649E-07	2
**S100a6**	0.5713	0.955	0.836	2.6397E-04	2
**Myo1c**	0.5712	0.982	0.887	1.7776E-06	2
**Dynll2**	0.5686	0.856	0.718	3.9868E-03	2
**Selenop**	0.5685	0.946	0.845	1.5010E-04	2
**Gsn**	0.5675	0.982	0.921	2.3629E-06	2
**Cdkn1a**	0.5670	0.883	0.672	1.1742E-02	2
**Col5a1**	0.5670	1.000	0.998	2.9658E-10	2
**Nfic**	0.5638	0.829	0.608	3.7575E-02	2
**Arf4**	0.5589	0.919	0.814	7.6882E-03	2
**Ndufb7**	0.5526	0.991	0.976	1.4422E-08	2
**Col5a2**	0.5518	1.000	0.997	7.9617E-09	2
**Tcf4**	0.5476	0.937	0.828	1.4970E-03	2
**Mtch1**	0.5417	0.982	0.927	1.9122E-06	2
**Psap**	0.5377	1.000	1.000	6.9663E-11	2
**Ctsz**	0.5329	0.883	0.726	3.9303E-02	2
**Tgfbr2**	0.5322	0.892	0.743	3.0754E-02	2
**Mapkapk2**	0.5311	0.883	0.775	3.3426E-02	2
**Lox**	0.5299	0.856	0.713	4.4660E-02	2
**Plec**	0.5278	0.919	0.867	1.8475E-02	2
**Slc1a5**	0.5193	0.991	0.892	3.9134E-03	2
**Ube2m**	0.5095	0.964	0.894	3.4717E-03	2
**Stfa3**	1.5423	0.301	0.112	3.8623E-03	3
**Usp1**	1.2825	0.330	0.128	4.7357E-03	3
**Hist3h2ba**	1.0728	0.427	0.178	8.3508E-04	3
**Xcl1**	2.6433	0.113	0.020	2.5284E-02	4
**Dclk2**	2.2807	0.134	0.028	2.8997E-02	4
**Prokr1**	2.2807	0.134	0.028	2.8997E-02	4
**Oas2**	2.0583	0.216	0.053	2.5604E-04	4

Table 1

**Nat2**	1.8932	0.196	0.053	8.0409E-03	4
**Tex9**	1.8657	0.196	0.053	8.8208E-03	4
**Unc5cl**	1.8173	0.196	0.058	3.6198E-02	4
**Fanca**	1.7885	0.381	0.124	9.9711E-07	4
**Spic**	1.7877	0.196	0.058	3.7817E-02	4
**Kif4**	1.7502	0.216	0.069	3.7586E-02	4
**Malt1**	1.7227	0.268	0.086	1.7385E-03	4
**Lin28a**	1.6433	0.299	0.106	2.1298E-03	4
**Tonsl**	1.6433	0.278	0.096	4.2903E-03	4
**Gpr141**	1.6433	0.206	0.061	2.4601E-02	4
**Tnpo2**	1.6433	0.237	0.079	2.7554E-02	4
**4930404N11Rik**	1.6186	0.289	0.091	4.6457E-04	4
**Eif2ak2**	1.5867	0.237	0.076	1.5631E-02	4
**Tbxas1**	1.5859	0.454	0.206	3.5032E-04	4
**Cdca7l**	1.5805	0.381	0.137	3.1943E-05	4
**Mcemp1**	1.5777	0.629	0.244	9.9349E-12	4
**Rtel1**	1.5635	0.381	0.135	3.7029E-05	4
**Cenpu**	1.5475	0.423	0.147	2.0316E-06	4
**Olfm4**	1.5475	0.412	0.157	3.2334E-05	4
**Fignl1**	1.5213	0.309	0.112	2.9311E-03	4
**Tacc3**	1.5199	0.464	0.175	9.2964E-07	4
**Blm**	1.5177	0.330	0.142	2.4171E-02	4
**Galns**	1.4982	0.454	0.172	2.5021E-06	4
**Armt1**	1.4944	0.227	0.074	4.5511E-02	4
**Rad54l**	1.4709	0.495	0.198	1.0839E-06	4
**Ube2c**	1.4706	0.495	0.185	1.3348E-07	4
**Ikzf3**	1.4706	0.423	0.186	6.9123E-04	4
**Rinl**	1.4579	0.515	0.193	9.4996E-08	4
**Trmo**	1.4526	0.371	0.155	2.9174E-03	4
**Haus5**	1.4469	0.371	0.152	1.9294E-03	4
**Itgam**	1.4465	0.485	0.211	2.3044E-05	4
**Iqgap3**	1.4427	0.454	0.188	5.0696E-05	4
**Trub2**	1.4416	0.371	0.139	2.5140E-04	4
**Gmeb2**	1.4368	0.330	0.134	1.3556E-02	4
**Fhdc1**	1.4331	0.701	0.335	1.9652E-10	4
**Wrn**	1.4327	0.299	0.117	3.3071E-02	4
**Igll1**	1.4268	0.701	0.287	1.3531E-11	4
**Otub2**	1.4165	0.423	0.149	5.2730E-06	4
**Eri1**	1.4155	0.330	0.127	5.2655E-03	4
**Cyb561a3**	1.4130	0.381	0.168	6.2620E-03	4
**Gba2**	1.4098	0.340	0.132	3.6341E-03	4
**Ncapg**	1.4088	0.464	0.168	7.7653E-07	4
**Pon2**	1.4070	0.433	0.200	1.4583E-03	4
**Traf3ip3**	1.4062	0.474	0.177	1.1021E-06	4

Table 1

**Cd300a**	1.4034	0.402	0.158	2.7435E-04	4
**Pidd1**	1.4001	0.598	0.249	1.8837E-08	4
**Pdia2**	1.3876	0.392	0.170	3.1225E-03	4
**Pik3cg**	1.3825	0.423	0.170	1.6520E-04	4
**Btk**	1.3802	0.320	0.120	6.6453E-03	4
**Cdkn3**	1.3770	0.330	0.130	8.5724E-03	4
**Igsf6**	1.3767	0.474	0.215	1.7170E-04	4
**Pax5**	1.3765	0.505	0.201	1.3101E-06	4
**Rasgrp1**	1.3732	0.320	0.114	2.2085E-03	4
**H2-DMb2**	1.3732	0.309	0.124	3.1995E-02	4
**Foxm1**	1.3702	0.320	0.125	1.5607E-02	4
**Bloc1s3**	1.3632	0.351	0.150	1.6878E-02	4
**Nfe2**	1.3538	0.732	0.353	6.7513E-10	4
**Rbm43**	1.3538	0.340	0.130	3.4385E-03	4
**Cenpk**	1.3529	0.546	0.234	1.3570E-06	4
**Aurkb**	1.3478	0.546	0.271	3.3653E-05	4
**Arid3a**	1.3461	0.629	0.276	2.9334E-08	4
**Pole2**	1.3356	0.505	0.208	6.1663E-06	4
**Ly6d**	1.3356	0.454	0.190	1.2938E-04	4
**Ccr2**	1.3351	0.361	0.145	4.1072E-03	4
**Jpt1**	1.3347	0.526	0.226	4.2560E-06	4
**Mthfd1**	1.3294	0.546	0.248	5.8113E-06	4
**6030468B19Rik**	1.3213	0.639	0.281	3.0399E-08	4
**Svip**	1.3161	0.464	0.190	3.4441E-05	4
**Abca7**	1.3149	0.402	0.160	5.7357E-04	4
**1700123O20Rik**	1.3140	0.330	0.132	1.7863E-02	4
**Fcnb**	1.3133	0.629	0.312	1.4213E-06	4
**Cand1**	1.3121	0.299	0.112	2.0836E-02	4
**Mzb1**	1.3119	0.660	0.277	1.4103E-09	4
**Xpo7**	1.3085	0.835	0.394	8.4825E-13	4
**Helb**	1.3067	0.454	0.205	4.4464E-04	4
**Gtse1**	1.3048	0.567	0.279	1.5475E-05	4
**Chtf18**	1.3013	0.412	0.147	2.3016E-05	4
**Dusp2**	1.2979	0.495	0.206	1.2876E-05	4
**Kif21b**	1.2924	0.495	0.233	1.7484E-04	4
**Dntt**	1.2886	0.722	0.348	1.2712E-09	4
**Zfp131**	1.2871	0.433	0.183	4.5875E-04	4
**Oas3**	1.2864	0.639	0.315	4.0131E-07	4
**Pop7**	1.2837	0.402	0.190	2.7117E-02	4
**Iglc1**	1.2831	0.505	0.223	3.7522E-05	4
**Ftsj1**	1.2765	0.351	0.139	6.1775E-03	4
**Dhfr**	1.2698	0.392	0.170	5.0900E-03	4
**Snrpd1**	1.2689	0.598	0.274	1.1380E-06	4
**Adpgk**	1.2689	0.433	0.193	1.5481E-03	4

Table 1

**Nop58**	1.2678	0.742	0.462	4.3107E-08	4
**Sowaha**	1.2661	0.742	0.417	4.0939E-08	4
**Hmox1**	1.2661	0.629	0.252	6.5489E-09	4
**Trpv2**	1.2631	0.526	0.231	2.1061E-05	4
**Gmfg**	1.2542	0.371	0.158	1.0945E-02	4
**Ube2t**	1.2509	0.464	0.203	4.7495E-04	4
**B9d2**	1.2487	0.485	0.215	2.9781E-04	4
**E2f1**	1.2484	0.588	0.281	4.2668E-06	4
**E2f7**	1.2467	0.443	0.170	4.7708E-05	4
**Adar**	1.2467	0.412	0.178	2.1490E-03	4
**Dock2**	1.2459	0.742	0.363	5.2064E-10	4
**Arhgap9**	1.2447	0.464	0.191	7.9272E-05	4
**Arhgap11a**	1.2439	0.464	0.213	8.0553E-04	4
**Ammecr1**	1.2389	0.557	0.243	4.9279E-06	4
**Ckap2**	1.2375	0.639	0.312	7.1554E-07	4
**Tal1**	1.2373	0.763	0.409	3.7648E-09	4
**Mzt1**	1.2366	0.392	0.162	2.6381E-03	4
**Top2a**	1.2352	0.732	0.389	1.0103E-08	4
**Rasal3**	1.2282	0.299	0.116	3.8221E-02	4
**Ptpn6**	1.2262	0.588	0.290	2.0487E-05	4
**Cd5l**	1.2243	0.402	0.172	3.3085E-03	4
**Trim12c**	1.2240	0.392	0.167	4.6981E-03	4
**Pik3ap1**	1.2232	0.546	0.248	2.3469E-05	4
**Wdr74**	1.2206	0.381	0.175	4.0591E-02	4
**Plcl2**	1.2198	0.732	0.351	4.0726E-10	4
**E2f3**	1.2197	0.505	0.229	1.5983E-04	4
**Nfatc2ip**	1.2144	0.454	0.198	6.1757E-04	4
**Ankle1**	1.2133	0.722	0.350	2.5206E-09	4
**Kras**	1.2133	0.330	0.137	4.5405E-02	4
**Bub1b**	1.2126	0.433	0.172	2.1196E-04	4
**Kif11**	1.2100	0.876	0.474	6.4343E-13	4
**Akna**	1.2064	0.753	0.371	3.6718E-09	4
**Parp9**	1.2059	0.392	0.183	3.7134E-02	4
**Ltb4r1**	1.1985	0.464	0.191	1.1548E-04	4
**Smc2**	1.1967	0.670	0.340	5.6700E-07	4
**Ms4a1**	1.1937	0.515	0.236	1.9733E-04	4
**Dcp2**	1.1932	0.423	0.190	6.0800E-03	4
**Iba57**	1.1895	0.505	0.254	1.9185E-03	4
**Ints7**	1.1887	0.608	0.295	3.1869E-06	4
**Epb42**	1.1884	0.443	0.215	7.0504E-03	4
**Lamtor5**	1.1866	0.402	0.180	1.1005E-02	4
**Snx22**	1.1838	0.526	0.257	7.3031E-04	4
**Fcna**	1.1838	0.443	0.226	2.2090E-02	4
**Snx20**	1.1823	0.588	0.295	2.5196E-05	4

Table 1

**Mcm10**	1.1773	0.577	0.279	4.9280E-05	4
**Topbp1**	1.1732	0.577	0.264	7.7805E-06	4
**Uba1**	1.1726	0.588	0.259	1.5233E-06	4
**Sash3**	1.1716	0.505	0.264	2.6375E-03	4
**Mafk**	1.1710	0.691	0.353	1.3063E-07	4
**Dkc1**	1.1710	0.639	0.355	6.0466E-06	4
**Gpr155**	1.1708	0.361	0.152	2.3999E-02	4
**Clasrp**	1.1703	0.454	0.221	6.0822E-03	4
**Pld4**	1.1693	0.670	0.315	1.6778E-07	4
**Rad18**	1.1682	0.433	0.193	3.5152E-03	4
**Zfp39**	1.1679	0.381	0.160	1.0739E-02	4
**Kif18a**	1.1668	0.412	0.177	3.6444E-03	4
**Cdca2**	1.1633	0.351	0.147	2.5956E-02	4
**Ptpn7**	1.1629	0.577	0.266	1.8894E-05	4
**Acap1**	1.1614	0.443	0.218	1.9321E-02	4
**Ltf**	1.1595	1.000	0.917	1.9292E-21	4
**Apol8**	1.1593	0.515	0.261	1.6150E-03	4
**Fermt3**	1.1568	0.856	0.546	1.6935E-09	4
**Gmip**	1.1539	0.536	0.279	1.6025E-03	4
**Cited4**	1.1523	0.711	0.337	3.8981E-08	4
**Diaph3**	1.1514	0.567	0.276	2.2860E-05	4
**Tmc8**	1.1510	0.577	0.277	3.0242E-05	4
**Rfwd3**	1.1510	0.546	0.284	5.6773E-04	4
**Cybc1**	1.1502	0.423	0.200	2.3227E-02	4
**Aldh3b1**	1.1500	0.639	0.314	9.4404E-07	4
**Nfam1**	1.1477	0.485	0.243	6.0110E-03	4
**Smg6**	1.1452	0.443	0.208	4.2160E-03	4
**Nmral1**	1.1426	0.619	0.335	1.0721E-04	4
**Slfn14**	1.1399	0.680	0.309	9.1957E-08	4
**Elovl1**	1.1397	0.608	0.294	2.5089E-05	4
**1810009A15Rik**	1.1396	0.505	0.262	1.1956E-02	4
**Eed**	1.1392	0.433	0.210	2.7596E-02	4
**Atad5**	1.1358	0.412	0.198	4.5468E-02	4
**Myb**	1.1336	0.887	0.606	1.4429E-10	4
**Timm17b**	1.1326	0.536	0.274	1.5885E-03	4
**Ska1**	1.1326	0.608	0.322	3.4235E-05	4
**Psd4**	1.1323	0.722	0.398	5.9031E-07	4
**Ccdc125**	1.1323	0.567	0.276	1.2905E-04	4
**Sell**	1.1323	0.485	0.211	2.6603E-04	4
**Ppil1**	1.1323	0.443	0.218	2.0159E-02	4
**Slc7a5**	1.1287	0.619	0.318	1.9430E-05	4
**Stard10**	1.1287	0.804	0.490	8.4631E-09	4
**Slc14a1**	1.1287	0.670	0.360	1.9979E-05	4
**Sept1**	1.1272	0.649	0.333	9.2647E-06	4

Table 1

**Zfp367**	1.1271	0.639	0.340	8.2972E-06	4
**Ifrd2**	1.1267	0.804	0.437	4.4434E-09	4
**Rcc1**	1.1254	0.505	0.257	2.2198E-03	4
**Rad51**	1.1245	0.443	0.211	1.1208E-02	4
**Arhgap4**	1.1228	0.474	0.239	6.8308E-03	4
**Med8**	1.1220	0.454	0.210	4.4802E-03	4
**Dlgap5**	1.1205	0.577	0.277	5.5256E-05	4
**Arrdc1**	1.1189	0.588	0.300	2.3557E-04	4
**Cd79b**	1.1183	0.887	0.569	2.4764E-11	4
**Dna2**	1.1094	0.515	0.239	5.7976E-04	4
**Nfkbib**	1.1085	0.577	0.281	7.7007E-05	4
**Wdr76**	1.1083	0.629	0.300	1.6861E-05	4
**Mylk3**	1.1060	0.577	0.285	1.4008E-04	4
**Lrmp**	1.1035	0.814	0.459	2.0720E-09	4
**Fam49b**	1.1021	0.567	0.287	6.8971E-04	4
**Chaf1a**	1.1011	0.691	0.408	2.5692E-05	4
**Slc16a6**	1.0996	0.454	0.228	3.2886E-02	4
**Creg1**	1.0986	0.660	0.380	3.6967E-05	4
**Pum2**	1.0971	0.629	0.312	2.8791E-05	4
**Nadk2**	1.0970	0.753	0.406	3.6635E-07	4
**Tinf2**	1.0966	0.608	0.310	9.5744E-05	4
**Lcn2**	1.0953	0.990	0.868	4.6711E-20	4
**Orc2**	1.0944	0.598	0.289	5.6184E-05	4
**Cenph**	1.0927	0.722	0.340	9.4546E-08	4
**Aunip**	1.0902	0.495	0.213	3.9531E-04	4
**Mybl2**	1.0892	0.722	0.394	8.7027E-07	4
**Rlim**	1.0887	0.433	0.195	6.9572E-03	4
**Tspo2**	1.0883	0.856	0.492	1.0944E-09	4
**Gcnt1**	1.0873	0.505	0.259	4.9724E-03	4
**Rhno1**	1.0873	0.495	0.256	7.8633E-03	4
**S100a8**	1.0873	1.000	0.995	2.1847E-30	4
**Madd**	1.0835	0.577	0.284	1.1405E-04	4
**Itga4**	1.0832	0.536	0.290	1.0876E-02	4
**Elane**	1.0831	0.948	0.733	1.1754E-11	4
**Kif15**	1.0810	0.485	0.251	2.5636E-02	4
**Hist1h3a**	1.0803	0.495	0.264	3.7461E-02	4
**Snap29**	1.0800	0.546	0.249	1.0476E-04	4
**St3gal5**	1.0794	0.608	0.315	1.7362E-04	4
**Slc16a10**	1.0791	0.804	0.441	2.9489E-09	4
**Cdca4**	1.0782	0.907	0.620	4.0216E-11	4
**H2-T24**	1.0779	0.557	0.266	3.8534E-04	4
**Parpbp**	1.0773	0.423	0.203	3.6675E-02	4
**Ptprcap**	1.0768	0.928	0.635	2.8562E-11	4
**Cdc6**	1.0759	0.887	0.635	8.6195E-10	4

Table 1

**Fam126a**	1.0756	0.474	0.218	2.8492E-03	4
**Ddx39**	1.0747	0.794	0.477	7.8563E-09	4
**Exosc7**	1.0725	0.495	0.254	1.4816E-02	4
**Lsm8**	1.0721	0.701	0.365	1.6254E-06	4
**Tspan33**	1.0706	0.722	0.404	3.7553E-06	4
**Atad2**	1.0704	0.701	0.414	3.1284E-05	4
**Fam110a**	1.0681	0.423	0.201	2.3789E-02	4
**Stag2**	1.0675	0.649	0.366	3.5520E-04	4
**Drg1**	1.0674	0.433	0.216	4.8136E-02	4
**Irf5**	1.0672	0.464	0.221	8.6216E-03	4
**Gna15**	1.0669	0.464	0.226	1.0457E-02	4
**Zc3h15**	1.0665	0.515	0.226	2.5815E-04	4
**Aldh1a1**	1.0657	0.526	0.241	4.4239E-04	4
**Nfya**	1.0638	0.608	0.347	1.8625E-03	4
**Vcam1**	1.0629	0.701	0.348	6.3343E-07	4
**AI467606**	1.0627	0.722	0.363	1.5708E-07	4
**Hmgb3**	1.0622	0.784	0.401	1.5530E-08	4
**Psmf1**	1.0583	0.722	0.411	8.5756E-06	4
**B4galnt1**	1.0583	0.588	0.285	1.1100E-04	4
**Cactin**	1.0583	0.557	0.307	6.8292E-03	4
**Sbno1**	1.0583	0.423	0.193	1.5102E-02	4
**Asns**	1.0550	0.784	0.480	3.9167E-06	4
**Tmem123**	1.0543	0.722	0.403	9.2074E-07	4
**Cdca8**	1.0540	0.897	0.601	1.3514E-11	4
**Nrros**	1.0522	0.567	0.315	1.1022E-02	4
**Me2**	1.0518	0.577	0.290	4.0259E-04	4
**Tpx2**	1.0516	0.536	0.277	4.2854E-03	4
**Sbf1**	1.0514	0.515	0.279	9.0908E-03	4
**Blnk**	1.0512	0.505	0.251	4.2581E-03	4
**Itgb2**	1.0506	0.856	0.545	1.1686E-07	4
**Oip5**	1.0504	0.485	0.246	2.3620E-02	4
**Birc5**	1.0493	0.979	0.810	7.5529E-17	4
**Trim58**	1.0482	0.619	0.347	4.4280E-04	4
**Stxbp2**	1.0454	0.722	0.394	7.1142E-06	4
**Cep76**	1.0452	0.515	0.282	3.4920E-02	4
**Cd52**	1.0452	0.928	0.645	3.0964E-11	4
**Cd19**	1.0440	0.742	0.450	2.9743E-06	4
**Nudt21**	1.0437	0.722	0.436	1.1037E-05	4
**Pklr**	1.0436	0.732	0.424	2.4539E-06	4
**Blvrb**	1.0434	1.000	0.997	9.4333E-35	4
**Zfpm1**	1.0429	0.763	0.530	6.0463E-05	4
**Espl1**	1.0422	0.608	0.314	3.4113E-04	4
**Nasp**	1.0416	0.619	0.338	5.6332E-04	4
**Gpn2**	1.0409	0.454	0.223	2.2290E-02	4

Table 1

**Rnf20**	1.0406	0.649	0.290	1.2808E-06	4
**Cdc45**	1.0380	0.608	0.340	3.9794E-04	4
**Gfi1b**	1.0364	0.742	0.436	2.8899E-06	4
**Ak6**	1.0353	0.598	0.318	4.2289E-04	4
**Nrf1**	1.0351	0.660	0.375	3.6467E-04	4
**Dyrk1a**	1.0346	0.691	0.413	6.7016E-05	4
**Tcf19**	1.0341	0.691	0.416	1.3637E-04	4
**Add1**	1.0338	0.722	0.409	6.5134E-06	4
**Kel**	1.0337	0.794	0.474	1.6018E-07	4
**Nt5c3**	1.0336	0.670	0.383	1.0510E-04	4
**Kif22**	1.0326	0.773	0.432	1.1447E-07	4
**H2-T23**	1.0321	0.660	0.376	1.9632E-04	4
**Fmr1**	1.0294	0.485	0.259	2.8212E-02	4
**Syce2**	1.0289	0.567	0.307	2.2274E-03	4
**Prkcb**	1.0255	0.474	0.239	1.6890E-02	4
**Trim56**	1.0244	0.680	0.366	2.0974E-05	4
**Stx11**	1.0240	0.619	0.355	4.3400E-04	4
**Hpf1**	1.0238	0.680	0.431	2.4302E-04	4
**Mcm5**	1.0215	0.938	0.688	1.8246E-10	4
**Ptprj**	1.0214	0.629	0.342	5.9850E-04	4
**Prtn3**	1.0206	0.979	0.726	8.2447E-12	4
**Nup133**	1.0197	0.608	0.335	7.5894E-04	4
**Yod1**	1.0188	0.608	0.320	9.5441E-04	4
**Pmf1**	1.0184	0.722	0.368	2.1556E-06	4
**Sae1**	1.0174	0.691	0.411	2.8247E-05	4
**Gtpbp1**	1.0167	0.897	0.647	2.7262E-09	4
**Bicra**	1.0157	0.608	0.342	1.6144E-03	4
**Plekha2**	1.0152	0.619	0.335	2.6353E-04	4
**Ppp1r35**	1.0147	0.536	0.305	3.7725E-02	4
**A630001G21Rik**	1.0132	0.526	0.236	2.5029E-04	4
**Ccm2**	1.0132	0.598	0.317	1.5109E-03	4
**Slc38a5**	1.0130	0.660	0.347	1.5431E-04	4
**Parvg**	1.0123	0.670	0.376	6.5630E-05	4
**Trim10**	1.0113	0.959	0.752	1.5266E-12	4
**E2f2**	1.0094	0.701	0.394	6.7875E-05	4
**Timm22**	1.0094	0.495	0.238	5.8883E-03	4
**Gclm**	1.0082	0.948	0.726	4.3704E-13	4
**Inpp5d**	1.0074	0.722	0.450	2.2691E-05	4
**Camp**	1.0070	1.000	0.995	2.6179E-26	4
**Ibtk**	1.0058	0.588	0.348	1.0707E-02	4
**Eloa**	1.0058	0.505	0.266	2.0817E-02	4
**Ncapd2**	1.0047	0.959	0.734	1.5007E-13	4
**Lta4h**	1.0034	0.732	0.488	1.4787E-04	4
**Mkrn1**	0.9997	0.979	0.875	3.2585E-18	4

Table 1

**Adgrg1**	0.9980	0.794	0.427	7.2786E-07	4
**Ctsg**	0.9971	0.701	0.388	3.5671E-05	4
**Gins2**	0.9962	0.608	0.340	4.2483E-03	4
**Kmt2b**	0.9961	0.464	0.228	2.1100E-02	4
**Zc3hav1**	0.9952	0.660	0.348	5.7361E-05	4
**Rpain**	0.9951	0.515	0.269	1.5916E-02	4
**Imp3**	0.9942	0.505	0.266	2.3519E-02	4
**Ighj1**	0.9940	0.938	0.583	3.3333E-10	4
**Klf1**	0.9938	0.598	0.358	1.0467E-02	4
**Sptb**	0.9908	1.000	0.866	6.8612E-20	4
**Stmn1**	0.9898	0.907	0.574	6.9163E-09	4
**Pafah1b3**	0.9897	0.794	0.460	5.8846E-07	4
**Arl6ip4**	0.9893	0.814	0.474	1.0808E-07	4
**Car2**	0.9888	1.000	0.997	1.2714E-31	4
**Kdm3b**	0.9876	0.577	0.304	1.7636E-03	4
**Mpeg1**	0.9861	0.588	0.297	3.0049E-04	4
**Alas2**	0.9843	1.000	0.901	9.5222E-19	4
**Hist1h4f**	0.9837	0.990	0.812	2.6625E-16	4
**Fam241a**	0.9831	0.784	0.460	4.1100E-06	4
**Btrc**	0.9784	0.588	0.330	3.6704E-03	4
**Abtb1**	0.9756	0.742	0.419	1.1166E-05	4
**Mcm4**	0.9756	0.907	0.620	4.6062E-10	4
**Ncbp1**	0.9742	0.629	0.361	6.7902E-03	4
**Rab3il1**	0.9731	0.887	0.663	3.0127E-08	4
**Spn**	0.9723	0.814	0.485	4.0239E-07	4
**Rab44**	0.9712	0.536	0.279	4.8375E-03	4
**Dnajb1**	0.9708	0.814	0.507	4.2313E-06	4
**Plekhj1**	0.9690	0.639	0.366	6.7705E-04	4
**Dok3**	0.9690	0.866	0.649	4.8378E-06	4
**Gadd45a**	0.9684	0.804	0.525	2.1641E-05	4
**Zbtb22**	0.9674	0.557	0.327	3.2709E-02	4
**Spta1**	0.9668	0.979	0.904	1.4684E-18	4
**Trp53**	0.9666	0.753	0.447	3.5588E-05	4
**Hk3**	0.9665	0.567	0.271	6.7746E-04	4
**Stk4**	0.9643	0.680	0.370	1.1213E-04	4
**Mpo**	0.9641	0.773	0.512	7.5372E-05	4
**Ngp**	0.9640	1.000	0.969	1.1776E-19	4
**Tmem131l**	0.9639	0.557	0.299	8.9671E-03	4
**Irf8**	0.9634	0.608	0.368	1.0614E-02	4
**Ep300**	0.9628	0.691	0.427	3.8563E-04	4
**Lsm7**	0.9627	0.557	0.305	1.5705E-02	4
**Ccnd3**	0.9622	0.876	0.627	1.5270E-06	4
**Dhrs11**	0.9616	0.660	0.363	1.3854E-04	4
**Mad2l1**	0.9591	0.598	0.333	4.3404E-03	4

Table 1

**Dbnl**	0.9569	0.701	0.421	7.0427E-04	4
**Snx15**	0.9568	0.742	0.446	7.0588E-05	4
**Slc25a37**	0.9563	1.000	0.949	5.5052E-23	4
**Fgd3**	0.9559	0.598	0.309	1.5860E-03	4
**Psmb8**	0.9546	0.598	0.340	1.3320E-02	4
**Tipin**	0.9542	0.804	0.525	4.5131E-06	4
**Smox**	0.9540	0.814	0.540	2.1919E-05	4
**Cenpn**	0.9522	0.588	0.317	3.4941E-03	4
**Thoc3**	0.9521	0.454	0.211	1.5661E-02	4
**Mcm6**	0.9514	0.918	0.587	5.7283E-09	4
**Hist4h4**	0.9483	1.000	0.954	8.9267E-24	4
**Unc93b1**	0.9475	0.536	0.299	2.9885E-02	4
**Dek**	0.9474	1.000	0.939	2.8060E-21	4
**Csf3r**	0.9460	0.536	0.282	1.0851E-02	4
**Sgo1**	0.9454	0.577	0.315	8.3203E-03	4
**Dck**	0.9452	0.876	0.566	4.1298E-08	4
**Ncf4**	0.9450	0.691	0.355	1.8515E-05	4
**Hbb-bt**	0.9448	1.000	0.998	2.5399E-30	4
**Bop1**	0.9440	0.629	0.368	2.1938E-03	4
**Tlnrd1**	0.9437	0.701	0.465	6.6772E-04	4
**Rnps1**	0.9428	0.567	0.328	2.1559E-02	4
**Cxcr4**	0.9423	0.907	0.653	5.5981E-08	4
**Hist2h2bb**	0.9418	1.000	1.000	1.1994E-36	4
**Gpcpd1**	0.9418	0.649	0.401	2.1480E-03	4
**Cdk6**	0.9417	0.608	0.384	2.0720E-02	4
**Dyrk3**	0.9412	0.546	0.279	5.5535E-03	4
**2310009A05Rik**	0.9407	0.680	0.403	3.1392E-04	4
**Ctse**	0.9394	1.000	0.946	4.0040E-21	4
**Rad21**	0.9370	0.608	0.365	1.1845E-02	4
**Srgn**	0.9368	0.753	0.460	4.7460E-05	4
**Kif14**	0.9359	0.515	0.267	1.9792E-02	4
**Azin1**	0.9354	0.619	0.338	1.7656E-03	4
**Slc43a1**	0.9347	0.608	0.312	2.0062E-03	4
**Pcnt**	0.9335	0.670	0.409	1.1133E-03	4
**Tfdp2**	0.9332	0.588	0.360	2.1202E-02	4
**Racgap1**	0.9325	0.784	0.472	5.0166E-06	4
**Cdk2ap2**	0.9314	0.784	0.500	1.9943E-05	4
**S100a9**	0.9303	1.000	1.000	9.4143E-28	4
**Cib1**	0.9302	0.588	0.320	7.2822E-03	4
**Tor2a**	0.9302	0.588	0.325	1.0002E-02	4
**Syf2**	0.9300	0.701	0.474	1.3099E-03	4
**Ing1**	0.9294	0.588	0.347	2.4535E-02	4
**Rpa2**	0.9290	0.526	0.267	9.8589E-03	4
**Cldn13**	0.9260	0.670	0.419	2.4594E-03	4

Table 1

**Bpgm**	0.9258	0.969	0.851	7.7572E-11	4
**Cdt1**	0.9257	0.907	0.680	8.3087E-09	4
**Hmmr**	0.9246	0.660	0.373	4.4906E-04	4
**Mki67**	0.9244	1.000	0.936	3.6192E-20	4
**Gpsm3**	0.9241	0.660	0.396	3.0317E-03	4
**Arhgef18**	0.9231	0.660	0.355	4.9991E-04	4
**Slc40a1**	0.9226	0.649	0.381	3.5328E-03	4
**Hist1h3d**	0.9223	1.000	0.987	8.7595E-27	4
**Ankrd13a**	0.9206	0.948	0.693	1.5589E-10	4
**Prkab1**	0.9205	0.814	0.469	6.0327E-06	4
**Dcun1d1**	0.9196	0.608	0.310	6.7441E-04	4
**Adam10**	0.9174	0.608	0.394	3.7586E-02	4
**Asf1b**	0.9174	0.856	0.583	3.9440E-07	4
**Gtf2f1**	0.9174	0.773	0.444	3.5262E-06	4
**Klf16**	0.9174	0.629	0.368	2.4959E-03	4
**Ddx27**	0.9174	0.588	0.360	2.5724E-02	4
**Hist1h2bk**	0.9173	1.000	0.891	1.2693E-17	4
**Map2k3**	0.9163	0.691	0.434	2.7214E-03	4
**Trappc1**	0.9163	0.505	0.257	3.2603E-02	4
**Pnpo**	0.9159	0.753	0.475	1.1224E-04	4
**Ppp1r15b**	0.9159	0.649	0.353	7.1007E-04	4
**Tnrc6b**	0.9159	0.608	0.375	4.7043E-02	4
**Knstrn**	0.9158	0.814	0.543	9.4388E-06	4
**Tnfrsf14**	0.9151	0.773	0.460	3.5422E-05	4
**Cpox**	0.9145	0.938	0.782	3.5758E-10	4
**Nde1**	0.9140	0.918	0.591	1.2424E-08	4
**Tk1**	0.9127	0.990	0.809	6.2491E-13	4
**Tapt1**	0.9122	0.804	0.426	1.6477E-06	4
**Cd72**	0.9115	0.598	0.335	1.9025E-03	4
**Cmc2**	0.9105	0.557	0.299	1.0575E-02	4
**Top1**	0.9104	0.629	0.408	3.4108E-02	4
**Tent5c**	0.9080	0.990	0.855	2.1570E-12	4
**Pnp**	0.9078	0.979	0.771	1.8260E-12	4
**Aspm**	0.9077	0.649	0.375	3.8750E-03	4
**Cd79a**	0.9063	0.990	0.769	2.1408E-12	4
**Cerk**	0.9063	0.577	0.314	1.5834E-02	4
**Epc1**	0.9045	0.598	0.315	2.6500E-03	4
**Rhd**	0.9032	0.979	0.861	1.0807E-13	4
**Ank1**	0.9031	0.990	0.871	5.0118E-15	4
**Arpc4**	0.9019	0.629	0.378	1.2543E-02	4
**Htra2**	0.9016	0.773	0.508	2.4480E-04	4
**U2af1**	0.9010	0.814	0.543	2.1316E-05	4
**Zfand2b**	0.9008	0.608	0.333	3.8805E-03	4
**Prkcd**	0.9005	0.588	0.305	2.5674E-03	4

Table 1

**Rrm1**	0.9005	0.753	0.441	3.2068E-05	4
**Exosc8**	0.8999	0.691	0.419	6.9129E-04	4
**Ppbp**	0.8989	0.742	0.518	1.3073E-02	4
**5031439G07Rik**	0.8986	0.639	0.376	5.4050E-03	4
**Mcm3**	0.8986	0.969	0.779	3.1012E-09	4
**Myo9b**	0.8974	0.536	0.292	3.0203E-02	4
**Slc25a51**	0.8967	0.629	0.348	8.4962E-03	4
**Grk2**	0.8963	0.938	0.716	5.2516E-09	4
**Smap2**	0.8960	0.825	0.558	1.0719E-06	4
**Farsb**	0.8960	0.742	0.455	1.4953E-04	4
**Zbtb7a**	0.8956	0.856	0.563	8.6287E-06	4
**Cln8**	0.8952	0.588	0.325	7.8695E-03	4
**Smc4**	0.8952	0.814	0.495	5.5654E-06	4
**Nelfcd**	0.8946	0.670	0.419	8.7423E-04	4
**Tcf20**	0.8943	0.711	0.404	1.2036E-04	4
**Ticrr**	0.8939	0.742	0.436	1.2312E-04	4
**E130309D02Rik**	0.8938	0.557	0.297	1.4878E-02	4
**Rcsd1**	0.8908	0.722	0.424	5.0727E-05	4
**Lmnb1**	0.8906	0.990	0.855	1.1704E-13	4
**Spc25**	0.8905	0.660	0.386	8.8299E-04	4
**Zcchc8**	0.8904	0.485	0.241	4.0217E-02	4
**Dcaf12**	0.8904	0.711	0.477	8.9010E-03	4
**U2surp**	0.8899	0.660	0.413	3.2354E-03	4
**Hist1h2bj**	0.8899	1.000	0.998	8.9488E-32	4
**March8**	0.8880	0.804	0.558	1.0237E-05	4
**Eif3a**	0.8863	0.608	0.332	6.7406E-03	4
**Cdca5**	0.8858	0.701	0.371	2.3628E-05	4
**Ermap**	0.8852	0.990	0.851	3.6312E-12	4
**Ccnb2**	0.8843	0.691	0.441	2.3051E-02	4
**Mthfd1l**	0.8835	0.608	0.365	2.7077E-02	4
**Slc9a3r1**	0.8804	0.804	0.545	5.1211E-04	4
**Spc24**	0.8797	0.907	0.630	1.3997E-07	4
**Apobec3**	0.8791	0.918	0.695	4.1173E-09	4
**Sharpin**	0.8787	0.619	0.361	1.1144E-02	4
**Tcof1**	0.8786	0.691	0.391	7.2397E-04	4
**Pold1**	0.8786	0.629	0.380	7.1980E-03	4
**Gypa**	0.8777	0.938	0.691	9.9653E-09	4
**Myl4**	0.8769	0.866	0.622	9.4924E-05	4
**Bach2**	0.8757	0.629	0.318	4.7092E-04	4
**Kdm4a**	0.8753	0.680	0.411	5.4636E-03	4
**Pif1**	0.8746	0.588	0.330	1.9594E-02	4
**Ptdss2**	0.8744	0.814	0.561	3.4804E-06	4
**Hist1h1d**	0.8743	1.000	1.000	3.2471E-34	4
**Add2**	0.8736	0.959	0.784	2.1101E-09	4

Table 1

**Cdc42se2**	0.8724	0.588	0.320	7.1229E-03	4
**Suz12**	0.8703	0.649	0.373	1.8814E-03	4
**Nup210**	0.8703	0.773	0.553	4.3056E-04	4
**Ifitm6**	0.8691	0.619	0.347	1.0954E-02	4
**Pak1ip1**	0.8688	0.588	0.318	1.0963E-02	4
**Hist2h2ac**	0.8673	1.000	0.982	2.4474E-23	4
**Ppie**	0.8665	0.598	0.307	1.7874E-03	4
**Hist1h1b**	0.8660	0.990	0.886	1.5958E-17	4
**Ppp1cc**	0.8644	0.619	0.376	2.5924E-02	4
**Ucp2**	0.8636	1.000	0.914	1.1978E-16	4
**Pttg1**	0.8634	0.773	0.507	7.3896E-04	4
**Incenp**	0.8634	0.948	0.724	2.9266E-07	4
**Pou2af1**	0.8623	0.918	0.650	2.1781E-07	4
**Slc4a1**	0.8602	1.000	0.942	5.9208E-17	4
**Mcm2**	0.8595	0.979	0.868	7.7094E-14	4
**Syk**	0.8593	0.959	0.752	5.0360E-10	4
**Ncbp3**	0.8590	0.588	0.330	2.9440E-02	4
**Tcp11l2**	0.8588	0.907	0.647	9.3732E-06	4
**Gda**	0.8587	0.773	0.477	2.2830E-04	4
**Plac8**	0.8584	0.948	0.810	3.5351E-10	4
**Cnot10**	0.8584	0.567	0.309	1.6033E-02	4
**Capza2**	0.8579	0.629	0.351	4.2839E-03	4
**Csk**	0.8572	0.876	0.587	6.4836E-06	4
**Lamtor3**	0.8557	0.608	0.356	4.7186E-02	4
**Lyn**	0.8545	0.825	0.568	2.8421E-05	4
**Vrk1**	0.8543	0.660	0.361	1.6490E-03	4
**Gpx1**	0.8543	1.000	0.997	1.2592E-27	4
**Wfdc21**	0.8522	0.825	0.599	1.9079E-04	4
**Med17**	0.8522	0.691	0.403	1.0102E-03	4
**Ube2a**	0.8515	0.680	0.422	2.8841E-03	4
**Ptbp3**	0.8509	0.938	0.721	2.6435E-07	4
**Rpp21**	0.8508	0.722	0.474	3.0784E-03	4
**Was**	0.8505	0.722	0.517	2.6315E-03	4
**Abcg2**	0.8504	0.608	0.350	3.2077E-02	4
**Cdc42ep3**	0.8503	0.649	0.413	2.9993E-02	4
**Hist1h1a**	0.8498	1.000	1.000	8.1821E-32	4
**Cmas**	0.8497	0.649	0.360	5.7428E-03	4
**Snx5**	0.8497	0.567	0.307	2.1868E-02	4
**Pkig**	0.8490	0.928	0.691	1.4098E-07	4
**Odf2**	0.8490	0.691	0.447	3.7824E-03	4
**Atrx**	0.8478	0.629	0.358	2.3286E-02	4
**Ccdc71l**	0.8476	0.969	0.754	2.4047E-08	4
**Cenpf**	0.8473	0.649	0.363	1.7956E-03	4
**Pla2g12a**	0.8465	0.608	0.335	1.7744E-02	4

Table 1

**Car1**	0.8458	0.990	0.814	1.8540E-12	4
**Pirb**	0.8455	0.588	0.340	2.0583E-02	4
**Pqbp1**	0.8451	0.629	0.386	3.9011E-02	4
**Pxk**	0.8449	0.567	0.318	3.9651E-02	4
**Cabin1**	0.8438	0.825	0.568	8.8952E-05	4
**Alad**	0.8434	0.938	0.739	2.5761E-09	4
**Mmp8**	0.8434	0.588	0.337	2.5868E-02	4
**Hs6st1**	0.8414	0.784	0.502	8.5690E-05	4
**Ppp1r10**	0.8412	0.680	0.452	2.3265E-02	4
**Slc6a9**	0.8400	0.670	0.411	1.2741E-02	4
**Polr2j**	0.8396	0.773	0.574	4.6149E-03	4
**Ranbp10**	0.8387	0.959	0.757	2.1018E-08	4
**Nsl1**	0.8384	0.598	0.333	1.1007E-02	4
**Uckl1**	0.8383	0.639	0.365	3.6868E-03	4
**Spi1**	0.8378	0.701	0.389	3.1644E-04	4
**Tmod1**	0.8359	0.680	0.363	3.7433E-04	4
**Cstf2**	0.8359	0.691	0.417	2.6045E-03	4
**Hcls1**	0.8359	0.598	0.325	8.0121E-03	4
**Actl6a**	0.8359	0.649	0.416	4.2561E-02	4
**Rhag**	0.8352	0.907	0.711	6.3565E-08	4
**Hebp1**	0.8346	0.866	0.541	5.3396E-06	4
**March2**	0.8337	0.990	0.863	2.4165E-10	4
**Fam129c**	0.8334	0.598	0.322	1.2483E-02	4
**Cenpa**	0.8324	0.907	0.706	6.5549E-07	4
**Ncaph**	0.8323	0.918	0.690	1.2083E-06	4
**Coro1a**	0.8308	0.948	0.766	1.4360E-08	4
**Pa2g4**	0.8307	0.990	0.858	3.0646E-13	4
**Vars**	0.8306	0.732	0.442	5.1277E-03	4
**Rasgrp2**	0.8304	0.732	0.439	2.9912E-03	4
**Clec12a**	0.8301	0.701	0.391	7.3822E-04	4
**Ccar1**	0.8301	0.680	0.426	3.5464E-03	4
**Cdc25b**	0.8298	0.918	0.706	7.7249E-06	4
**Dmtn**	0.8298	0.938	0.777	5.3821E-08	4
**Atp13a2**	0.8292	0.763	0.543	5.8065E-03	4
**Fam111a**	0.8284	0.701	0.427	7.2632E-03	4
**Isg20**	0.8276	0.990	0.853	3.7202E-13	4
**Cd24a**	0.8269	1.000	0.974	2.4618E-20	4
**Fahd1**	0.8268	0.608	0.371	4.6475E-02	4
**Selplg**	0.8263	0.742	0.414	1.5448E-04	4
**Mocs1**	0.8258	0.876	0.599	1.1834E-05	4
**Ddx56**	0.8256	0.598	0.332	3.8986E-02	4
**Vti1a**	0.8253	0.763	0.500	2.3344E-03	4
**Pqlc1**	0.8249	0.691	0.422	6.2055E-03	4
**Hist1h1e**	0.8242	1.000	0.998	1.4370E-29	4

Table 1

**Mpp1**	0.8238	0.722	0.498	2.8862E-02	4
**Tmem9**	0.8232	0.753	0.442	3.2506E-04	4
**Kcnn4**	0.8216	0.918	0.647	1.8687E-07	4
**Ezh2**	0.8210	0.804	0.503	3.7542E-05	4
**Fbxo7**	0.8207	0.701	0.449	7.6626E-03	4
**Urod**	0.8198	0.979	0.845	4.5697E-10	4
**Psma2**	0.8198	0.649	0.353	2.8531E-03	4
**Cdk1**	0.8195	0.918	0.655	4.0521E-06	4
**Farsa**	0.8188	0.639	0.406	4.5908E-02	4
**Supt4a**	0.8182	0.619	0.345	1.6599E-02	4
**Hist1h2bb**	0.8168	1.000	1.000	3.5453E-32	4
**Fcrla**	0.8167	0.660	0.413	2.0928E-02	4
**Tifa**	0.8160	0.660	0.427	4.7750E-02	4
**Mtmr3**	0.8158	0.856	0.640	2.5883E-05	4
**Rhog**	0.8154	0.680	0.391	2.6253E-03	4
**Tmem14c**	0.8147	0.825	0.607	4.1648E-05	4
**Ccna2**	0.8141	0.969	0.779	4.2499E-08	4
**Uck2**	0.8141	0.918	0.634	2.7028E-06	4
**Arhgap45**	0.8138	0.825	0.538	7.6605E-05	4
**Cd37**	0.8114	0.938	0.748	9.8357E-06	4
**Net1**	0.8114	0.711	0.467	6.1109E-03	4
**Plcg2**	0.8091	0.742	0.444	7.4492E-04	4
**Hba-a2**	0.8090	1.000	1.000	3.8939E-30	4
**Txnrd2**	0.8083	0.680	0.437	1.7385E-02	4
**Dut**	0.8070	0.918	0.733	1.2556E-05	4
**Hist1h3b**	0.8059	1.000	0.998	8.3123E-29	4
**Nop56**	0.8058	0.722	0.472	2.0695E-02	4
**Ggnbp2**	0.8058	0.526	0.269	4.3724E-02	4
**Hvcn1**	0.8057	0.845	0.550	4.4654E-05	4
**Fen1**	0.8045	0.887	0.614	1.5749E-05	4
**Laptm5**	0.8044	0.938	0.728	7.2719E-08	4
**Mxd1**	0.8041	0.742	0.483	1.6371E-03	4
**Spsb3**	0.8039	0.649	0.406	2.6305E-02	4
**Ube2l6**	0.8027	1.000	0.937	3.2671E-15	4
**Atp2a3**	0.8017	0.742	0.488	1.5617E-02	4
**Fam117a**	0.8011	0.990	0.955	1.7576E-17	4
**Sec11c**	0.8009	0.856	0.639	7.9984E-05	4
**Hdac1**	0.7980	0.928	0.649	1.1627E-06	4
**Hbb-bs**	0.7975	1.000	1.000	1.6341E-27	4
**Cecr2**	0.7975	0.845	0.592	2.8260E-04	4
**Rab27a**	0.7970	0.649	0.393	4.4532E-02	4
**Ncoa4**	0.7960	0.660	0.427	3.8145E-02	4
**Seh1l**	0.7953	0.619	0.343	1.0364E-02	4
**Pim1**	0.7948	0.979	0.799	4.7026E-09	4

Table 1

**Txnl1**	0.7944	0.742	0.411	1.1874E-04	4
**Hist1h3c**	0.7943	1.000	0.998	2.2237E-25	4
**Arhgdib**	0.7936	1.000	0.969	1.2239E-16	4
**Nme1**	0.7932	0.825	0.561	1.3727E-04	4
**Otud5**	0.7918	0.959	0.779	6.7372E-09	4
**Rtf1**	0.7910	0.722	0.469	1.4647E-02	4
**Uhrf1**	0.7907	0.856	0.668	5.0539E-04	4
**Rsad2**	0.7907	0.959	0.785	4.1063E-05	4
**Atic**	0.7904	0.722	0.477	1.9108E-02	4
**Bcl2l1**	0.7899	0.711	0.450	2.0672E-02	4
**Gata1**	0.7885	0.948	0.729	2.8030E-07	4
**Nsd2**	0.7884	0.763	0.498	4.0747E-03	4
**Elof1**	0.7884	0.649	0.347	1.8693E-03	4
**Ppp4c**	0.7883	0.876	0.634	7.4101E-05	4
**Smarcc1**	0.7874	0.856	0.578	1.0601E-04	4
**Hemgn**	0.7862	0.990	0.896	7.3980E-11	4
**Ddb1**	0.7856	0.835	0.627	1.6271E-04	4
**Lyl1**	0.7849	0.742	0.482	7.3373E-03	4
**Ube2s**	0.7841	0.948	0.814	2.8346E-08	4
**Tut7**	0.7840	0.794	0.554	2.9620E-04	4
**Snca**	0.7832	0.979	0.927	1.1460E-10	4
**Casp2**	0.7831	0.784	0.589	5.2205E-03	4
**Fcer1g**	0.7825	0.763	0.535	5.0942E-03	4
**Anp32e**	0.7822	0.990	0.964	1.6130E-17	4
**Gch1**	0.7820	0.845	0.563	1.2034E-03	4
**Tnfaip2**	0.7811	0.990	0.924	1.0211E-10	4
**Map3k1**	0.7805	0.918	0.731	3.6963E-06	4
**H2afx**	0.7799	0.990	0.964	9.3674E-17	4
**Tyms**	0.7794	0.732	0.485	2.0749E-02	4
**Lgals9**	0.7787	0.804	0.583	1.4884E-03	4
**Limd2**	0.7780	0.897	0.718	1.0807E-05	4
**Hsp90aa1**	0.7778	0.804	0.554	4.2780E-03	4
**Rab8b**	0.7777	0.691	0.459	2.9261E-02	4
**Hist1h1c**	0.7777	1.000	1.000	1.2044E-34	4
**Rnf213**	0.7763	0.670	0.427	2.3286E-02	4
**Hipk1**	0.7751	0.753	0.541	1.7754E-02	4
**Slirp**	0.7745	0.711	0.479	3.0221E-02	4
**Mlec**	0.7743	0.866	0.573	5.0255E-06	4
**Dgkd**	0.7740	0.753	0.507	1.3993E-03	4
**Brpf3**	0.7731	0.948	0.789	7.9718E-07	4
**Glrx5**	0.7723	1.000	0.969	1.0781E-16	4
**Ncl**	0.7723	0.959	0.883	1.2888E-08	4
**Msrb1**	0.7710	0.887	0.708	7.3744E-05	4
**Dpf2**	0.7709	0.825	0.634	1.1312E-03	4

Table 1

**Gypc**	0.7708	0.856	0.653	1.6536E-05	4
**Slc43a3**	0.7707	0.753	0.521	3.5351E-02	4
**Tmpo**	0.7706	1.000	0.972	2.0449E-21	4
**Ccnf**	0.7703	0.773	0.579	3.9428E-03	4
**Hnrnph2**	0.7700	0.722	0.457	1.2002E-02	4
**Ilk**	0.7698	0.794	0.518	8.1341E-03	4
**Cxxc1**	0.7697	0.753	0.521	2.3524E-03	4
**Mns1**	0.7697	0.814	0.523	1.6239E-03	4
**Hmbs**	0.7695	1.000	0.932	1.0368E-13	4
**Bola1**	0.7688	0.680	0.429	1.8068E-02	4
**Nudt1**	0.7688	0.588	0.310	2.0061E-02	4
**Srrt**	0.7683	0.979	0.759	3.6882E-08	4
**Gsr**	0.7680	0.845	0.604	5.4408E-04	4
**Slbp**	0.7679	1.000	0.889	3.1451E-10	4
**Kifc1**	0.7676	0.897	0.685	2.6428E-05	4
**Pglyrp1**	0.7669	0.948	0.823	4.8062E-06	4
**Dynll1**	0.7666	0.732	0.480	1.1291E-02	4
**Hdac2**	0.7659	0.639	0.384	2.2561E-02	4
**Ppp1r15a**	0.7654	0.979	0.856	1.5779E-09	4
**Lig1**	0.7651	0.845	0.604	1.6740E-03	4
**Fip1l1**	0.7640	0.763	0.533	7.5062E-03	4
**Ncf1**	0.7620	0.948	0.815	5.8024E-08	4
**Cpeb4**	0.7602	0.887	0.685	5.8859E-05	4
**Rad23a**	0.7601	0.990	0.883	9.0930E-11	4
**Cyfip2**	0.7595	0.959	0.769	3.5421E-06	4
**Abcf1**	0.7550	0.907	0.667	3.3137E-05	4
**Sri**	0.7546	0.938	0.809	6.9847E-07	4
**Cybb**	0.7528	0.784	0.535	8.0111E-03	4
**Dnmt1**	0.7523	0.928	0.713	3.0536E-06	4
**Serinc3**	0.7518	0.979	0.785	1.1505E-08	4
**Psmd13**	0.7502	0.835	0.591	2.8566E-03	4
**Sirpa**	0.7494	0.825	0.508	1.6299E-04	4
**Hcfc1**	0.7488	0.711	0.470	3.4461E-02	4
**Tfrc**	0.7485	1.000	0.937	3.8263E-11	4
**Prc1**	0.7485	0.887	0.637	6.4915E-04	4
**Rnf167**	0.7484	0.649	0.386	2.4693E-02	4
**Mpst**	0.7467	0.722	0.464	1.7241E-02	4
**Pygl**	0.7428	0.804	0.556	2.6289E-03	4
**Cnp**	0.7426	0.959	0.837	7.1144E-06	4
**Ube2o**	0.7399	0.845	0.576	1.1981E-03	4
**Dtymk**	0.7388	0.825	0.586	4.9593E-03	4
**E2f4**	0.7364	0.990	0.937	2.6669E-12	4
**Pck2**	0.7337	0.804	0.647	1.0936E-02	4
**Srsf1**	0.7332	1.000	0.911	7.4873E-09	4

Table 1

**Dnm2**	0.7328	0.773	0.526	3.7760E-02	4
**Dnajc9**	0.7317	0.990	0.825	1.4485E-08	4
**Rbm38**	0.7307	0.763	0.510	3.3505E-02	4
**Tmcc2**	0.7307	1.000	0.995	7.6104E-18	4
**H2afv**	0.7301	0.979	0.858	9.9662E-08	4
**Spib**	0.7300	0.887	0.591	1.8382E-04	4
**Rcc2**	0.7293	0.969	0.845	7.7921E-09	4
**Snrpf**	0.7290	0.773	0.505	8.8488E-03	4
**Sfxn1**	0.7285	0.742	0.492	2.1895E-02	4
**Rb1**	0.7271	0.742	0.493	2.4164E-02	4
**Klhl7**	0.7257	0.753	0.517	2.8366E-02	4
**Psme3**	0.7239	0.804	0.520	9.1525E-03	4
**Samd14**	0.7212	0.938	0.759	1.2720E-04	4
**Eml3**	0.7193	0.691	0.434	4.9807E-02	4
**Dnajb2**	0.7193	0.845	0.554	1.5072E-03	4
**Ikzf1**	0.7188	0.856	0.718	3.0937E-03	4
**Epb41**	0.7163	0.990	0.876	3.4654E-08	4
**Impdh2**	0.7161	0.876	0.644	1.8498E-04	4
**Abcb10**	0.7141	0.990	0.878	8.8933E-10	4
**Vasp**	0.7134	1.000	0.911	4.0552E-10	4
**Rnf126**	0.7129	0.763	0.523	1.0819E-02	4
**Cdr2**	0.7121	0.948	0.823	1.8655E-06	4
**Parp1**	0.7113	0.866	0.616	3.0775E-04	4
**Eaf1**	0.7089	0.753	0.477	1.0989E-02	4
**Tubb4b**	0.7051	0.959	0.926	4.6989E-09	4
**Kpnb1**	0.7047	0.773	0.563	9.7372E-03	4
**Ptp4a3**	0.7039	0.979	0.855	5.2331E-07	4
**Atp6v0d1**	0.7032	0.856	0.592	9.5589E-04	4
**Plekho2**	0.7028	0.835	0.606	1.1838E-03	4
**Usp25**	0.7025	0.876	0.616	2.8866E-04	4
**Ckap5**	0.7022	0.691	0.419	3.7131E-02	4
**H2afy**	0.7000	0.969	0.833	8.7388E-07	4
**Vbp1**	0.6998	0.804	0.513	2.4446E-03	4
**Pf4**	0.6980	0.979	0.901	5.6569E-03	4
**Anxa1**	0.6977	0.969	0.848	9.0861E-06	4
**Ighm**	0.6975	1.000	0.965	6.1307E-14	4
**Vps26a**	0.6962	0.742	0.462	1.3474E-02	4
**Fech**	0.6956	1.000	0.959	2.7651E-12	4
**Cdca3**	0.6937	0.969	0.815	1.2850E-06	4
**Ubald2**	0.6932	0.990	0.868	1.5965E-08	4
**Hnrnpa2b1**	0.6929	1.000	0.998	5.1814E-19	4
**Cct8**	0.6928	0.773	0.503	1.4140E-02	4
**Npm1**	0.6911	0.928	0.721	3.5150E-04	4
**Necap2**	0.6891	0.897	0.733	1.1475E-04	4

Table 1

**Ndufb1-ps**	0.6874	0.825	0.538	6.1024E-04	4
**Eif6**	0.6873	0.918	0.672	5.9418E-04	4
**Usp15**	0.6867	0.835	0.612	2.3059E-02	4
**Cbx1**	0.6857	0.835	0.691	4.1754E-03	4
**Sipa1**	0.6849	0.825	0.581	3.1454E-02	4
**Smarca4**	0.6837	0.990	0.972	1.6064E-12	4
**Scand1**	0.6835	0.948	0.911	7.6226E-10	4
**Pfn1**	0.6818	1.000	0.998	4.1968E-20	4
**Hist1h4h**	0.6799	0.990	0.856	2.1165E-08	4
**Wnk1**	0.6795	0.825	0.535	1.3262E-03	4
**Cyba**	0.6764	0.979	0.939	1.0606E-10	4
**Cct2**	0.6745	0.876	0.635	3.4020E-03	4
**Btg2**	0.6741	0.835	0.548	1.8844E-03	4
**Copz1**	0.6729	0.732	0.497	4.3446E-02	4
**Tmx4**	0.6706	0.680	0.360	7.2310E-03	4
**Pigq**	0.6694	0.990	0.891	9.0121E-08	4
**H2-D1**	0.6683	1.000	0.946	2.3663E-12	4
**Trak2**	0.6668	0.959	0.771	3.4420E-05	4
**Rnf123**	0.6667	0.784	0.579	1.1686E-02	4
**Zmat2**	0.6653	0.948	0.802	3.4411E-05	4
**Dap3**	0.6641	0.814	0.602	1.3791E-02	4
**Igkc**	0.6627	0.990	0.815	1.6708E-05	4
**Pabpc1**	0.6627	1.000	0.997	1.5555E-16	4
**Sf3a1**	0.6624	0.876	0.632	2.3835E-03	4
**C1qc**	0.6624	0.794	0.528	7.9617E-03	4
**Trim28**	0.6597	0.784	0.538	1.5079E-02	4
**Dazap2**	0.6593	0.990	0.866	1.0721E-05	4
**H3f3b**	0.6584	0.928	0.810	1.8233E-05	4
**Sh3bgrl3**	0.6562	0.959	0.766	1.0676E-05	4
**Oaz1**	0.6555	1.000	1.000	1.3525E-19	4
**Arpp19**	0.6552	0.887	0.657	3.0105E-03	4
**Bud31**	0.6543	0.897	0.680	9.6565E-03	4
**Actb**	0.6541	1.000	1.000	6.4476E-24	4
**Sf3b3**	0.6536	0.722	0.469	4.2745E-02	4
**1810058I24Rik**	0.6530	0.907	0.749	1.2782E-02	4
**Nsmce4a**	0.6526	0.928	0.853	1.2776E-05	4
**Hnrnpul1**	0.6524	0.856	0.673	1.2363E-03	4
**Klhdc3**	0.6498	0.722	0.467	4.7340E-02	4
**Hectd4**	0.6476	0.845	0.624	4.0577E-02	4
**Pspc1**	0.6470	0.876	0.678	2.5989E-02	4
**Cebpd**	0.6468	0.907	0.693	6.7087E-03	4
**Ybx3**	0.6445	1.000	0.988	9.8817E-16	4
**Smdt1**	0.6433	0.897	0.769	1.7372E-04	4
**Sumo2**	0.6433	0.856	0.642	1.6087E-02	4

Table 1

**Tgfb1**	0.6417	0.990	0.980	5.3719E-13	4
**Eif4a1**	0.6410	0.969	0.822	3.2121E-05	4
**Clic1**	0.6408	0.979	0.828	1.0067E-05	4
**Phf12**	0.6401	0.897	0.766	2.8384E-03	4
**Cd47**	0.6393	0.979	0.929	7.4517E-07	4
**Srsf5**	0.6392	0.856	0.627	2.2216E-02	4
**Anapc5**	0.6392	0.979	0.929	2.9000E-08	4
**Ssr4**	0.6369	0.763	0.485	2.4194E-02	4
**Hnrnpd**	0.6355	0.866	0.653	1.0126E-02	4
**Prdx2**	0.6350	0.990	0.917	1.5323E-07	4
**Lsm4**	0.6346	0.990	0.960	4.3484E-10	4
**Ebna1bp2**	0.6331	0.835	0.655	3.4057E-02	4
**Ptprc**	0.6320	0.794	0.596	4.5128E-02	4
**Stk24**	0.6284	0.763	0.500	4.1446E-02	4
**Ssna1**	0.6262	0.907	0.807	6.2003E-04	4
**Zeb2**	0.6256	0.948	0.769	2.7893E-04	4
**Poldip3**	0.6256	0.948	0.827	2.2666E-04	4
**H2afz**	0.6235	1.000	0.992	4.1697E-14	4
**C3**	0.6226	0.814	0.576	2.0553E-02	4
**Eef1g**	0.6210	0.990	0.921	4.2027E-07	4
**Clta**	0.6202	1.000	0.972	9.6318E-14	4
**Emb**	0.6182	0.701	0.416	1.3872E-02	4
**Arpc1b**	0.6180	0.938	0.837	3.4811E-03	4
**Rhoa**	0.6161	0.907	0.653	2.3009E-04	4
**Arrb1**	0.6148	0.887	0.680	1.0173E-02	4
**Arl6ip1**	0.6138	1.000	0.975	9.0649E-09	4
**Atpif1**	0.6124	0.990	0.873	1.1379E-05	4
**Pgp**	0.6113	0.876	0.736	2.1369E-02	4
**Eif3f**	0.6073	0.845	0.690	4.2795E-02	4
**Baz1b**	0.6040	0.938	0.830	3.9479E-04	4
**Tsc22d4**	0.6011	1.000	0.924	1.3033E-05	4
**Tbc1d10b**	0.6011	0.928	0.731	8.8307E-04	4
**Clk3**	0.5982	0.897	0.685	1.3260E-02	4
**Srsf6**	0.5917	1.000	0.985	1.6730E-10	4
**Aqp1**	0.5914	1.000	1.000	1.5971E-16	4
**Eif5**	0.5913	0.969	0.888	4.1150E-05	4
**Nusap1**	0.5911	1.000	0.914	1.3755E-04	4
**Eef1a1**	0.5898	1.000	0.997	2.5698E-18	4
**Hnrnpu**	0.5897	1.000	1.000	3.4311E-14	4
**Sf3b5**	0.5867	0.969	0.860	1.0904E-04	4
**Cotl1**	0.5863	0.928	0.799	4.7521E-04	4
**Erh**	0.5859	0.918	0.672	3.0035E-02	4
**Tmsb4x**	0.5829	1.000	1.000	5.8186E-21	4
**Naca**	0.5820	0.990	0.970	1.4488E-10	4

Table 1

**Hnrnpa0**	0.5800	1.000	1.000	6.0754E-15	4
**Supt16**	0.5798	0.876	0.670	2.5262E-02	4
**Sdcbp**	0.5781	0.969	0.861	8.5820E-05	4
**Vamp8**	0.5760	0.969	0.855	8.4876E-04	4
**Wdr26**	0.5744	0.990	0.926	3.9407E-07	4
**Mapk1**	0.5699	0.959	0.894	8.7702E-05	4
**Klf13**	0.5697	1.000	0.924	1.5343E-07	4
**Crebzf**	0.5656	0.938	0.840	1.6852E-03	4
**Zyx**	0.5649	0.979	0.936	1.4408E-02	4
**Tln1**	0.5600	0.948	0.817	2.1871E-02	4
**Rack1**	0.5592	1.000	1.000	8.0516E-20	4
**Serbp1**	0.5590	1.000	0.992	6.9654E-14	4
**Cfl1**	0.5589	1.000	0.990	8.2958E-12	4
**Ly6e**	0.5543	0.990	0.893	2.7117E-04	4
**Rnf10**	0.5542	1.000	0.944	4.6520E-08	4
**Gnai2**	0.5533	1.000	1.000	9.6134E-12	4
**Grina**	0.5441	0.969	0.810	3.4572E-03	4
**Spcs3**	0.5435	0.928	0.815	2.2096E-02	4
**Ppp2ca**	0.5311	0.979	0.838	1.5275E-03	4
**Anp32b**	0.5269	1.000	0.997	2.2270E-12	4
**Nap1l1**	0.5244	1.000	0.931	2.7257E-06	4
**Psmc2**	0.5235	0.969	0.776	1.2497E-02	4
**Col22a1**	0.5215	0.948	0.889	8.6340E-03	4
**Picalm**	0.5100	0.928	0.896	9.5760E-03	4
**Rnf187**	0.5048	1.000	0.985	1.6816E-06	4
**Tpt1**	0.5038	1.000	0.974	5.2275E-07	4
**Mobp**	1.8994	0.337	0.136	5.5825E-03	5
**Mbp**	0.9297	0.872	0.744	5.1011E-04	5
**Cd79b**	0.9113	0.814	0.585	1.7958E-04	5
**Acp5**	0.8321	1.000	0.998	6.5692E-10	5
**Acta1**	0.8259	0.860	0.699	3.6496E-04	5
**Ctsk**	0.7897	1.000	0.998	1.1946E-05	5
**Mmp9**	0.7638	1.000	0.990	1.8240E-05	5
**Ltf**	0.5732	0.977	0.922	1.7942E-02	5
**Ighm**	0.5475	1.000	0.966	2.1678E-03	5
**Caly**	7.1220	0.364	0.001	1.0609E-45	6
**Hand1**	6.7594	0.500	0.007	1.8742E-48	6
**Epha10**	6.6890	0.364	0.003	1.2601E-40	6
**Gprin1**	6.6150	0.545	0.007	1.0773E-54	6
**Areg**	6.5370	0.455	0.006	1.2515E-45	6
**Hmx1**	6.4946	0.773	0.029	8.0389E-51	6
**Zdhhc22**	6.3671	0.273	0.003	1.4359E-27	6
**Unc5a**	6.2740	0.409	0.004	7.7581E-43	6
**Kcnh7**	6.2740	0.318	0.004	3.4424E-30	6

Table 1

**Rxfp1**	6.2740	0.182	0.001	7.4679E-19	6
**Hpca**	6.2740	0.182	0.001	7.4679E-19	6
**Th**	6.2196	1.000	0.098	4.2353E-39	6
**Syn2**	6.2036	1.000	0.075	3.7523E-46	6
**Insm2**	6.1745	0.273	0.003	1.5363E-27	6
**Dbh**	6.1327	0.864	0.057	4.7017E-41	6
**Vstm2l**	6.1220	0.318	0.004	3.7853E-30	6
**Ntrk1**	6.0655	0.955	0.068	2.9077E-45	6
**Hand2**	6.0535	1.000	0.112	1.2847E-35	6
**Phox2a**	5.9939	0.864	0.043	1.3507E-49	6
**Ccdc184**	5.9521	0.364	0.006	5.5273E-33	6
**B4galnt4**	5.9521	0.273	0.004	5.5646E-24	6
**Akain1**	5.9521	0.227	0.003	3.4908E-21	6
**Hcrtr1**	5.9521	0.227	0.004	5.2995E-18	6
**Hcn3**	5.9521	0.136	0.001	2.3425E-12	6
**Chga**	5.9179	0.955	0.081	3.1955E-39	6
**Pcsk1n**	5.8799	0.955	0.132	1.7291E-28	6
**Sncb**	5.8781	0.591	0.026	9.7461E-34	6
**Vip**	5.8726	0.864	0.066	6.7248E-37	6
**Syngr3**	5.8366	0.818	0.035	9.9444E-50	6
**Nrsn2**	5.8366	0.591	0.018	9.4284E-43	6
**Nacad**	5.8146	0.500	0.015	9.0753E-36	6
**Doc2b**	5.8077	0.682	0.026	3.2823E-43	6
**Chrna3**	5.7752	0.818	0.050	6.1685E-40	6
**Chd5**	5.7594	0.364	0.010	1.4869E-25	6
**Rims4**	5.7594	0.273	0.004	6.0900E-24	6
**Lrrtm1**	5.7594	0.273	0.004	6.0900E-24	6
**Grp**	5.7594	0.273	0.004	6.0900E-24	6
**9330159F19Rik**	5.7594	0.227	0.004	5.2995E-18	6
**Sst**	5.7150	0.864	0.043	1.8802E-49	6
**Rbfox1**	5.6890	0.182	0.003	5.2780E-15	6
**Prph**	5.6529	1.000	0.223	1.7259E-21	6
**Chrna7**	5.6440	0.500	0.018	4.1987E-32	6
**Iqsec3**	5.6302	0.182	0.006	2.9308E-10	6
**Stmn2**	5.6221	0.955	0.191	2.8093E-21	6
**Rab3b**	5.6160	0.818	0.088	2.1055E-26	6
**Elavl2**	5.6092	0.636	0.037	8.5620E-31	6
**Dusp26**	5.6042	0.227	0.009	5.7063E-12	6
**Nsg2**	5.5942	0.864	0.085	7.3926E-31	6
**Gap43**	5.5434	0.818	0.079	5.0453E-29	6
**Tbx20**	5.5370	0.364	0.010	1.7609E-25	6
**Brinp2**	5.5370	0.227	0.004	5.7676E-18	6
**Slc6a2**	5.5310	1.000	0.159	5.9772E-27	6
**Cyb561**	5.5132	0.864	0.085	5.5491E-31	6

Table 1

**Rtn1**	5.5106	0.864	0.072	7.6061E-34	6
**Ptprn2**	5.4826	0.773	0.032	5.3852E-48	6
**Scn3b**	5.4826	0.545	0.023	1.3319E-31	6
**Vgf**	5.4667	0.773	0.025	1.6789E-54	6
**Bex2**	5.4462	0.909	0.090	4.8835E-32	6
**Scg2**	5.4375	0.409	0.013	6.2194E-27	6
**Kcnn2**	5.4375	0.227	0.006	1.6710E-15	6
**Slc30a3**	5.4115	0.409	0.010	8.6300E-31	6
**6330403K07Rik**	5.3927	0.818	0.073	6.6489E-30	6
**Tmem179**	5.3827	0.636	0.032	9.7005E-34	6
**Zcchc12**	5.3809	0.636	0.032	2.2342E-33	6
**Npy**	5.3803	0.955	0.156	4.0326E-24	6
**Htr3a**	5.3671	0.682	0.023	6.7920E-46	6
**Disp2**	5.3671	0.591	0.031	2.6856E-30	6
**Syt17**	5.3671	0.591	0.032	3.3061E-29	6
**Dab1**	5.3671	0.136	0.003	4.2794E-09	6
**Prrt2**	5.3671	0.136	0.003	4.2794E-09	6
**Elfn2**	5.3671	0.136	0.003	4.2794E-09	6
**Rab33a**	5.3671	0.136	0.003	4.2794E-09	6
**Rab9b**	5.3671	0.136	0.003	4.2794E-09	6
**Slc18a3**	5.3391	0.500	0.018	5.4464E-32	6
**Syt4**	5.3340	0.727	0.043	6.5342E-36	6
**Tm4sf4**	5.3306	0.318	0.013	4.1458E-17	6
**Sncg**	5.3039	0.955	0.194	1.1439E-19	6
**Kcnq3**	5.3000	0.364	0.015	2.1096E-20	6
**Sult4a1**	5.2916	0.818	0.053	1.0253E-37	6
**Celf3**	5.2847	0.727	0.034	5.5887E-41	6
**Slc18a2**	5.2833	0.818	0.079	7.8198E-28	6
**Fbxo41**	5.2740	0.182	0.004	2.9954E-12	6
**Diras1**	5.2740	0.182	0.004	2.9954E-12	6
**Tro**	5.2740	0.182	0.004	2.9954E-12	6
**Ddc**	5.2681	0.727	0.060	3.5969E-27	6
**Unc13a**	5.2416	0.364	0.010	2.5344E-25	6
**Syp**	5.2339	0.864	0.069	8.8039E-35	6
**Stmn3**	5.2208	0.955	0.142	2.8972E-25	6
**Soga3**	5.2151	0.318	0.009	4.7013E-22	6
**Sez6**	5.2151	0.227	0.006	1.8469E-15	6
**Rundc3a**	5.1931	0.682	0.047	6.4695E-29	6
**Nrsn1**	5.1865	0.545	0.023	5.4146E-31	6
**Pdyn**	5.1745	0.273	0.007	1.0555E-18	6
**Osbpl10**	5.1745	0.273	0.007	1.0555E-18	6
**Tmem132c**	5.1745	0.227	0.007	1.6749E-13	6
**Tubb3**	5.1727	0.909	0.132	2.2811E-24	6
**Prkar1b**	5.1585	0.500	0.016	1.3859E-33	6

Table 1

**Gal**	5.1585	0.682	0.066	2.8505E-22	6
**Astn1**	5.1447	0.500	0.023	1.7229E-26	6
**Gpr85**	5.1447	0.318	0.009	5.6915E-22	6
**Nrg3**	5.1447	0.318	0.009	5.6915E-22	6
**Sgpp2**	5.1447	0.273	0.007	1.0839E-18	6
**Srrm3**	5.1447	0.273	0.009	8.5423E-17	6
**Pcsk1**	5.1220	0.364	0.010	2.9170E-25	6
**Ptpn5**	5.1220	0.318	0.010	4.2281E-20	6
**Ppp2r2c**	5.1156	0.500	0.031	1.4410E-21	6
**Gpr158**	5.1125	0.545	0.021	9.3753E-34	6
**D430019H16Rik**	5.1125	0.545	0.023	3.9680E-31	6
**Syn1**	5.1082	0.773	0.048	4.2700E-36	6
**Elavl3**	5.1041	0.455	0.025	2.6727E-21	6
**Kcnq2**	5.0989	0.636	0.040	4.5459E-29	6
**Ptprn**	5.0965	0.682	0.050	2.3121E-27	6
**Insrr**	5.0896	0.409	0.013	9.3325E-27	6
**Stac**	5.0776	0.409	0.015	3.7736E-25	6
**Rab3c**	5.0631	0.682	0.035	1.1705E-35	6
**Nap1l5**	5.0574	0.864	0.079	4.5787E-32	6
**Rab6b**	5.0280	0.591	0.046	1.9521E-22	6
**Avil**	5.0261	0.364	0.026	6.7623E-13	6
**Celf6**	5.0135	0.500	0.031	8.1732E-22	6
**Cadm3**	5.0098	0.909	0.065	1.5684E-40	6
**Pcdh10**	5.0087	0.636	0.032	4.7163E-33	6
**Snap25**	4.9902	0.955	0.123	2.4857E-28	6
**L1cam**	4.9900	0.955	0.094	3.5429E-34	6
**Ngfr**	4.9521	0.727	0.053	1.1034E-29	6
**Fxyd7**	4.9521	0.636	0.040	1.0611E-28	6
**Tmem151b**	4.9521	0.455	0.019	1.5300E-25	6
**Hs3st2**	4.9521	0.364	0.012	2.4209E-23	6
**Jph3**	4.9521	0.500	0.029	1.2347E-22	6
**Sh3gl2**	4.9521	0.409	0.018	2.4229E-22	6
**Ina**	4.9521	0.364	0.015	2.8029E-20	6
**Mab21l2**	4.9521	0.364	0.015	3.1452E-20	6
**Ttbk1**	4.9521	0.364	0.015	3.1452E-20	6
**Tmprss5**	4.9521	0.273	0.009	9.7730E-17	6
**Nyap2**	4.9521	0.273	0.012	8.9140E-14	6
**Dock3**	4.9521	0.273	0.012	8.9140E-14	6
**Dlgap3**	4.9521	0.227	0.007	1.8739E-13	6
**Dclk3**	4.9521	0.227	0.007	1.8739E-13	6
**Rprm**	4.9521	0.182	0.004	3.3237E-12	6
**Celf4**	4.9521	0.227	0.009	7.2913E-12	6
**Oprl1**	4.9521	0.182	0.006	3.5092E-10	6
**Panx2**	4.9521	0.182	0.006	3.5092E-10	6

Table 1

**Pnmal1**	4.9521	0.227	0.012	2.1280E-09	6
**Them7**	4.9521	0.136	0.004	6.4910E-07	6
**Igdcc3**	4.9521	0.136	0.004	6.4910E-07	6
**Armc2**	4.9521	0.136	0.004	6.4910E-07	6
**Snhg11**	4.9133	0.955	0.132	9.9960E-28	6
**Apba1**	4.9131	0.864	0.104	2.6453E-26	6
**Fabp7**	4.8966	0.727	0.070	1.9995E-23	6
**Cend1**	4.8955	0.727	0.062	1.3546E-26	6
**Dpysl5**	4.8696	0.682	0.047	1.6397E-28	6
**Hpcal4**	4.8548	0.773	0.057	5.9730E-32	6
**Cplx1**	4.8474	0.909	0.120	1.4132E-25	6
**Mapk8ip2**	4.8366	0.545	0.034	6.9213E-24	6
**Fam163a**	4.8366	0.409	0.016	1.5246E-23	6
**Syt5**	4.8366	0.318	0.018	1.2016E-13	6
**Cdk5r2**	4.8318	0.636	0.031	4.1252E-34	6
**Ret**	4.8188	0.636	0.048	2.2259E-24	6
**Scg5**	4.8146	0.682	0.059	7.6669E-24	6
**Mapk10**	4.8146	0.455	0.031	9.0773E-18	6
**Pirt**	4.8052	0.591	0.038	1.5139E-25	6
**Gng3**	4.8052	0.500	0.040	1.6033E-17	6
**Kcnt1**	4.8001	0.364	0.013	1.1963E-21	6
**Nudt17**	4.8001	0.318	0.013	5.9033E-17	6
**Ecel1**	4.7916	0.591	0.026	6.5600E-33	6
**Tmem59l**	4.7916	0.409	0.026	1.8895E-16	6
**Asic2**	4.7822	0.455	0.025	4.7174E-21	6
**Gm49027**	4.7770	0.500	0.048	7.6864E-15	6
**Chgb**	4.7715	0.500	0.023	3.5523E-26	6
**Bean1**	4.7715	0.455	0.023	3.4317E-22	6
**Nsg1**	4.7657	0.955	0.128	2.0599E-28	6
**Frmpd1**	4.7594	0.273	0.010	4.5197E-15	6
**Creg2**	4.7297	0.409	0.019	6.9610E-21	6
**Arhgap36**	4.7297	0.364	0.018	1.0353E-17	6
**Rnf112**	4.7297	0.364	0.019	9.0382E-17	6
**Lrrc24**	4.7297	0.227	0.009	8.2378E-12	6
**Lhfpl5**	4.7297	0.227	0.009	8.2378E-12	6
**Gm11549**	4.7297	0.182	0.009	2.5198E-07	6
**Sptbn4**	4.7297	0.182	0.009	2.5198E-07	6
**Uchl1**	4.7220	1.000	0.405	2.4377E-13	6
**Spock1**	4.7111	0.364	0.018	1.1293E-17	6
**Rnf208**	4.6890	0.364	0.016	6.9541E-19	6
**Igsf9b**	4.6890	0.364	0.016	6.9541E-19	6
**Mapt**	4.6890	0.636	0.078	4.3944E-16	6
**Ctxn2**	4.6890	0.182	0.007	1.4346E-08	6
**Cbarp**	4.6820	0.727	0.056	5.4292E-28	6

Table 1

**Scn9a**	4.6720	0.455	0.023	4.5292E-22	6
**Syn3**	4.6626	0.318	0.015	1.1668E-15	6
**Syt1**	4.6626	0.318	0.015	1.1668E-15	6
**Jph4**	4.6593	0.682	0.057	6.0264E-25	6
**Htr3b**	4.6525	0.455	0.021	3.6683E-24	6
**Syt2**	4.6525	0.455	0.022	6.1851E-23	6
**Elavl4**	4.6495	0.727	0.048	1.5226E-31	6
**Bmerb1**	4.6302	0.591	0.032	9.3568E-29	6
**Adra2b**	4.6302	0.364	0.021	8.8596E-16	6
**Bpifb5**	4.6302	0.136	0.006	2.3692E-05	6
**9430007A20Rik**	4.6302	0.136	0.006	2.3692E-05	6
**Cdh16**	4.6302	0.136	0.006	2.3692E-05	6
**Nxph3**	4.6302	0.136	0.006	2.3692E-05	6
**Mgat5b**	4.6302	0.136	0.006	2.3692E-05	6
**Vwa5b2**	4.6302	0.136	0.006	2.3692E-05	6
**Cadm2**	4.6302	0.136	0.006	2.3692E-05	6
**Aplp1**	4.6202	0.636	0.093	1.0058E-13	6
**Tmeff2**	4.6150	0.364	0.034	7.7186E-10	6
**Brsk2**	4.6110	0.545	0.026	2.0910E-28	6
**Amph**	4.6110	0.455	0.026	3.1135E-20	6
**Kif1a**	4.5984	0.500	0.032	1.3014E-20	6
**Chrnb4**	4.5984	0.455	0.032	3.4900E-17	6
**Dgkb**	4.5895	0.455	0.025	6.2586E-21	6
**Gnb3**	4.5895	0.409	0.023	5.5649E-18	6
**Drd2**	4.5895	0.273	0.012	1.2112E-13	6
**Fbll1**	4.5895	0.273	0.012	1.2112E-13	6
**Cyp2j12**	4.5895	0.227	0.012	2.4409E-09	6
**Tspyl4**	4.5766	0.636	0.068	2.3814E-18	6
**Mast1**	4.5736	0.364	0.018	1.1293E-17	6
**Maoa**	4.5598	0.500	0.028	2.7503E-23	6
**Apc2**	4.5515	0.500	0.046	2.8565E-15	6
**Sgip1**	4.5370	0.455	0.022	6.1851E-23	6
**Pcbd1**	4.5370	0.455	0.022	6.1851E-23	6
**Myt1l**	4.5370	0.227	0.010	1.9333E-10	6
**Spock2**	4.5337	0.818	0.163	7.7434E-16	6
**Snap91**	4.5247	0.773	0.048	1.8830E-35	6
**Ncald**	4.5115	0.545	0.051	9.0431E-17	6
**Epha5**	4.5115	0.409	0.026	3.2386E-16	6
**Reep1**	4.5046	0.909	0.163	1.2893E-20	6
**Rasl10b**	4.5046	0.364	0.021	1.1125E-15	6
**Tmc3**	4.4926	0.227	0.015	1.5077E-07	6
**Ctxn1**	4.4699	0.909	0.116	3.5626E-25	6
**Fbxl16**	4.4667	0.545	0.029	2.9298E-26	6
**Resp18**	4.4667	0.500	0.035	3.0746E-19	6

Table 1

**Oxtr**	4.4667	0.182	0.009	2.8045E-07	6
**Fam155a**	4.4667	0.182	0.009	2.8045E-07	6
**Tbc1d30**	4.4667	0.182	0.009	2.8045E-07	6
**Lgi1**	4.4667	0.182	0.009	2.8045E-07	6
**Zcchc18**	4.4496	0.364	0.023	4.8221E-14	6
**Ctnna2**	4.4375	0.500	0.028	5.8440E-23	6
**Garnl3**	4.4375	0.455	0.028	3.1564E-19	6
**Kif26b**	4.4375	0.455	0.028	4.2334E-19	6
**Ptger3**	4.4140	0.864	0.140	4.5562E-20	6
**Ppfia3**	4.4046	0.409	0.026	4.2338E-16	6
**Kcnc4**	4.4027	0.636	0.082	1.8659E-15	6
**Plekha6**	4.3995	0.409	0.029	8.3208E-15	6
**Vat1l**	4.3671	0.864	0.122	1.0771E-20	6
**Gnal**	4.3671	0.409	0.021	1.2551E-19	6
**Fsd1**	4.3671	0.318	0.021	8.5263E-12	6
**Cyp4x1**	4.3671	0.227	0.012	2.7987E-09	6
**Shisa9**	4.3671	0.227	0.012	2.7987E-09	6
**Scn2b**	4.3671	0.182	0.012	2.1747E-05	6
**Cacna2d2**	4.3671	0.182	0.012	2.1747E-05	6
**Srsf12**	4.3671	0.136	0.007	3.5449E-04	6
**P2rx2**	4.3671	0.136	0.007	3.5449E-04	6
**Mpp3**	4.3671	0.136	0.007	3.5449E-04	6
**Pcdha7**	4.3671	0.136	0.007	3.5449E-04	6
**Crmp1**	4.3391	0.500	0.037	1.9895E-18	6
**Kcnk10**	4.3306	0.455	0.028	5.6719E-19	6
**Atp2b2**	4.3306	0.409	0.028	2.4859E-15	6
**Eml5**	4.3306	0.409	0.028	2.4859E-15	6
**Sv2a**	4.3240	0.318	0.023	1.9796E-10	6
**Rasgrf1**	4.3240	0.318	0.023	1.9796E-10	6
**Pcsk2**	4.3146	0.364	0.019	1.7545E-16	6
**Sv2c**	4.3146	0.318	0.019	1.5132E-12	6
**Map7d2**	4.3000	0.409	0.031	3.4349E-14	6
**Trnp1**	4.2916	0.545	0.059	1.7000E-14	6
**Sgsm1**	4.2740	0.364	0.022	1.1347E-14	6
**Grhl3**	4.2740	0.182	0.010	3.2045E-06	6
**Dbndd1**	4.2740	0.182	0.010	3.2045E-06	6
**Kif5a**	4.2602	0.455	0.029	2.8222E-18	6
**9330182L06Rik**	4.2516	0.273	0.018	1.3732E-09	6
**Vgll3**	4.2516	0.273	0.018	1.3732E-09	6
**Ptprr**	4.2416	0.364	0.025	3.5692E-13	6
**Hap1**	4.2151	0.409	0.028	3.2608E-15	6
**Mab21l1**	4.2151	0.318	0.021	1.0486E-11	6
**Adra2c**	4.2151	0.227	0.013	2.7712E-08	6
**AW551984**	4.1931	0.364	0.031	9.5389E-11	6

Table 1

**Cacna1b**	4.1865	0.318	0.023	2.4565E-10	6
**Gria1**	4.1865	0.273	0.023	3.9802E-07	6
**Rgs7**	4.1745	0.636	0.048	7.6510E-24	6
**Atp6v0e2**	4.1745	0.273	0.016	2.3072E-10	6
**Nalcn**	4.1745	0.364	0.032	2.6999E-10	6
**Smim10l2a**	4.1745	0.227	0.016	9.9310E-07	6
**Map2**	4.1697	0.591	0.059	2.2522E-17	6
**Gprasp2**	4.1447	0.318	0.019	1.8510E-12	6
**Slc2a6**	4.1447	0.136	0.009	2.9239E-03	6
**Edn2**	4.1447	0.136	0.009	2.9239E-03	6
**Fam71e1**	4.1447	0.136	0.009	2.9239E-03	6
**Nt5c1a**	4.1220	0.364	0.022	1.4300E-14	6
**Cnksr2**	4.1220	0.318	0.022	6.0989E-11	6
**Slc29a4**	4.1220	0.273	0.021	3.4465E-08	6
**Faah**	4.1041	0.182	0.012	2.4468E-05	6
**Pcnx2**	4.1041	0.182	0.012	2.4468E-05	6
**Nrxn3**	4.1041	0.182	0.012	2.4468E-05	6
**Ndrg4**	4.1022	0.818	0.164	2.2577E-14	6
**Golga7b**	4.0896	0.364	0.025	4.4988E-13	6
**Pcbp3**	4.0853	0.682	0.087	5.8373E-17	6
**Nrn1**	4.0776	0.455	0.046	4.0946E-12	6
**Sprn**	4.0776	0.409	0.047	2.8093E-09	6
**Gdap1l1**	4.0776	0.227	0.013	3.0020E-08	6
**Atcay**	4.0776	0.227	0.015	2.0246E-07	6
**Epb41l4a**	4.0776	0.227	0.015	2.0246E-07	6
**Rab39b**	4.0776	0.182	0.013	1.2808E-04	6
**Ccdc106**	4.0776	0.182	0.015	5.2998E-04	6
**Sarm1**	4.0776	0.182	0.015	5.2998E-04	6
**Sctr**	4.0676	0.682	0.087	2.7506E-16	6
**Unc80**	4.0590	0.273	0.018	1.6384E-09	6
**Clvs1**	4.0590	0.273	0.018	1.6384E-09	6
**Rgs17**	4.0452	0.273	0.018	1.6427E-09	6
**Olfm2**	4.0395	0.591	0.041	3.6418E-23	6
**Gabrb3**	4.0395	0.545	0.044	7.5118E-19	6
**Syt11**	4.0378	0.545	0.070	3.9078E-12	6
**Ctnnd2**	4.0345	0.273	0.023	4.8229E-07	6
**Aldoc**	4.0287	0.591	0.076	5.3034E-13	6
**Dpp6**	4.0261	0.318	0.026	4.1439E-09	6
**Kcnn3**	4.0261	0.273	0.026	3.8278E-06	6
**Fbxo2**	4.0225	0.455	0.053	2.6844E-10	6
**Nmnat2**	4.0192	0.318	0.029	3.6696E-08	6
**Mllt11**	3.9972	0.818	0.151	2.1818E-15	6
**Thy1**	3.9943	0.955	0.131	1.6630E-24	6
**Scg3**	3.9886	0.455	0.053	1.6935E-10	6

Table 1

**Gata2**	3.9825	0.545	0.063	9.7985E-14	6
**Map1b**	3.9648	0.727	0.138	1.8136E-13	6
**Rgs9**	3.9521	0.636	0.054	1.6381E-21	6
**Dner**	3.9521	0.455	0.037	7.3484E-15	6
**Fbxo44**	3.9521	0.500	0.051	2.3289E-13	6
**Phyhipl**	3.9521	0.409	0.040	6.8089E-11	6
**Slc6a15**	3.9521	0.318	0.022	7.5267E-11	6
**Lrrc4b**	3.9521	0.364	0.031	1.2322E-10	6
**Srcin1**	3.9521	0.364	0.038	2.3981E-08	6
**Begain**	3.9521	0.273	0.022	1.5943E-07	6
**Arhgap22**	3.9521	0.273	0.022	1.5943E-07	6
**Ass1**	3.9521	0.227	0.016	1.1552E-06	6
**Gm42517**	3.9521	0.227	0.019	1.8036E-05	6
**Tmem169**	3.9521	0.182	0.013	1.3697E-04	6
**Col26a1**	3.9521	0.182	0.013	1.3697E-04	6
**Omg**	3.9521	0.182	0.013	1.3697E-04	6
**Kcnh8**	3.9521	0.182	0.013	1.3697E-04	6
**Fam131b**	3.9521	0.227	0.022	1.5636E-04	6
**Hcn1**	3.9521	0.182	0.016	1.9062E-03	6
**Tox2**	3.9521	0.182	0.019	1.5759E-02	6
**Col28a1**	3.9521	0.136	0.010	1.5884E-02	6
**Trim9**	3.9521	0.136	0.010	1.5884E-02	6
**Kif5c**	3.9235	0.591	0.069	1.5786E-14	6
**Gng4**	3.9223	0.636	0.069	9.5374E-18	6
**Eno2**	3.9217	0.727	0.125	9.9097E-14	6
**Rgs4**	3.9185	0.909	0.203	1.0550E-15	6
**Acsbg1**	3.9165	0.500	0.051	3.8262E-13	6
**Nyap1**	3.9126	0.455	0.050	4.7551E-11	6
**Eef1a2**	3.8992	0.818	0.167	9.0146E-15	6
**Nnat**	3.8887	0.773	0.188	1.4867E-10	6
**Nrcam**	3.8879	0.591	0.062	2.6963E-16	6
**Rims3**	3.8879	0.364	0.032	4.1678E-10	6
**Flrt1**	3.8850	0.591	0.059	4.0811E-17	6
**Ttll7**	3.8817	0.318	0.029	4.6088E-08	6
**Csdc2**	3.8741	0.545	0.051	4.8626E-16	6
**Sept3**	3.8741	0.318	0.026	5.1712E-09	6
**Wasf3**	3.8646	0.273	0.023	5.8389E-07	6
**Lix1**	3.8646	0.273	0.023	5.8389E-07	6
**Slc1a1**	3.8646	0.273	0.023	5.8389E-07	6
**Lrp11**	3.8569	0.500	0.063	1.2509E-10	6
**Celsr3**	3.8525	0.455	0.041	3.4089E-13	6
**Lrrc73**	3.8525	0.273	0.021	4.6004E-08	6
**Bex1**	3.8525	0.227	0.021	6.1227E-05	6
**Tppp3**	3.8376	1.000	0.424	3.6307E-13	6

Table 1

**Tmem151a**	3.8366	0.318	0.037	4.0398E-06	6
**Phf24**	3.8366	0.227	0.018	5.3765E-06	6
**Syt3**	3.8366	0.227	0.018	5.3765E-06	6
**Gm10643**	3.8366	0.227	0.018	5.3765E-06	6
**Pcdh9**	3.8366	0.227	0.018	5.3765E-06	6
**Svop**	3.8366	0.182	0.018	5.8510E-03	6
**Adra2a**	3.8301	0.545	0.050	1.6695E-16	6
**Stmn4**	3.8301	0.455	0.046	4.4627E-12	6
**Reep2**	3.8265	0.591	0.097	1.1845E-09	6
**Syt9**	3.8243	0.682	0.082	1.3223E-17	6
**Fam57b**	3.8146	0.409	0.031	1.0512E-13	6
**Stx1a**	3.8146	0.364	0.029	4.7633E-11	6
**Tmem35a**	3.8146	0.364	0.029	4.7633E-11	6
**Myt1**	3.8146	0.182	0.013	1.4466E-04	6
**Map1a**	3.8101	0.909	0.211	7.7031E-16	6
**Ckmt1**	3.8052	0.364	0.044	7.1622E-07	6
**Rimbp2**	3.8001	0.364	0.028	1.3512E-11	6
**Tox**	3.8001	0.318	0.028	1.8110E-08	6
**Plppr4**	3.7822	0.455	0.037	1.1486E-14	6
**Pianp**	3.7822	0.318	0.025	1.6542E-09	6
**Dpysl4**	3.7822	0.227	0.025	1.0451E-03	6
**Frmpd4**	3.7594	0.227	0.022	1.8409E-04	6
**Faxc**	3.7504	0.364	0.032	5.3926E-10	6
**Spred3**	3.7504	0.364	0.032	5.3926E-10	6
**Shank2**	3.7504	0.364	0.032	5.3926E-10	6
**Rph3a**	3.7456	0.455	0.043	9.3387E-13	6
**Arhgdig**	3.7297	0.409	0.040	1.0309E-10	6
**Kcnip4**	3.7297	0.227	0.019	2.1115E-05	6
**March11**	3.7297	0.227	0.019	2.1115E-05	6
**Lancl3**	3.7297	0.227	0.019	2.1115E-05	6
**Hmgcs2**	3.7297	0.273	0.029	3.1847E-05	6
**Sez6l2**	3.7150	0.409	0.044	1.1893E-09	6
**Pnmal2**	3.7150	0.364	0.046	8.9956E-07	6
**Tagln3**	3.7133	0.500	0.072	6.3655E-09	6
**Lonrf2**	3.7111	0.364	0.037	1.0375E-08	6
**Kif3c**	3.7111	0.455	0.072	6.2299E-07	6
**Ncam2**	3.7042	0.318	0.026	6.4470E-09	6
**Grin1**	3.7042	0.273	0.025	2.0487E-06	6
**Edn3**	3.7042	0.273	0.025	2.0487E-06	6
**Spock3**	3.6983	0.500	0.040	1.1385E-16	6
**Ttyh1**	3.6983	0.364	0.044	5.4417E-07	6
**Tmem178b**	3.6890	0.455	0.047	1.8517E-11	6
**Pcp4**	3.6890	0.182	0.016	2.1663E-03	6
**Nap1l3**	3.6890	0.182	0.016	2.1663E-03	6

Table 1

**Mcam**	3.6811	0.818	0.210	1.7375E-11	6
**Rab15**	3.6720	0.364	0.047	2.3200E-06	6
**Sox10**	3.6720	0.182	0.021	3.5962E-02	6
**Oprm1**	3.6626	0.318	0.031	1.6999E-07	6
**Adcy1**	3.6626	0.318	0.031	1.6999E-07	6
**Nrxn1**	3.6626	0.318	0.031	1.6999E-07	6
**A4galt**	3.6566	0.364	0.037	1.1317E-08	6
**Nefl**	3.6525	0.864	0.145	1.5544E-18	6
**Nrxn2**	3.6495	0.500	0.050	1.6501E-13	6
**Gata3**	3.6302	0.364	0.035	4.9382E-09	6
**Rgs11**	3.6302	0.273	0.028	1.5431E-05	6
**Gria2**	3.6302	0.227	0.019	2.2452E-05	6
**Cxxc4**	3.6302	0.364	0.054	2.6130E-05	6
**Tram1l1**	3.6302	0.227	0.021	7.1867E-05	6
**Wnt11**	3.6302	0.227	0.021	7.1867E-05	6
**Map3k13**	3.6302	0.227	0.021	7.1867E-05	6
**Tmem130**	3.6302	0.182	0.021	3.8063E-02	6
**Ephb6**	3.6302	0.182	0.021	3.8063E-02	6
**Esyt3**	3.6302	0.182	0.021	3.8063E-02	6
**Adcyap1r1**	3.6187	0.682	0.084	1.9969E-16	6
**Cadps**	3.6081	0.409	0.041	4.0076E-10	6
**Kcnh2**	3.6081	0.364	0.043	3.8504E-07	6
**Brsk1**	3.5959	0.636	0.081	1.5445E-14	6
**Zdhhc2**	3.5945	0.500	0.059	1.6290E-11	6
**Egfl8**	3.5895	0.227	0.025	1.2352E-03	6
**Parva**	3.5804	1.000	0.325	1.2633E-15	6
**Prune2**	3.5736	0.409	0.054	2.6822E-07	6
**Adra1b**	3.5736	0.182	0.018	6.6648E-03	6
**Cacna1a**	3.5674	0.545	0.063	5.4912E-13	6
**Map6**	3.5651	0.591	0.088	1.3087E-10	6
**Hid1**	3.5461	0.773	0.141	1.8902E-13	6
**Fam89a**	3.5461	0.500	0.066	8.9066E-10	6
**Foxd3**	3.5370	0.455	0.046	1.0313E-11	6
**Nudt11**	3.5370	0.318	0.032	5.0021E-07	6
**Napb**	3.5370	0.318	0.034	1.1853E-06	6
**Cntnap5a**	3.5370	0.227	0.022	2.1657E-04	6
**Sptbn2**	3.5370	0.273	0.046	2.0109E-02	6
**Stk32a**	3.5370	0.182	0.021	3.9568E-02	6
**Tuba1a**	3.5175	1.000	0.897	2.5089E-11	6
**Nphp4**	3.5115	0.273	0.026	6.8924E-06	6
**Lrrc3**	3.5115	0.273	0.026	6.8924E-06	6
**Gpr137c**	3.5115	0.273	0.026	6.8924E-06	6
**Ccdc92**	3.4926	0.591	0.070	5.4718E-14	6
**Fgf12**	3.4926	0.273	0.029	4.1338E-05	6

Table 1

**Isl1**	3.4926	0.273	0.031	9.1582E-05	6
**Bicdl1**	3.4826	0.455	0.047	2.6723E-11	6
**Dmkn**	3.4667	0.182	0.019	1.7986E-02	6
**Ric3**	3.4667	0.182	0.019	1.7986E-02	6
**Phactr1**	3.4667	0.182	0.019	1.7986E-02	6
**Fzd3**	3.4667	0.182	0.019	1.7986E-02	6
**Slitrk1**	3.4667	0.182	0.019	1.7986E-02	6
**Tcp11l1**	3.4573	0.682	0.082	1.8408E-16	6
**Magee1**	3.4573	0.318	0.040	2.7007E-05	6
**Slc6a17**	3.4573	0.273	0.043	1.0212E-02	6
**Syt7**	3.4496	0.455	0.069	3.7854E-07	6
**Ly6h**	3.4496	0.227	0.023	5.8795E-04	6
**Sbspon**	3.4375	0.227	0.028	6.0481E-03	6
**Adcy2**	3.4285	0.318	0.032	5.8409E-07	6
**Atp9a**	3.4216	0.909	0.266	1.0129E-12	6
**Rit2**	3.4216	0.364	0.035	7.0097E-09	6
**Asphd2**	3.4160	0.318	0.038	1.2641E-05	6
**Epb41l3**	3.4007	0.545	0.115	2.6955E-06	6
**Nptxr**	3.3671	0.455	0.057	6.3849E-09	6
**Rnf227**	3.3671	0.364	0.038	4.5476E-08	6
**Rph3al**	3.3671	0.318	0.032	6.2938E-07	6
**Mgat3**	3.3671	0.273	0.029	4.7451E-05	6
**Map9**	3.3671	0.318	0.043	1.1069E-04	6
**Adgrb1**	3.3671	0.318	0.046	3.0106E-04	6
**Shd**	3.3671	0.227	0.025	1.4585E-03	6
**Cspg5**	3.3671	0.182	0.019	1.8740E-02	6
**Susd2**	3.3512	0.591	0.116	8.1420E-08	6
**Gnao1**	3.3354	0.455	0.063	5.6947E-08	6
**Clip3**	3.3240	0.636	0.122	3.3276E-09	6
**Akap6**	3.3198	0.591	0.084	9.0903E-12	6
**Myh14**	3.3198	0.364	0.043	4.7655E-07	6
**Ttc9**	3.3146	0.318	0.040	2.9125E-05	6
**Minar2**	3.3146	0.273	0.040	4.2841E-03	6
**Faim2**	3.3000	0.273	0.029	5.0419E-05	6
**Ube2ql1**	3.3000	0.273	0.031	1.1190E-04	6
**Aatk**	3.2988	0.818	0.204	5.5159E-11	6
**Ccnjl**	3.2891	0.227	0.026	3.3447E-03	6
**Rnft2**	3.2822	0.318	0.050	1.3824E-03	6
**Tmod2**	3.2740	0.682	0.101	4.7068E-13	6
**Lsamp**	3.2740	0.318	0.044	2.1490E-04	6
**Ntng1**	3.2740	0.182	0.021	4.5226E-02	6
**Cntn4**	3.2740	0.182	0.021	4.5226E-02	6
**Omp**	3.2676	0.409	0.063	7.7185E-06	6
**Fmn1**	3.2602	0.409	0.056	7.8292E-07	6

Table 1

**Nol3**	3.2516	0.500	0.087	1.3531E-06	6
**Lgi4**	3.2516	0.318	0.035	3.8595E-06	6
**Samd10**	3.2516	0.273	0.035	8.9597E-04	6
**Azin2**	3.2443	0.545	0.065	3.1456E-12	6
**Caskin1**	3.2443	0.409	0.068	2.4143E-05	6
**Galnt17**	3.2416	0.364	0.048	8.2556E-06	6
**Ndn**	3.2359	0.909	0.229	1.5403E-11	6
**Fgf14**	3.2359	0.273	0.032	2.4829E-04	6
**Tubb2b**	3.2311	0.864	0.372	6.5502E-08	6
**Atl1**	3.2151	0.455	0.078	3.4277E-06	6
**Ppm1j**	3.2151	0.227	0.026	3.5065E-03	6
**Gabrg2**	3.2151	0.227	0.026	3.5065E-03	6
**Scml4**	3.2151	0.227	0.028	7.1611E-03	6
**Ggt7**	3.1972	0.318	0.035	4.1367E-06	6
**Amer2**	3.1972	0.318	0.037	8.7813E-06	6
**Apbb1**	3.1957	0.682	0.160	1.0568E-07	6
**Cplx2**	3.1951	0.727	0.210	9.5885E-08	6
**Fkbp1b**	3.1719	0.500	0.106	8.0574E-05	6
**Pex5l**	3.1678	0.318	0.040	3.3344E-05	6
**Fez1**	3.1636	0.455	0.053	1.2989E-09	6
**Snap47**	3.1577	0.727	0.145	3.9381E-10	6
**St6galnac5**	3.1447	0.318	0.040	3.6937E-05	6
**Parm1**	3.1192	0.364	0.076	1.3700E-02	6
**Tspan17**	3.1156	0.591	0.104	8.9722E-09	6
**Kif3a**	3.1156	0.455	0.068	3.3142E-07	6
**Dgkh**	3.1156	0.455	0.069	5.1244E-07	6
**Zfhx2**	3.1156	0.273	0.035	1.0430E-03	6
**Dhcr24**	3.1125	0.682	0.167	2.3463E-07	6
**Ppp2r2b**	3.1041	0.364	0.047	4.4386E-06	6
**Vat1**	3.0991	0.955	0.454	1.9374E-10	6
**Kcnab1**	3.0941	0.318	0.041	7.3472E-05	6
**Mtmr7**	3.0941	0.318	0.041	7.3472E-05	6
**B630019K06Rik**	3.0896	0.455	0.054	3.0763E-09	6
**Lingo1**	3.0870	0.409	0.070	1.0687E-04	6
**Syngr1**	3.0864	0.818	0.203	3.5109E-10	6
**Dtx1**	3.0776	0.364	0.046	2.7256E-06	6
**Kcnmb4**	3.0776	0.318	0.047	6.3510E-04	6
**Bicd1**	3.0776	0.227	0.031	2.7567E-02	6
**Alcam**	3.0676	0.364	0.066	2.1516E-03	6
**Bend6**	3.0590	0.273	0.037	1.9948E-03	6
**Igsf21**	3.0590	0.273	0.037	1.9948E-03	6
**Coprs**	3.0590	0.273	0.037	1.9948E-03	6
**Nefm**	3.0428	0.727	0.176	1.3755E-08	6
**Prmt2**	3.0378	0.636	0.129	7.7393E-08	6

Table 1

**Camk2n2**	3.0378	0.455	0.066	3.8453E-07	6
**Zc4h2**	3.0345	0.318	0.048	1.0729E-03	6
**Sox11**	3.0345	0.273	0.046	3.3507E-02	6
**Fgf1**	3.0304	0.909	0.310	2.5927E-09	6
**Mvd**	3.0292	0.636	0.125	1.2297E-08	6
**Cep170b**	3.0183	0.818	0.181	1.3715E-11	6
**Tac1**	3.0045	0.273	0.038	3.6643E-03	6
**Bbs1**	2.9979	0.318	0.041	8.2999E-05	6
**Adam23**	2.9979	0.318	0.044	2.6011E-04	6
**Evpl**	2.9979	0.273	0.044	2.4189E-02	6
**Kctd16**	2.9927	0.273	0.041	1.1336E-02	6
**Ncs1**	2.9886	0.773	0.191	1.7101E-09	6
**Cadm4**	2.9852	0.409	0.051	2.0346E-07	6
**Flywch2**	2.9852	0.409	0.057	1.9826E-06	6
**Ank3**	2.9634	0.727	0.157	2.6269E-09	6
**Tubb2a**	2.9521	0.909	0.465	3.1017E-06	6
**Rragd**	2.9521	0.409	0.062	8.8626E-06	6
**Bsn**	2.9521	0.364	0.048	1.1042E-05	6
**Camta1**	2.9521	0.455	0.091	1.6723E-04	6
**Psd**	2.9521	0.545	0.148	5.6926E-04	6
**Tlcd2**	2.9521	0.273	0.040	6.4886E-03	6
**Acot7**	2.9492	0.955	0.470	3.0262E-08	6
**Nefh**	2.9401	0.545	0.141	6.7103E-04	6
**Mapk8ip1**	2.9287	0.773	0.226	4.2189E-07	6
**Ttbk2**	2.9235	0.591	0.129	9.9520E-07	6
**Kif21a**	2.9094	0.545	0.119	2.6378E-05	6
**Plekhb2**	2.9077	0.591	0.090	5.0785E-10	6
**Agap2**	2.9077	0.318	0.047	8.0919E-04	6
**Plekha4**	2.9015	0.455	0.103	1.3289E-03	6
**Asl**	2.8918	0.773	0.210	1.0566E-08	6
**Atp6v1g2**	2.8788	0.591	0.128	1.7696E-06	6
**Stxbp1**	2.8615	0.591	0.148	2.8778E-05	6
**Plxna4**	2.8569	0.364	0.066	2.0944E-03	6
**Frs3**	2.8525	0.273	0.040	7.1252E-03	6
**Plppr3**	2.8525	0.273	0.041	1.1627E-02	6
**Gprasp1**	2.8477	0.455	0.107	2.8271E-03	6
**Rhbdd2**	2.8477	0.318	0.057	1.3413E-02	6
**Camk2b**	2.8410	0.636	0.142	3.2581E-07	6
**Spry2**	2.8366	0.455	0.070	1.8547E-06	6
**Rab3a**	2.8287	0.455	0.079	2.2029E-05	6
**Slc31a1**	2.8261	0.864	0.319	7.4495E-08	6
**Tcaf1**	2.8214	0.682	0.160	4.5762E-07	6
**Ank2**	2.7988	0.773	0.285	1.3645E-05	6
**Dipk1b**	2.7968	0.409	0.062	1.2548E-05	6

Table 1

**Bcam**	2.7822	0.636	0.148	1.0681E-06	6
**Lrrn2**	2.7822	0.318	0.051	3.6273E-03	6
**Itgb4**	2.7822	0.318	0.051	3.6273E-03	6
**Hdac11**	2.7715	0.455	0.088	1.0131E-04	6
**Arhgap28**	2.7699	0.500	0.075	9.4138E-08	6
**Fcor**	2.7645	0.364	0.057	2.8366E-04	6
**Sfrp1**	2.7594	0.409	0.082	2.3898E-03	6
**Mbip**	2.7594	0.273	0.046	4.6893E-02	6
**Fbxl20**	2.7391	0.455	0.073	5.4596E-06	6
**Trib1**	2.7391	0.364	0.070	8.4687E-03	6
**Rufy3**	2.7366	0.636	0.250	2.2368E-03	6
**Fam219a**	2.7267	0.636	0.198	6.4925E-04	6
**Tesc**	2.7233	0.409	0.095	1.1877E-02	6
**Olfm1**	2.7150	0.727	0.261	1.5174E-04	6
**Spry4**	2.7150	0.273	0.044	3.2443E-02	6
**Ttl**	2.7092	0.455	0.097	7.8329E-04	6
**Tenm4**	2.6836	0.545	0.137	2.7212E-04	6
**Nfasc**	2.6836	0.500	0.129	2.7688E-03	6
**Pfn2**	2.6812	0.500	0.141	1.6360E-02	6
**Ache**	2.6768	0.727	0.250	1.7910E-05	6
**Cystm1**	2.6652	0.591	0.159	3.5235E-04	6
**Slc22a17**	2.6586	0.773	0.232	2.1613E-07	6
**Akap7**	2.6463	0.545	0.120	5.6676E-05	6
**Pacsin1**	2.6463	0.500	0.116	6.4962E-04	6
**Ahi1**	2.6302	0.364	0.069	6.0716E-03	6
**Rap1gap**	2.6302	0.364	0.078	2.7350E-02	6
**Basp1**	2.6053	0.955	0.571	3.3328E-06	6
**Ncam1**	2.6047	0.636	0.266	1.7587E-02	6
**Adap1**	2.5959	0.636	0.156	9.6586E-06	6
**Hspb8**	2.5945	0.864	0.204	3.8989E-10	6
**Zfr2**	2.5651	0.500	0.081	9.7129E-07	6
**Scamp5**	2.5580	0.682	0.247	2.5458E-04	6
**5730409E04Rik**	2.5427	0.455	0.112	8.0852E-03	6
**Fam174b**	2.5370	0.545	0.148	2.6824E-03	6
**Hmgcs1**	2.5331	0.682	0.270	4.7708E-03	6
**Scube1**	2.5242	0.591	0.138	3.9689E-05	6
**Fam131a**	2.5232	0.455	0.091	3.9592E-04	6
**Flywch1**	2.5221	0.727	0.236	1.4665E-04	6
**Ids**	2.5135	0.500	0.138	1.3199E-02	6
**Meis1**	2.5115	0.455	0.101	2.8376E-03	6
**Inpp5f**	2.5046	0.409	0.081	2.2110E-03	6
**Alg2**	2.4984	0.545	0.172	1.3326E-02	6
**Plk3**	2.4808	0.455	0.084	1.2060E-04	6
**Hr**	2.4667	0.500	0.106	1.7668E-04	6

Table 1

**Cartpt**	2.4624	0.773	0.336	2.0041E-03	6
**Ralgds**	2.4204	0.682	0.298	1.8492E-02	6
**Sqle**	2.4186	0.545	0.140	1.6938E-03	6
**Pfkp**	2.4141	0.727	0.191	2.8216E-06	6
**Klc1**	2.3975	0.955	0.460	9.4554E-07	6
**Zc2hc1a**	2.3873	0.409	0.091	1.5198E-02	6
**Atp1b1**	2.3595	1.000	0.881	6.2548E-08	6
**Apod**	2.3586	0.818	0.347	2.1580E-04	6
**Tln2**	2.3545	0.636	0.242	4.3958E-02	6
**Msmo1**	2.3494	0.591	0.204	3.0774E-02	6
**Pmp22**	2.3465	0.955	0.581	1.3284E-07	6
**Dpysl2**	2.3358	0.909	0.495	1.5105E-03	6
**Abca8a**	2.3216	0.636	0.191	2.2591E-03	6
**Plscr4**	2.3165	0.455	0.117	3.5857E-02	6
**Cacnb3**	2.3061	0.727	0.211	1.2443E-05	6
**Rgmb**	2.2916	0.773	0.229	2.5597E-06	6
**Atp6v1b2**	2.2913	0.727	0.261	4.4016E-04	6
**Tbc1d7**	2.2891	0.364	0.076	4.3965E-02	6
**Capn5**	2.2891	0.455	0.120	4.5021E-02	6
**Sfrp5**	2.2862	0.682	0.194	1.3372E-04	6
**Vamp2**	2.2860	0.818	0.451	6.8959E-04	6
**Wdr6**	2.2754	0.682	0.244	4.3134E-03	6
**Dip2a**	2.2740	0.500	0.131	7.7263E-03	6
**Plekha5**	2.2740	0.409	0.087	1.0359E-02	6
**Rcan3**	2.2665	0.636	0.229	7.8750E-03	6
**Arvcf**	2.2640	0.727	0.219	8.9593E-05	6
**Palmd**	2.2640	0.500	0.137	1.3889E-02	6
**Exoc6b**	2.2640	0.364	0.076	4.5964E-02	6
**Tril**	2.2628	0.545	0.163	1.2861E-02	6
**Celsr2**	2.2602	0.455	0.104	6.7038E-03	6
**Castor2**	2.2602	0.455	0.107	9.3718E-03	6
**Gm13889**	2.2516	0.682	0.242	2.8794E-03	6
**Nicn1**	2.2516	0.455	0.119	3.4845E-02	6
**Pde3a**	2.2458	0.545	0.163	1.1351E-02	6
**Tspan18**	2.2435	0.727	0.295	1.1789E-03	6
**1500011B03Rik**	2.2431	0.500	0.117	2.1700E-03	6
**Cmbl**	2.2296	0.409	0.088	1.4463E-02	6
**Cntfr**	2.2039	0.727	0.363	1.3071E-02	6
**Limk1**	2.1931	0.455	0.110	1.5754E-02	6
**Slc8a1**	2.1896	0.500	0.128	8.1557E-03	6
**C77080**	2.1888	0.545	0.164	2.3640E-02	6
**Akr1b3**	2.1849	0.636	0.213	4.5144E-03	6
**Lxn**	2.1756	0.682	0.217	5.1566E-04	6
**Zwint**	2.1548	0.773	0.338	5.9072E-04	6

Table 1

**Dpysl3**	2.1379	0.864	0.565	1.4209E-03	6
**Rusc2**	2.1349	0.955	0.301	2.8546E-08	6
**Olfml2a**	2.1108	0.591	0.207	2.0917E-02	6
**Mapre3**	2.1041	0.500	0.138	2.7154E-02	6
**Necap1**	2.0965	0.591	0.191	1.6419E-02	6
**Atp1a1**	2.0719	1.000	0.856	3.5118E-08	6
**Nipal3**	2.0686	0.636	0.257	3.6608E-02	6
**Impact**	2.0628	0.591	0.206	3.7052E-02	6
**Ndfip1**	2.0617	0.773	0.349	1.4990E-02	6
**Fabp5**	2.0494	0.727	0.366	2.7515E-02	6
**Pea15a**	2.0431	0.818	0.370	1.2728E-03	6
**Ywhag**	2.0156	1.000	0.938	1.3187E-08	6
**Scamp1**	1.9951	0.591	0.172	8.5833E-03	6
**Clstn1**	1.9817	0.909	0.564	7.4570E-05	6
**Rtn4rl1**	1.9712	0.591	0.191	2.6213E-02	6
**Gng2**	1.9521	0.545	0.147	1.0091E-02	6
**Kcnab2**	1.9521	0.636	0.214	1.2321E-02	6
**Acly**	1.9303	0.955	0.721	1.3574E-04	6
**Prkacb**	1.9034	0.955	0.683	1.2376E-03	6
**Hspa12a**	1.8891	0.545	0.138	3.7531E-03	6
**Dkk3**	1.8779	0.818	0.421	5.8288E-03	6
**Sept5**	1.8579	1.000	0.712	1.2687E-06	6
**Scd2**	1.8564	0.955	0.595	1.2859E-04	6
**Cd9**	1.8402	0.955	0.696	1.1546E-04	6
**Dlc1**	1.8385	0.682	0.263	4.7844E-02	6
**Gdi1**	1.8166	0.864	0.523	2.8755E-03	6
**Flot1**	1.7921	0.864	0.542	1.1819E-02	6
**Caml**	1.7911	0.773	0.344	7.1354E-03	6
**Gnb5**	1.7869	0.909	0.339	1.4390E-04	6
**Adam15**	1.7822	0.818	0.366	7.8577E-03	6
**Pja2**	1.7665	0.909	0.507	3.1952E-03	6
**Dynll2**	1.7657	0.955	0.733	1.5534E-04	6
**1110008P14Rik**	1.7572	0.864	0.455	2.2486E-04	6
**Rtl8b**	1.7557	0.727	0.310	4.6257E-02	6
**Map1lc3a**	1.7540	1.000	0.633	1.5512E-04	6
**Mapk3**	1.7531	0.864	0.573	3.7673E-02	6
**Serinc1**	1.7289	1.000	0.561	1.2408E-03	6
**Emb**	1.7276	0.864	0.442	4.1930E-03	6
**Evl**	1.7120	0.727	0.308	2.6156E-02	6
**S1pr3**	1.7042	0.727	0.327	2.7361E-02	6
**Col5a3**	1.6769	0.955	0.445	4.4253E-05	6
**Ywhah**	1.6451	0.864	0.608	2.5385E-02	6
**Trim2**	1.6433	0.773	0.257	2.6750E-03	6
**Cdipt**	1.6416	0.864	0.396	3.6108E-03	6

Table 1

**Nbl1**	1.6415	0.955	0.752	4.2119E-03	6
**Sparcl1**	1.6347	0.773	0.322	2.6576E-02	6
**Bex3**	1.6231	0.818	0.423	3.3939E-02	6
**Mturn**	1.6188	0.818	0.380	6.1694E-03	6
**Cd151**	1.5816	0.818	0.382	4.9542E-03	6
**Pgrmc1**	1.5370	0.909	0.577	2.7575E-02	6
**Fxyd6**	1.5242	0.818	0.342	9.5505E-03	6
**Ntn1**	1.4763	0.864	0.391	1.6027E-02	6
**Gnas**	1.4668	1.000	1.000	5.8145E-07	6
**Cndp2**	1.4624	0.818	0.370	4.7635E-02	6
**Sh3glb2**	1.4478	0.864	0.514	1.4595E-02	6
**Dctn1**	1.4375	0.864	0.612	4.7794E-02	6
**Atp6v0c**	1.4278	1.000	0.759	2.5381E-04	6
**Ppp2r1a**	1.4264	0.955	0.744	1.6968E-02	6
**Sptbn1**	1.3507	1.000	0.866	1.8730E-04	6
**Csrp1**	1.3253	1.000	0.885	1.2615E-02	6
**Selenow**	1.3123	1.000	0.850	7.4808E-03	6
**Calm1**	1.3062	1.000	0.903	2.4690E-02	6
**Aldoa**	1.2878	1.000	0.872	4.3716E-05	6
**Grina**	1.2699	1.000	0.827	1.8238E-02	6
**Dbi**	1.2458	1.000	0.880	4.4154E-03	6
**Slc25a4**	1.2362	1.000	0.988	2.5547E-07	6
**Capns1**	1.2184	1.000	0.922	1.7139E-04	6
**Cidea**	1.1513	1.000	0.700	8.9880E-03	6
**Tagln2**	1.1335	1.000	0.972	3.2549E-06	6
**Cfd**	1.1041	1.000	0.743	1.6368E-02	6
**Echs1**	1.0795	0.955	0.756	3.8399E-02	6
**Akr1a1**	1.0789	0.955	0.902	1.4180E-02	6
**Reep5**	1.0742	1.000	0.977	5.7510E-04	6
**Gpi1**	1.0684	1.000	0.993	3.6294E-03	6
**Map7d1**	1.0567	1.000	0.959	5.6128E-03	6
**Apoe**	1.0445	1.000	0.999	4.2400E-05	6
**Cox8b**	0.9935	1.000	0.941	1.5137E-02	6
**Eci1**	0.9815	1.000	0.821	8.1368E-03	6
**Tle5**	0.9304	1.000	0.974	3.9957E-02	6
**Sqstm1**	0.9262	1.000	0.968	9.2836E-04	6
**Fabp4**	0.9117	1.000	0.981	3.7690E-03	6
**Mdh1**	0.9017	1.000	0.822	3.2154E-02	6
**Ptms**	0.8372	1.000	1.000	1.5336E-03	6
**Cst3**	0.7363	1.000	1.000	8.3815E-04	6
**Ndufb10**	0.6736	1.000	0.971	4.0642E-02	6
**Itm2b**	0.5419	1.000	1.000	1.6428E-02	6

Table 2

Gene	Log Fold Change	pct.1	pct.2	Adjusted *p*-value
**Acta1**	-2.8983	0.549	0.942	1.2872E-72
**Myh2**	-3.5948	0.175	0.756	1.1795E-57
**Tnnc2**	-2.7821	0.421	0.860	1.2750E-56
**Tnnt3**	-2.4925	0.513	0.884	8.4845E-56
**Myh1**	-2.7030	0.396	0.843	1.0032E-52
**Grep1 (1520401A03Rik)**	-3.9665	0.111	0.663	5.2478E-49
**Ucp1**	2.0810	0.911	0.573	3.7154E-48
**Mylpf**	-2.2139	0.521	0.858	1.1991E-43
**Atp2a1**	-2.3434	0.292	0.770	1.2604E-42
**Myl1**	-2.4031	0.396	0.794	2.5251E-42
**Tcap**	-3.0925	0.131	0.625	4.2831E-41
**Myh7**	-3.0273	0.214	0.683	1.8419E-40
**Des**	-2.4132	0.343	0.741	4.6163E-38
**Acan**	-1.3509	0.861	0.983	5.9410E-36
**Mgp**	-1.0686	0.967	1.000	7.9550E-34
**Cox6a2**	-2.7514	0.178	0.605	5.1683E-33
**Col9a1**	-1.1187	0.986	1.000	8.1446E-33
**Ckm**	-1.7279	0.521	0.817	3.5577E-31
**Hspb7**	-3.2805	0.120	0.517	7.9228E-30
**Col2a1**	-1.1672	1.000	1.000	7.2766E-28
**Ttn**	-2.8803	0.145	0.517	1.4658E-26
**Chad**	-1.2630	0.955	0.994	9.6650E-26
**Fabp4**	0.9221	0.994	0.977	4.2635E-25
**Gpd1**	1.3517	0.877	0.625	7.3630E-25
**Col9a3**	-1.2854	0.788	0.951	1.7203E-24
**Cox8b**	1.1830	0.967	0.901	3.2910E-24
**Fhl1**	-1.7984	0.384	0.689	5.2173E-24
**Pnpla2**	1.1656	0.925	0.811	7.3225E-24
**Tpm2**	-1.5835	0.621	0.802	7.3430E-24
**Acaa2**	1.1753	0.903	0.738	8.2832E-24
**Ryr1**	-3.0041	0.058	0.390	7.3429E-23
**Cnmd**	-1.9810	0.318	0.654	1.7420E-22
**Cryab**	-1.9101	0.451	0.701	4.4251E-22
**Snorc**	-1.1760	0.630	0.863	9.0858E-22
**Comp**	-1.2238	0.989	1.000	1.2221E-21
**Actn2**	-3.0708	0.084	0.410	2.1539E-21
**Tpm1**	-0.8655	0.930	0.959	8.2389E-21
**Col9a2**	-1.4462	0.680	0.875	1.0878E-20
**Csrp3**	-3.0102	0.072	0.381	1.0757E-19
**Susd5**	-1.0750	0.638	0.858	1.1676E-19
**Tnni2**	-2.6023	0.092	0.404	3.6804E-19
**Tnnt1**	-2.9190	0.092	0.395	8.7802E-19

Table 2

**Aco2**	1.2124	0.838	0.622	1.0108E-18
**Hapln1**	-1.2555	0.577	0.817	2.0366E-18
**Eci1**	1.0394	0.883	0.709	2.4209E-18
**Apoc1**	1.2690	0.741	0.398	1.0452E-17
**Etfb**	0.8721	0.911	0.759	2.2191E-17
**Ckmt2**	-3.1194	0.039	0.305	4.8053E-17
**Tkt**	0.4863	0.981	0.985	1.4089E-16
**Casq1**	-2.2719	0.106	0.401	1.8897E-16
**Acadvl**	1.0336	0.852	0.622	1.9120E-16
**Thrsp**	1.1473	0.766	0.451	2.1627E-16
**Cox6a1**	0.6330	0.955	0.956	2.2044E-16
**Ldb3**	-2.2761	0.123	0.413	5.1355E-16
**Tnni1**	-3.4303	0.050	0.305	1.3560E-15
**Mb**	-2.9295	0.031	0.270	7.8033E-15
**Mybpc1**	-3.0516	0.053	0.305	8.0588E-15
**Clec3a**	-1.1914	0.889	0.977	8.8327E-15
**Plin1**	1.1678	0.724	0.422	8.9101E-15
**Cytl1**	-1.0070	0.805	0.927	1.1992E-14
**Lpl**	0.9475	0.847	0.637	1.2419E-14
**Matn3**	-1.5924	0.387	0.625	1.2791E-14
**Nrap**	-3.1289	0.042	0.285	1.4001E-14
**Cfd**	1.1167	0.836	0.622	2.0818E-14
**Lmod2**	-3.4081	0.033	0.265	3.9536E-14
**Sox9**	-1.1308	0.568	0.779	1.9700E-13
**Myl2**	-3.9886	0.019	0.227	3.9918E-13
**Uqcrfs1**	0.8864	0.827	0.663	5.3394E-13
**Acadl**	0.7223	0.947	0.863	1.1907E-12
**Acadm**	0.9724	0.816	0.628	2.2697E-12
**Cidea**	1.0241	0.788	0.555	2.6748E-12
**Dgat2**	0.9733	0.774	0.517	2.9244E-12
**Lipe**	0.9629	0.794	0.564	3.1289E-12
**Ifi27**	0.8521	0.838	0.613	3.3981E-12
**Adipoq**	0.7611	0.877	0.703	3.6672E-12
**Cd36**	0.7236	0.897	0.782	3.7199E-12
**Bglap**	0.5551	0.997	0.994	4.3339E-12
**Cox7a1**	1.1416	0.733	0.494	4.7828E-12
**Idh3b**	0.8304	0.833	0.616	6.8999E-12
**Atp5b**	0.5921	0.930	0.904	7.7428E-12
**Sdc4**	-0.6190	0.936	0.962	8.6512E-12
**Depp1**	1.1175	0.641	0.355	1.0058E-11
**Sparc**	-0.3979	1.000	1.000	1.0080E-11
**Atp5d**	0.5103	0.981	0.959	2.8592E-11
**Fn1**	-0.6984	0.969	0.997	5.4476E-11
**Myoz1**	-2.3515	0.064	0.285	7.5918E-11

Table 2

**Sdhb**	0.6904	0.936	0.826	1.3467E-10
**Col6a2**	-0.8857	0.869	0.980	1.3687E-10
**Bckdha**	0.9310	0.788	0.535	1.7338E-10
**Cox5b**	0.5207	0.958	0.951	1.9242E-10
**Pck1**	1.0242	0.727	0.471	2.1645E-10
**Hadh**	1.1459	0.641	0.366	2.2240E-10
**Tmsb4x**	0.2763	1.000	1.000	2.3947E-10
**Tnnc1**	-3.2310	0.033	0.224	2.6823E-10
**Tceal7**	-2.8384	0.047	0.247	3.8756E-10
**Angptl7**	-3.1271	0.095	0.308	3.9408E-10
**4931406C07Rik**	1.2354	0.493	0.206	6.1704E-10
**Cebpa**	0.8856	0.760	0.532	6.2788E-10
**Adrb3**	2.2780	0.240	0.038	6.6469E-10
**Uqcrc1**	0.5884	0.953	0.901	9.2617E-10
**Ech1**	0.7864	0.816	0.654	1.1007E-09
**Tnn**	1.3740	0.588	0.299	1.1621E-09
**Idh3a**	0.8907	0.833	0.663	1.2820E-09
**Klhl41**	-2.8052	0.042	0.233	1.6386E-09
**Adig**	1.1362	0.613	0.334	1.8341E-09
**Rsrp1**	0.4972	0.964	0.910	1.9741E-09
**Mecr**	0.9001	0.713	0.453	2.6127E-09
**Idh3g**	0.9277	0.758	0.552	3.9653E-09
**Gas1**	-1.0969	0.889	0.916	5.8208E-09
**Angptl4**	1.2495	0.404	0.142	7.2237E-09
**Kcnk3**	1.1306	0.529	0.250	8.6641E-09
**Cidec**	1.6508	0.351	0.119	1.0848E-08
**Ephx2**	1.3336	0.357	0.116	1.5900E-08
**Mdh1**	0.6540	0.869	0.733	1.5951E-08
**Txnip**	0.6320	0.875	0.709	1.6747E-08
**Actn3**	-2.2526	0.064	0.256	1.9636E-08
**Suclg1**	0.9534	0.657	0.410	2.1342E-08
**Synpo2**	-1.9141	0.156	0.363	2.1621E-08
**Hrc**	-2.1658	0.092	0.291	3.1321E-08
**Eno3**	-1.5695	0.343	0.517	3.3950E-08
**Pgam2**	-2.0681	0.081	0.282	3.5364E-08
**Gnas**	0.4362	1.000	1.000	3.7657E-08
**Decr1**	0.9189	0.688	0.413	3.7829E-08
**Idh2**	0.7231	0.813	0.645	3.8117E-08
**Cdo1**	0.9678	0.694	0.483	4.6377E-08
**Etfdh**	1.1399	0.560	0.314	7.3373E-08
**Flnc**	-2.4693	0.039	0.206	1.2906E-07
**Clu**	-1.0959	0.565	0.706	1.4429E-07
**Acads**	0.8457	0.719	0.500	1.8131E-07
**Cluh**	0.9419	0.635	0.381	1.8305E-07

Table 2

**Clstn3**	1.2892	0.359	0.128	1.9433E-07
**Sucla2**	0.7932	0.716	0.474	1.9502E-07
**Pdha1**	0.6707	0.833	0.683	1.9728E-07
**Dbi**	0.6829	0.905	0.811	1.9989E-07
**Ogdh**	0.6298	0.914	0.846	2.4404E-07
**Echs1**	0.7623	0.825	0.622	2.4783E-07
**Bgn**	-0.7203	0.961	0.971	2.5116E-07
**Ywhag**	0.5377	0.944	0.875	2.5201E-07
**Sod2**	0.7051	0.780	0.564	3.0438E-07
**Obscn**	-3.0826	0.042	0.201	3.5211E-07
**Uqcr11**	0.6067	0.866	0.741	4.4451E-07
**Slc4a4**	1.1513	0.357	0.128	5.6721E-07
**Acacb**	0.8496	0.691	0.462	5.9433E-07
**Dld**	0.7671	0.721	0.477	7.1155E-07
**Agpat3**	0.9251	0.621	0.381	7.3029E-07
**Sh2b2**	1.0214	0.485	0.233	7.3541E-07
**Frzb**	-1.4793	0.276	0.471	9.6101E-07
**Klhdc7a**	1.0226	0.407	0.166	1.0983E-06
**Cib2**	1.0005	0.485	0.233	1.2545E-06
**Ppara**	1.1076	0.412	0.174	1.5677E-06
**Cs**	0.5557	0.911	0.860	1.6084E-06
**Mcrip2**	1.0533	0.493	0.253	2.0213E-06
**Gpx3**	0.5364	0.958	0.942	2.3646E-06
**Chrac1**	1.2739	0.370	0.145	2.4766E-06
**Scd1**	0.8932	0.526	0.262	2.5885E-06
**Ndufv1**	0.5729	0.894	0.846	2.6094E-06
**Crat**	0.6525	0.783	0.608	2.7057E-06
**Jph2**	-1.1951	0.228	0.433	3.0863E-06
**Pdk4**	1.2732	0.368	0.148	3.2958E-06
**Gpd2**	1.0999	0.487	0.244	4.4210E-06
**Cyc1**	0.4858	0.942	0.860	4.6610E-06
**Ccn1**	-0.8622	0.752	0.820	5.0165E-06
**Col11a2**	-0.3523	0.989	1.000	5.4569E-06
**Atp5k**	0.5612	0.864	0.765	6.1869E-06
**Mgst1**	1.0026	0.501	0.259	6.6338E-06
**Dlst**	0.6312	0.772	0.616	6.8274E-06
**Cox7b**	0.6228	0.799	0.666	7.0568E-06
**Ces1d**	1.0602	0.348	0.131	7.2175E-06
**Myoz2**	-3.3059	0.022	0.154	7.2345E-06
**Etfa**	0.7702	0.688	0.462	7.7606E-06
**Sdhc**	0.7712	0.632	0.419	7.8271E-06
**Col6a1**	-0.7031	0.852	0.907	8.2813E-06
**Fam13a**	0.9935	0.465	0.227	8.7533E-06
**Uqcr10**	0.5384	0.864	0.776	1.0740E-05

Table 2

**Cpt1b**	0.9160	0.543	0.302	1.1657E-05
**Jsrp1**	-2.5245	0.031	0.169	1.2467E-05
**Hadha**	0.7610	0.727	0.547	1.4006E-05
**Mdh2**	0.5370	0.841	0.727	1.4838E-05
**Col27a1**	-0.8249	0.671	0.747	1.4913E-05
**Acsl1**	1.1172	0.318	0.116	1.6109E-05
**Gpam**	1.0103	0.435	0.203	1.6687E-05
**Ndufs7**	0.4816	0.900	0.808	1.6975E-05
**Trdn**	-2.5829	0.031	0.166	1.7230E-05
**Thbs4**	-1.2472	0.524	0.654	1.8406E-05
**C3**	0.6617	0.733	0.485	1.9735E-05
**Agpat2**	1.0811	0.370	0.151	2.1671E-05
**Col6a3**	-0.5566	0.947	0.983	2.2127E-05
**Hadhb**	0.8225	0.699	0.485	2.2635E-05
**Ndufa4l2**	-1.0812	0.521	0.637	2.5588E-05
**B2m**	0.4052	0.933	0.852	2.7725E-05
**Apobec2**	-3.1746	0.014	0.131	2.9212E-05
**Pvalb**	-0.8015	0.390	0.581	3.2605E-05
**Cpt2**	1.1157	0.415	0.201	3.2699E-05
**Klhl31**	-2.7985	0.019	0.142	3.3533E-05
**Ctsb**	0.2772	0.972	0.977	3.4164E-05
**S100a9**	0.3301	1.000	1.000	3.4688E-05
**Slc25a42**	1.2113	0.393	0.192	3.4705E-05
**Nrg4**	0.9801	0.354	0.145	4.0400E-05
**Lgals3**	-0.6055	0.830	0.840	4.4235E-05
**Meltf**	-1.0050	0.337	0.517	4.9159E-05
**Zic1**	1.7741	0.192	0.044	5.2077E-05
**Pank1**	1.1122	0.362	0.157	7.8761E-05
**Hspb6**	-1.4944	0.231	0.395	8.8068E-05
**Ndufs8**	0.5194	0.880	0.765	8.9365E-05
**Cntfr**	0.9240	0.493	0.270	9.1825E-05
**Slc25a20**	0.7691	0.549	0.320	9.9612E-05
**Ahcyl1**	0.5903	0.805	0.628	9.9628E-05
**Pakap**	0.8939	0.546	0.337	1.0930E-04
**Mat2b**	0.7281	0.577	0.337	1.1533E-04
**Abi3bp**	-1.0676	0.496	0.613	1.2050E-04
**Grem1**	-1.2901	0.290	0.453	1.3604E-04
**Tspan18**	0.8589	0.440	0.212	1.3641E-04
**Prkar2b**	0.6181	0.733	0.517	1.3969E-04
**Chpt1**	0.8062	0.593	0.395	1.4224E-04
**Tob2**	0.7290	0.521	0.288	1.5836E-04
**Ankrd2**	-3.5464	0.011	0.116	1.6254E-04
**Ghitm**	0.6121	0.794	0.608	1.6281E-04
**S100a1**	0.7212	0.724	0.544	1.6375E-04

Table 2

**Acsm3**	1.0381	0.435	0.218	1.7300E-04
**Stmn2**	2.3459	0.312	0.134	1.8346E-04
**Plin5**	1.2445	0.223	0.064	1.8429E-04
**Bri3**	0.8610	0.513	0.291	1.8585E-04
**Cst3**	-0.3049	1.000	1.000	1.9885E-04
**Atp5a1**	0.3735	0.969	0.968	1.9940E-04
**Cyp4b1**	0.9906	0.290	0.108	2.0379E-04
**Otop1**	1.2435	0.267	0.096	2.1345E-04
**Slc36a2**	0.8253	0.680	0.515	2.7302E-04
**Xirp1**	-3.0297	0.019	0.131	2.7356E-04
**Cox17**	0.4808	0.847	0.674	2.8792E-04
**Laptm5**	0.5079	0.844	0.628	3.1061E-04
**Dlat**	0.5730	0.747	0.570	3.1277E-04
**Mlec**	0.5906	0.719	0.506	3.2143E-04
**Sptbn1**	0.4994	0.883	0.785	3.3128E-04
**Fmod**	-0.8496	0.713	0.817	4.1348E-04
**Rnf41**	0.7559	0.376	0.163	4.3431E-04
**Myh3**	-3.0886	0.017	0.122	4.6468E-04
**1700037H04Rik**	0.7204	0.621	0.381	4.7325E-04
**Chchd10**	0.6528	0.735	0.561	5.1164E-04
**Calml3**	-1.6559	0.120	0.276	5.1616E-04
**Acat1**	0.6939	0.666	0.445	5.2066E-04
**Hsd17b12**	0.7424	0.646	0.430	5.4052E-04
**Gpi1**	0.2994	0.964	0.965	5.4314E-04
**Syt4**	3.1643	0.120	0.015	6.1026E-04
**Ntrk3**	0.7950	0.379	0.169	6.2170E-04
**Pparg**	1.4666	0.270	0.105	6.5211E-04
**Nt5c3**	0.8253	0.568	0.337	6.7569E-04
**Thra**	0.8167	0.418	0.195	6.8271E-04
**Lrrc15**	1.3288	0.234	0.078	7.4184E-04
**Acly**	0.6111	0.791	0.616	7.5280E-04
**Pdlim3**	-2.2304	0.056	0.186	7.7495E-04
**Smdt1**	0.4564	0.833	0.663	8.1344E-04
**Pcx**	0.7586	0.588	0.363	8.3698E-04
**Letmd1**	0.7628	0.468	0.247	8.5389E-04
**Acad11**	0.9423	0.409	0.206	8.7341E-04
**Cox8a**	0.3891	0.950	0.898	8.8670E-04
**Fh1**	0.7067	0.685	0.500	9.3888E-04
**Mgll**	0.5568	0.758	0.584	9.4554E-04
**Gpt**	1.0694	0.354	0.163	9.9880E-04
**Myo18b**	-2.4052	0.036	0.154	1.0105E-03
**Pdk2**	0.6402	0.604	0.378	1.0686E-03
**Ndufa10**	0.6893	0.652	0.465	1.0884E-03
**Hspa9**	0.4202	0.905	0.849	1.1001E-03

Table 2

**Gata2**	2.1397	0.142	0.026	1.1498E-03
**Pcsk1n**	2.3056	0.237	0.084	1.1681E-03
**Slc25a34**	1.9722	0.136	0.023	1.1965E-03
**Slc24a3**	1.1284	0.267	0.102	1.2033E-03
**Ppargc1b**	0.7311	0.499	0.282	1.2197E-03
**Gpihbp1**	0.7788	0.476	0.270	1.3915E-03
**Pgam1**	0.3789	0.908	0.831	1.4468E-03
**Ndufb6**	0.4097	0.928	0.872	1.4590E-03
**Adipor2**	0.9435	0.320	0.140	1.4971E-03
**Art1**	-2.8607	0.019	0.122	1.5555E-03
**Wwp2**	-0.5516	0.783	0.805	1.6180E-03
**Mcam**	1.1288	0.337	0.148	1.6240E-03
**Bckdhb**	0.7382	0.426	0.212	1.6567E-03
**Gm13889**	0.9424	0.368	0.177	1.7822E-03
**Thbs1**	-0.4454	0.900	0.939	2.2180E-03
**Aco1**	0.6746	0.460	0.241	2.2481E-03
**Tagln2**	0.3399	0.942	0.913	2.3270E-03
**Aifm2**	0.8351	0.396	0.195	2.5343E-03
**Rgs2**	0.6834	0.607	0.395	2.6903E-03
**Aoc3**	0.8118	0.415	0.209	2.8687E-03
**Pygm**	-1.5821	0.142	0.282	2.8854E-03
**Plxnd1**	0.4904	0.788	0.660	3.3199E-03
**Acot13**	0.6117	0.390	0.186	3.3603E-03
**Tspo**	0.4039	0.903	0.863	3.3785E-03
**Immt**	0.5516	0.758	0.593	3.4402E-03
**Aldh4a1**	0.9639	0.340	0.157	3.5132E-03
**Sdha**	0.6257	0.674	0.517	3.5337E-03
**Coq8a**	0.5503	0.802	0.651	3.8529E-03
**Mtfp1**	1.1285	0.259	0.102	4.4333E-03
**Rtn3**	0.7856	0.526	0.305	4.4684E-03
**Hsp90b1**	0.2503	0.997	0.974	4.6106E-03
**Aldoa**	0.4788	0.886	0.805	4.8100E-03
**Pxmp2**	1.0195	0.209	0.067	4.8253E-03
**Prph**	2.1843	0.348	0.177	4.8326E-03
**Ndufab1**	0.6033	0.708	0.529	4.9939E-03
**Tpt1**	0.3044	0.978	0.924	5.3047E-03
**Scrg1**	-1.0779	0.309	0.456	5.3680E-03
**Card19**	0.7464	0.468	0.259	5.6603E-03
**Creb3l2**	-0.6907	0.630	0.703	5.6732E-03
**Dpep1**	1.3359	0.175	0.049	5.9951E-03
**Kcnab2**	0.9655	0.329	0.154	6.0226E-03
**Clybl**	0.8380	0.376	0.183	6.4090E-03
**Slc27a1**	0.5564	0.476	0.253	6.5148E-03
**Cyp27a1**	0.8190	0.334	0.157	6.8386E-03

Table 2

**Fmc1**	0.6468	0.604	0.401	7.2640E-03
**Wif1**	-0.7495	0.724	0.779	7.3370E-03
**Sgcg**	-2.0142	0.028	0.131	7.3493E-03
**Sfxn1**	0.6044	0.646	0.427	7.4481E-03
**Aldh6a1**	0.5750	0.696	0.483	7.5857E-03
**2310061I04Rik**	0.8079	0.432	0.233	8.2379E-03
**Grap**	1.1575	0.228	0.084	8.3473E-03
**Slc22a3**	1.6352	0.153	0.038	8.3656E-03
**H2-DMa**	0.9124	0.362	0.174	8.7452E-03
**Ak3**	0.5954	0.460	0.250	8.8514E-03
**Cat**	0.4137	0.866	0.753	9.5508E-03
**Trim54**	-2.1747	0.042	0.151	9.9521E-03
**Hand2**	2.6150	0.203	0.076	1.0741E-02
**Dlk1**	-1.0041	0.393	0.517	1.1287E-02
**Capn7**	1.4826	0.162	0.044	1.1795E-02
**Srsf5**	0.5229	0.758	0.535	1.1867E-02
**Vip**	3.0704	0.145	0.038	1.2332E-02
**Acot8**	0.6248	0.376	0.183	1.2413E-02
**Sbsn**	-1.6990	0.056	0.174	1.2483E-02
**Cavin2**	0.5358	0.699	0.515	1.3228E-02
**Fxyd6**	-1.2065	0.323	0.459	1.4601E-02
**Egfl7**	0.5719	0.552	0.349	1.4936E-02
**Ddt**	0.6510	0.532	0.326	1.5576E-02
**C030006K11Rik**	0.7629	0.390	0.206	1.5773E-02
**Pts**	0.6571	0.373	0.180	1.6021E-02
**Col10a1**	-0.7822	0.560	0.657	1.6027E-02
**Slc1a5**	0.3484	0.925	0.811	1.6471E-02
**Vps13d**	0.8047	0.415	0.218	1.6631E-02
**Alpk3**	-2.0080	0.053	0.166	1.6643E-02
**Cyyr1**	0.9842	0.212	0.076	1.7219E-02
**Sncg**	1.7022	0.309	0.145	1.7401E-02
**Lmcd1**	-1.2930	0.173	0.311	1.8654E-02
**Cd300lg**	0.6675	0.485	0.270	1.8796E-02
**Sptan1**	0.6405	0.624	0.439	1.8878E-02
**Hk2**	0.7864	0.337	0.160	1.9753E-02
**Anxa8**	-1.0719	0.370	0.480	2.0075E-02
**Sfrp1**	1.7525	0.156	0.044	2.0383E-02
**Prelp**	-0.8248	0.900	0.951	2.0613E-02
**Megf6**	1.0538	0.220	0.081	2.0761E-02
**Ralgapa2**	0.8105	0.345	0.169	2.0912E-02
**Sypl2**	-2.5760	0.031	0.128	2.0979E-02
**Adcy3**	0.9126	0.290	0.128	2.1605E-02
**Aqp7**	0.7806	0.390	0.203	2.2046E-02
**Slc2a1**	-1.1009	0.415	0.515	2.2140E-02

Table 2

**Myom2**	-2.3098	0.025	0.119	2.3589E-02
**Myl3**	-3.0310	0.017	0.102	2.3717E-02
**Eng**	0.5866	0.666	0.471	2.3866E-02
**Pirb**	0.7130	0.493	0.302	2.3995E-02
**Zfp865**	1.0652	0.234	0.093	2.4031E-02
**Adgrg3**	0.9941	0.195	0.067	2.4693E-02
**Bcl6**	0.6754	0.393	0.203	2.6003E-02
**Pttg1**	0.6454	0.652	0.448	2.7218E-02
**Atox1**	0.3950	0.830	0.677	2.7735E-02
**Fasn**	0.7691	0.340	0.169	2.8497E-02
**Pcsk6**	-0.7853	0.552	0.613	3.0065E-02
**Elovl1**	0.6581	0.460	0.250	3.0361E-02
**Ccdc25**	0.4828	0.309	0.140	3.0966E-02
**Ndufb8**	0.5153	0.797	0.698	3.1379E-02
**Rerg**	1.1331	0.203	0.073	3.1718E-02
**Chst11**	-0.7917	0.507	0.616	3.2153E-02
**Ccr7**	1.0325	0.175	0.055	3.2375E-02
**Ptges2**	0.6351	0.409	0.221	3.2507E-02
**Acaa1b**	1.6122	0.153	0.044	3.3342E-02
**Mt1**	0.3734	0.919	0.846	3.4885E-02
**Phb2**	0.3962	0.802	0.593	3.5693E-02
**Mcur1**	0.7498	0.535	0.343	3.6146E-02
**Eif4e3**	1.0197	0.276	0.119	3.6390E-02
**Vim**	-0.4171	0.986	0.997	3.6579E-02
**Dbt**	0.8503	0.323	0.157	3.8527E-02
**Gsn**	-0.5231	0.894	0.922	3.8902E-02
**T**	-1.2969	0.061	0.177	3.9039E-02
**Rnf152**	0.7130	0.217	0.078	3.9463E-02
**Ntrk1**	2.3464	0.150	0.044	4.0634E-02
**Lrg1**	0.5478	0.682	0.480	4.1402E-02
**Fabp3**	0.7098	0.652	0.506	4.2087E-02
**Gngt2**	0.6148	0.496	0.285	4.2208E-02
**Tinagl1**	0.6424	0.496	0.291	4.2442E-02
**Tmem70**	0.7816	0.253	0.105	4.2603E-02
**Plaat3**	0.4733	0.657	0.451	4.2702E-02
**Acox1**	0.7020	0.432	0.244	4.2995E-02
**Col15a1**	-0.8043	0.671	0.718	4.3597E-02
**Ehhadh**	0.9237	0.259	0.110	4.4174E-02
**Slc2a8**	0.8366	0.220	0.084	4.5398E-02
**Ctsz**	0.4652	0.791	0.645	4.6173E-02
**Tpd52**	0.9813	0.295	0.140	4.6468E-02
**Ttc38**	0.6385	0.368	0.186	4.6609E-02
**Cilp2**	-0.8460	0.808	0.797	4.6799E-02
**Ndufb9**	0.5602	0.582	0.369	4.8966E-02

Table 2

**Gsta4**	0.9078	0.220	0.084	4.9105E-02
**A530016L24Rik**	1.4735	0.148	0.041	4.9286E-02
**Tcim**	0.6436	0.404	0.224	4.9404E-02
**Zcchc9**	0.7426	0.429	0.244	4.9756E-02
**Gap43**	2.0146	0.159	0.049	5.0808E-02

Table 3

Genotype	*Sample ID*	*P40 Cobb Angle*	*P120 Cobb Angle*
*Col2Cre;*Gpr126$^{f/f}$;Sox9$^{del/del}$	DM_1	38	26
	DM_3	43	25, 25
	F18_3	No curve	13.2
	F21_7	29	16.4
*Col2Cre;*Gpr126$^{f/f}$;Sox9$^{del/del}$	F23_4	26	30.2
*Col2Cre;*Gpr126$^{f/f}$;Sox9$^{del/del}$	F46_3	No curve	15.6
*Col2Cre;*Gpr126$^{f/f}$;Sox9$^{del/del}$	F51_5	No curve	26
*Col2Cre;*Gpr126$^{f/f}$;Sox9$^{del/del}$	F59_1	23.3	25.5
*Col2Cre;*Gpr126$^{f/f}$;Sox9$^{del/+}$	F18_1	No curve	17.2
*Col2Cre;*Gpr126$^{f/f}$;Sox9$^{del/+}$	F26_2	45, 77	63.3, 89.1
*Col2Cre;*Gpr126$^{f/f}$;Sox9$^{del/+}$	F28_7	No curve	15.5, 25.4
*Col2Cre;*Gpr126$^{f/f}$	E22_1	14	28
*Col2Cre;*Gpr126$^{f/f}$	E22_3	No curve	15
